# Multimolecular characteristics and role of BRCA1 interacting protein C-terminal helicase 1 (BRIP1) in human tumors: a pan-cancer analysis

**DOI:** 10.1186/s12957-022-02877-8

**Published:** 2023-03-13

**Authors:** Ruohuang Wang, Jisheng Zhang, Xin Cui, Shun Wang, Ting Chen, Yanfang Niu, Xiaoyun Du, Jingwen Kong, Lin Wang, Yan Jiang

**Affiliations:** 1 Department of Otolaryngology-Head and Neck Surgery, The Affiliated Hospital of Qingdao University, Qingdao University, Qingdao, Shandong 266000 China; 2 Qingdao Women and Children’s Hospital, Qingdao University, Qingdao, Shandong 266000 China; 3 The Ninth People’s Hospital Affiliated to Shanghai Jiaotong University School of Medicine, Shanghai, 200000 China; 4 Department of Clinical Laboratory, Yuncheng Central Hospital, Yuncheng, Shanxi 044000 China

**Keywords:** BRIP1, Tumor, Expression, Prognosis, Alteration, Methylation

## Abstract

**Background:**

The aberrant expression of BRIP1 was associated with several cancers; however, the panoramic picture of BRIP1 in human tumors remains unclear. This study aims to explore the pan-cancerous picture of the expression of BRIP1 across 33 human cancers.

**Methods:**

Based on the data from TCGA and GTEx, a series of bioinformatic analyses were applied to systematically explore the genetic landscape and biologic function of BRIP1 in 33 human tumors.

**Results:**

We observed prognosis-related differential BRIP1 expressions between various carcinomas and the corresponding normal tissues. “Basal transcription factors,” “homologous recombination,” “nucleotide excision repair,” and DNA metabolism pathways may play a role in the functional mechanisms of BRIP1. Patients with uterine corpus endometrial carcinoma presented with the highest alteration frequency of BRIP1 (nearly 10%). Single-nucleotide and copy number variations of BRIP1 were noticed in multiple cancers, and the expression of BRIP1 is significantly regulated by copy number variation in breast invasive carcinoma and lung squamous cell carcinoma. BRIP1 expression is negatively correlated with the DNA methylation levels in many tumors and is associated with the activation of apoptosis, cell cycle, DNA damage response, and inhibition of hormone ER and RNS/MARK signaling pathways. Moreover, a positive correlation was observed between BRIP1 expression and the immune infiltration levels of cancer-associated fibroblasts and CD8+ T cells in lung adenocarcinoma.

**Conclusion:**

Our pan-cancer analysis of BRIP1 provides a valuable resource for understanding the multimolecular characteristics and biological function of BRIP1 across human cancers.

**Supplementary Information:**

The online version contains supplementary material available at 10.1186/s12957-022-02877-8.

## Background

Over the past decade, the incidence and mortality of tumors have continued to rise [1]. A recent study based on data of 38 cancers from 185 countries reported more than 19 million new cancer cases worldwide in 2020, and nearly half of these patients died because of cancer [1]. Many studies have been taken to explore the potential molecular mechanisms and factors of tumor carcinogenesis, development, prognosis, and treatment response [2–8]. The tumor microenvironment (TME) [9], mutations [10], epigenetics [11], and immune microenvironment [12] are some of the identified factors that are significantly associated with the malignant behavior of cancers.

As one of the *DEAH* helicase family members, BRCA1 interacting protein C-terminal helicase 1 (BRIP1), also known as BRCA1-associated C-terminal helicase-1 (BACH1), consists of 20 exons and is located at 17q23 chromosome [13]. BRIP1 was linked to Fanconi anemia (FA), an autosomal recessive genetic disease characterized by cancer susceptibility, bone marrow failure, and multiple physical abnormalities [14]. Since mutations of BRIP1 were noticed in patients of Fanconi anemia who belong to the complementation group J, BRIP1 was also named FANCJ [15]. With respect to the physiology of BRIP1, direct interaction between BRCA1 and BRIP1 was detected. Such interaction is facilitated by a vital domain of BRCA1 (BRCT), which is essential for establishing the G2 cell cycle when responding to DNA damage [16]. Besides, PALB2 (FANCN), BRCA2, and BRIP1 are three crucial genes that worked commonly in the FA-BRCA pathway and acted downstream of FANCD2. The details of these interactions are shown in Figure S1. Meanwhile, BRIP1’s helicase domains were suggested to directly contribute to DNA repairing sites [17]. Recently, the association between BRIP1 and cancers has been reported; however, most studies were carried out on ovarian, breast, and pancreatic cancer, and most of these analyses mainly focused on identifying the mutation effects on cancers [13, 18, 19]. The genetic landscape and biological function of BRIP1 across pan-cancer remain unclear.

In the current study, we performed a pan-cancer analysis of BRIP1 in various human cancers using multiple online databases to explore the impending mechanisms of BRIP1 in the carcinogenesis, development, and clinical outcomes of various human tumors. The design and summarization of the current study are shown in Figure S2.

## Materials and methods

### Gene expression analysis

We utilized the ONCOMINE database [20] (www.oncomine.org) to explore the differences in BRIP1 expression between normal and tumor tissues under the settings of fold change = 1.5, *P* value = 0.05. We recoded the statistically significant datasets and performed a series of pooling analyses across multiple comparisons.

We also logged into the TIMER2 [21] (http://timer.cistrome.org/) website to analyze the differential BRIP1 expression between cancer and corresponding normal tissues across 33 human carcinomas by inputting BRIP1 in the “Gene-DE” module. Differences in BRIP1 expression were compared using the Wilcoxon test. Since the data of normal tissues in the TIMER2 database is unavailable for several cancers [e.g., SARC (Sarcoma), THYM (Thymoma)], we using the GEPIA2 [22] (http://gepia2.cancer-pku.cn/#analysis) database to evaluate the differential expression levels of BRIP1 between these tumors and their corresponding normal tissues. The cutoff points were set as “Match TCGA normal and GTEx data”, *P* value = 0.05, and log2FC (fold change) = 1, and the expression levels were displayed with box plots. In addition, associations between BRIP1 expression and the pathological stage of various cancers were also investigated.

To examine the differential proteins expression of BRIP1 among various tumors, we logged into The Human Protein Atlas (HPA) [23] (https://www.proteinatlas.org) website to compare the BRIP1 protein expression between breast cancer, lung cancer, colorectal cancer, liver cancer, prostate cancer, and their corresponding normal tissues. The data on BRIP1 expression in different tumors, various normal tissues, and blood cells were also retrieved.

### Survival analysis

Using the GEPIA2 database, we also compared the survival contribution of BRIP1 in all TCGA tumors by entering “BRIP1” in the “Survival Map” module, estimated using the Mantel-Cox test. The significance map of overall survival (OS) and disease-free survival (DFS) was generated under the significance threshold *P* value of 0.05. The BRIP1 cases were divided into the high and low expression group based on the median expression level of BRIP1. Therefore, the cases with the BRIP1 expression higher or lower than the cutoff value were considered as the high or low BRIP1 group. The survival analysis was performed between the two groups. Meanwhile, for the cancer types with statistical significance of OS and DFS, we also obtained the Kaplan-Meier curves under the “Survival Analysis” module of GEPIA2.

### BRIP1-related gene enrichment analysis

Firstly, we logged into the STRING [24] (https://string-db.org/) website to obtain the BRIP1-binding proteins that were experimentally determined by entering “BRIP1” under the single protein name module, and “Homo sapiens” was chosen as the organism. In the “Settings” section, we set the following filters: “full network” as the network type, “evidence” as the meaning of network edges, “Experiments” as the active interaction sources, “low confidence (0.15)” as the minimum required interaction score, and “no more than 50 interactors” as the max number of interactors to show. A protein-protein interaction (PPI) analysis was conducted among the selected 50 genes. The interaction between genes was analyzed by the STRING database and visualized by Cytoscape.

Secondly, we utilized the “Similar Gene Detection” function of GEPIA2 to get the top 100 genes with the highest correlation with BRIP1. For the top five BRIP1-correlated genes, we also performed a Pearson correlation analysis between BRIP1 and each selected correlated gene using the GEPIA2 database under the “correlation analysis” module. Moreover, the heatmap data of the five genes were generated using the “Gene_Corr” module of the TIMER2 database.

Thirdly, we utilized Venn online website (http://bioinformatics.psb.ugent.be/webtools/Venn/) to compare the BRIP1-interacted and binding genes by conducting an intersection analysis. Additionally, we also combined the two sets of genes to conduct Kyoto Encyclopedia of Genes and Genomes (KEGG) and Gene Oncology (GO) enrichment analysis in the database of Metascape [25]. There are three components contained in GO analysis, molecular functions (MF), cellular components (CC), and biological processes (BP), which could predict the functional roles of genes closely related to BRIP1, while KEGG analysis could delineate the pathways of the genes related to BRIP1.

### Mutation, methylation, and **g**enome-wide association of BRIP1 mRNA in cancers

By logging into the cBioPortal [26] (http://www.cbioportal.org) website, the copy number alteration (CNA), mutation types, and alteration frequency of BRIP1 across different cancers can be observed under the “TCGA Pan-Cancer Atlas Studies” module, in the “Cancer Types Summary” section. The mutation sites and three-dimensional (3D) structure of BRIP1 can be obtained in the “Mutation” section. In the “Comparison/Survival” section, for a specific TCGA cancer type, we can explore the correlation between gene alteration and the survival of specific cancers, including disease-specific, progress-free, overall, and disease-free survival. The results were displayed as Kaplan-Meier plots, and a log-rank test *P* value was supplied.

We utilized the MEXPRESS [27] (https://mexpress.be/) database to explore the association of BRIP1 expression and DNA methylation across all TCGA tumors, only cancer types with statistical significance were displayed. The methylation level of each probe was presented with a beta value, and the adjusted *P* value (Benjamini-Hochberg) and the Pearson correlation coefficient value (R) were provided.

We also logged into GSCALite [28] (http://bioinfo.life.hust.edu.cn/web/GSCALite/) website to evaluate the correlation between DNA methylation and the expression of BRIP1 and its five most correlated genes across 33 TCGA tumors. The results were displayed as bubble maps. Besides methylation data, GSCALite provides information on the SNV (single-nucleotide variation) and CNV (copy number variation) of BRIP1 and its five most related genes in all TCGA tumors. In addition, Pearson correlation was performed to explore the association between genes’ expression and CNV of each cancer, which could help to investigate the influence of CNV on the expression of selected genes. Additionally, the differences of BRIP1 and the five most related genes’ expression between pathway activation and inhibition groups across all TCGA tumors were explored.

Moreover, we used the Cancer Regulome tools (http://explorer.cancerregulome.org/) to evaluate the association of BRIP1 expression with other genes in various tumors. The correlations were displayed as circus diagrams based on the links between Protein level-RPPA, somatic mutation, microRNA expression, somatic copy number, DNA methylation, and gene expression. Besides, using the Sangerbox tool, we investigated the association between gene expression of BRIP1 and marker genes of RNA modification in various cancers.

### Immune infiltration analysis

Using the TIMER2 database, we explored the correlation between the expression and immune infiltrates of BRIP1 in different cancers. We selected the cancer-associated fibroblasts and CD8+ T cells for immune infiltration estimation, using the algorithms of EPIC, MCPCOUNTER, QUANTISEQ, CIBERSORT-ABS, CIBERSORT, XCELL, and TIMER. The associations were displayed as heatmap and scatter plots, and purity-adjusted *P* value and partial correlation (cor) were supplied.

## Results

### Expression analysis of BRIP1

In the current work, the genetic landscape and biological function of BRIP1 (genome location: chr7(q32.1), consensus CDS: CDS11631.1, Figure S3a) across human cancers were investigated. As presented in Figure S3b, a conserved domain of DEAD_2 (pfam06733) commonly consists of BRIP1 protein structure among different species. The evolutionary relationship of the BRIP1 protein among various species was presented in a phylogenetic tree (Figure S4).

**Fig. 1:**
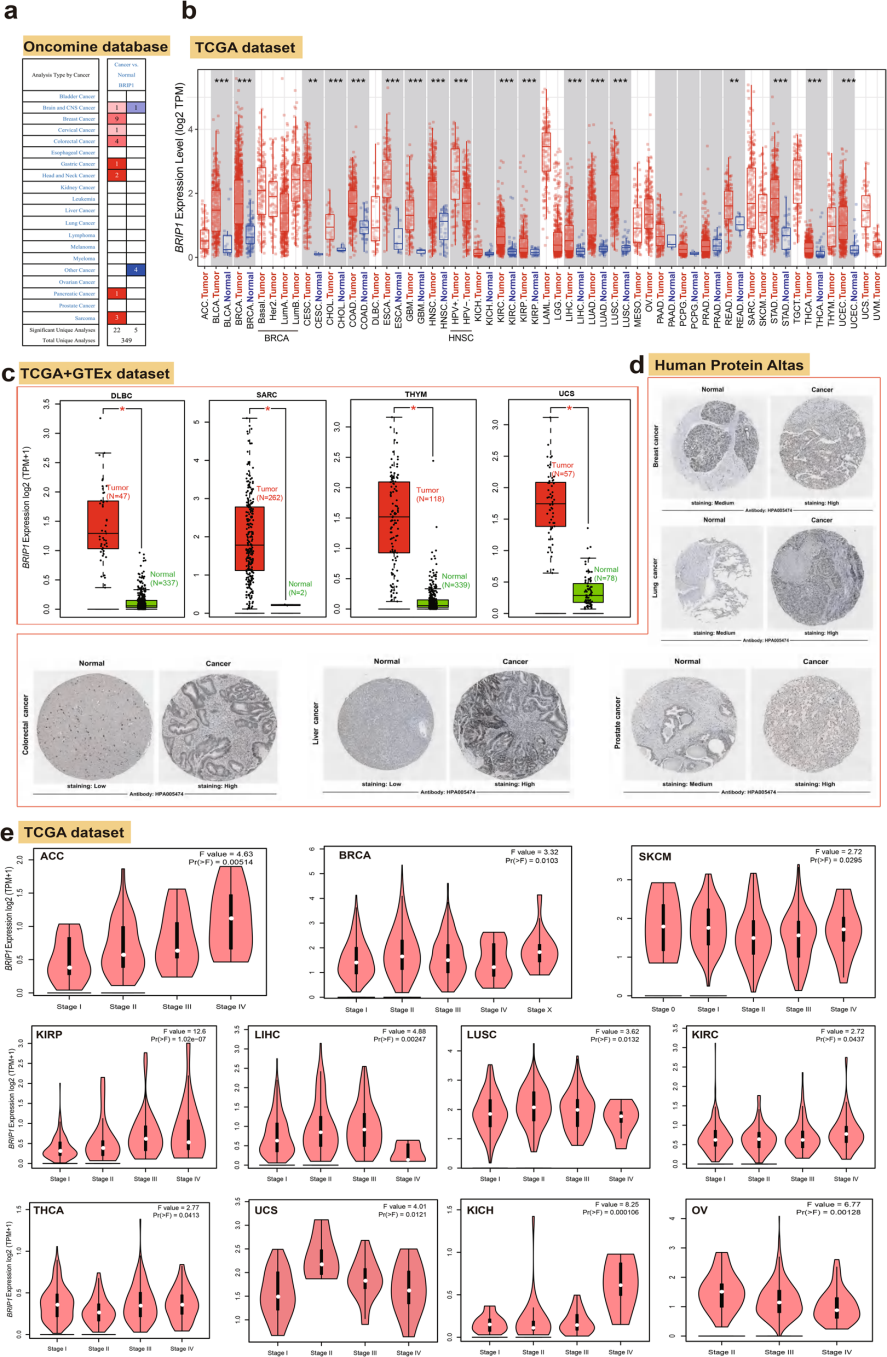
The expression levels of BRIP1 gene in different tumors and pathological stages. **a** The transcription levels of BRIP1 in different types of human cancers (ONCOMINE). The cell number represents the dataset number that meets all the thresholds with the color blue for under-expression and color red for over-expression. Cell color is determined by the best gene rank percentile for the analyses within the cell. **b** The expression status of the BRIP1 gene in different cancers or specific cancer subtypes was analyzed through TIMER2. *P<0.05; **P<0.01; ***P<0.001. **c** For the type of DLBC, SARC, THYM, and UCS in the TCGA project, the corresponding normal tissues of the GTEx database were included as controls. The box plot data were supplied. *P <0.05. **d** Representative immunohistochemistry images of BRIP1 in breast cancer, lung cancer, colorectal cancer, liver cancer, prostate cancer, and their corresponding normal tissues (Human Protein Atlas). **e** Based on the TCGA data, the expression levels of the BRIP1 gene were analyzed by the main pathological stages (stage I, stage II, stage III, and stage IV) of ACC, BRCA, SKCM, KIRP, LIHC, LUSC, KIRC, THCA, UCS, KICH, and OV. Log2 (TPM+1) was applied for the log-scale

We first used the ONCOMINE database to compare BRIP1 mRNA expression between tumors and corresponding normal tissues (Fig. 1a). Higher expression of BRIP1 was noticed in brain and CNS cancer, breast cancer, cervical cancer, colorectal cancer, gastric cancer, head and neck cancer, pancreatic cancer, and sarcoma than in the corresponding normal tissues. However, significantly downregulated expression of BRIP1 was also noticed in several tumors. The details are shown in Table S1. Collectively, 22 datasets showed a higher mRNA expression of BRIP1 in different tumors than in normal samples, while five datasets showed controversial results. We then assessed the levels of BRIP1 protein expression across different cancers in datasets from the HPA database. As shown in Figure S5a, most tumor tissues showed moderate to strong nuclear or nuclear membranous staining, especially in colorectal cancer, head and neck cancer, carcinoid, urothelial cancer, prostate cancer, and melanoma. The highest expression of BRIP1 was identified in the thymus, followed by testis and bone marrow (Figure S5b). Similarly, BRIP1 expression was detected in all blood cells, and low RNA blood cell type specificity was noticed with the consensus datasets of HPA, Monaco, and Schmiedel (Figure S5c). While in brain tissues, the highest expression of BRIP1 was found in the basal ganglia, followed by the cerebral cortex and olfactory region (Figure S5d).

Figure 1b showed higher BRIP1 expressions in cancer tissues of BLCA (bladder urothelial carcinoma), BRCA (breast invasive carcinoma), CHOL (cholangiocarcinoma), COAD (colon adenocarcinoma), ESCA (esophageal carcinoma), GBM (glioblastoma multiforme), HNSC (head and neck squamous cell carcinoma), KIRC (kidney renal clear cell carcinoma), KIRP (kidney renal papillary cell carcinoma), LIHC (liver hepatocellular carcinoma), LUAD (lung adenocarcinoma), LUSC (lung squamous cell carcinoma), STAD (stomach adenocarcinoma), THCA (thyroid carcinoma), UCEC (uterine corpus endometrial carcinoma) (*P*<0.001), CESC (cervical squamous cell carcinoma), and READ (rectum adenocarcinoma) (*P*<0.01) than in the corresponding normal tissues. In summary, a total of 21 cancer types with data of normal samples were investigated in the TIMER2 database. The upregulation of BRIP1 was found in 17 cancers, and no statistical differences exist in 4 cancers (Fig. 1b). As there are several cancer types in TIMER2 lacking the data of normal tissues, we used the GEPIA2 database to compare the differences of BRIP1 expression between corresponding normal tissues and cancer tissues of DLBC (lymphoid neoplasm diffuse large B cell lymphoma), SARC (sarcoma), THYM (thymoma), and UCS (uterine carcinosarcoma), and higher BRIP1 expressions were noticed in the cancer tissues (Fig. 1c, *P*<0.05). However, no statistical significance was detected for other cancers (Figure S6a). Besides, the results of pooling analysis in the ONCOMINE database also verified that BRIP1 is highly expressed in breast, sarcoma, colorectal, and head and neck cancers (Figure S7). Additionally, the correlation between BRIP1 expression and cancers with different pathological stages was investigated using the “Pathological Stage Plot” module of GEPIA2. Significantly differences were found in ACC (adrenocortical carcinoma), BRCA, SKCM (skin cutaneous melanoma), KIRP, LIHC, LUSC, KIRC, THCA, UCS, KICH (kidney chromophobe), and OV (ovarian serous cystadenocarcinoma) (Fig. 1e). Cancers without significance were shown in Figure S6b-d.

Moreover, we analyzed BRIP1 expression in different molecular and immune subtypes. As shown in Figure S8a, significantly different BRIP1 expression was observed in various molecular subtypes of BRCA, LGG, PCPG (pheochromocytoma and paraganglioma), COAD, LUSC, STAD, HNSC, KIRP, OV, UCEC (*P*<0.001 for all), LIHC (*P*<0.01), SKCM, and ESCA (*P*<0.05 for all). Figure S8b showed that significant differences in BRIP1 expression exist across immune subtypes of C1 to C6 (represent wound healing, IFN-γ dominant, inflammatory, lymphocyte deplete, immunologically quiet, and TGF-β dominant, respectively) in BLCA, LGG, SARC, BRCA, LUAD, SKCM, COAD, LUSC, STAD, ESCA, OV, THCA, KICH, PCPG, UCEC, KIRC, READ, and KIRP. Of interest, the lowest BRIP1 expression was noticed in subtype C3 in most cancers, except for LGG and KIRC (BRIP1 expression in C5 is the lowest). Cancer types with no significant difference were presented in Figure S9. Jointly, the differential expression of BRIP1 in various molecular and immune subtypes may contribute to the differing role of BRIP1 in the prognosis of different tumors.

After exploring the differential expression patterns of BRIP1 between tumors and normal tissues, we also used the HPA dataset to examine the protein expression patterns of BRIP1 in breast cancer, lung cancer, colorectal cancer, liver cancer, and prostate cancer. As shown in Fig. 1d, high expression of BRIP1 was found in the tumor tissues, whereas low to medium expression of BRIP1 was noticed in the corresponding normal tissues.

### Survival analysis of BRIP1

**Fig. 2:**
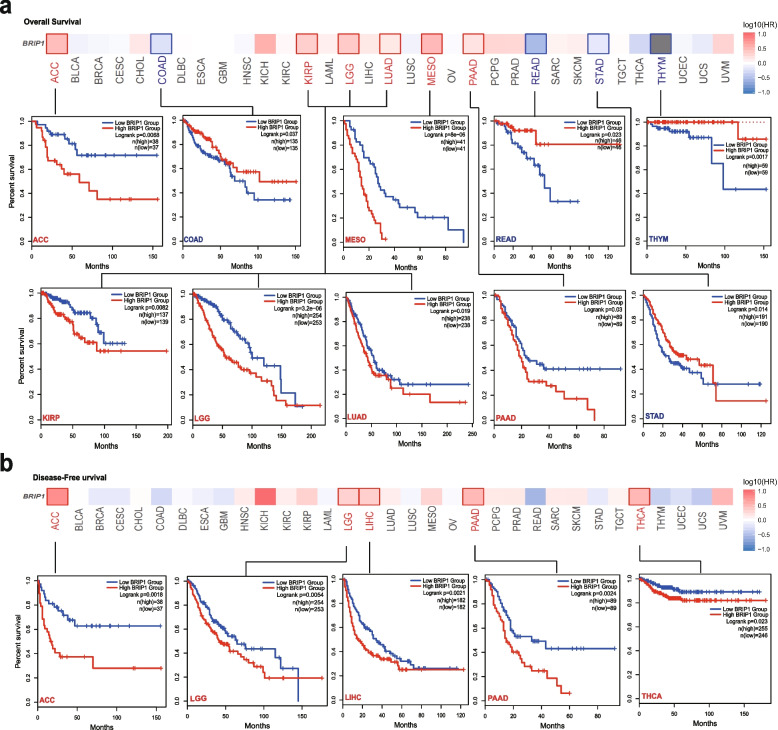
Correlation between BRIP1 gene expression and survival prognosis of cancers in TCGA. We used the GEPIA2 tool to perform overall survival (**a**) and disease-free survival (**b**) analyses of different tumors in TCGA by BRIP1 gene expression. The survival map and Kaplan-Meier curves with positive results are given

According to the expressional levels of BRIP1, we divided the cases into two groups (low and high expression groups) to explore the correlation between gene expression and the survival status of patients across different tumors. As performed in Fig. 2a, high expression of BRIP1 was associated with worse OS (overall survival) prognosis for ACC, KIRP, LGG, LUAD, MESO (mesothelioma), and PAAD (*P*<0.05 for all). In contrast, high BRIP1 expression was linked to better OS prognosis of COAD, READ, STAD, and THYM (*P*<0.05 for all). Data of disease-free survival (DFS) analysis indicated that high expression of BRIP1 was correlated to poor DFS prognosis of ACC, LGG, LIHC, PAAD (pancreatic adenocarcinoma), and THCA (*P*<0.05 for all) (Fig. 2b).

Besides, evidence from the Kaplan-Meier plotter tool showed that high BRIP1 expression was associated with poor OS (*P*=0.026), PPS (post-progression survival) (*P*<0.01), and DMFS (distant metastasis-free survival) (*P*<0.001) prognosis for breast cancer (Figure S10a). For ovarian cancer, high expression of BRIP1 was correlated to worse OS (*P*=0.027) and PFS (progress-free survival) (*P*=0.036) in patients (Figure S10b). Similarly, high BRIP1 expression was linked to the poor OS, FP (first progression), and PPS (*P*<0.001 for all) prognosis for lung cancer (Figure S10c). Conversely, high expression of BRIP1 was correlated to better OS (*P*=0.024), FP (*P*<0.01), and PPS (*P*<0.001) prognosis for gastric cancer (Figure S10d). In addition, a positive correlation was found between high BRIP1 expression and worse OS (*P*=0.021), PFS (*P*<0.01), RFS (relapse-free survival) (*P*=0.047), and FP (*P*<0.001) prognosis for liver cancer (Figure S10e).

We also used the Sangerbox tool to evaluate the independent prognostic role of BRIP1 across all TCGA tumors. As shown in Figure S11, BRIP1 could serve as an independent prognostic biomarker to predict the OS of patients for PCPG, ACC, KICH, LGG, READ, MESO, LIHC, KIRP, PAAD, UCEC, PRAD (prostate adenocarcinoma), and LUAD; to predict the DSS (disease-specific survival) of patients for PCPG, ACC, KICH, LGG, KIRC, COAD, MESO, LIHC, KIRP, PAAD, PRAD, and LUAD; to predict the DFI (disease-free interval) of patients for THCA, LIHC, KIRP, and PAAD; and to predict the PFI (progress-free interval) of patients for UVM (uveal melanoma), PCPG, ACC, KICH, LGG, THCA, MESO, LIHC, KIRP, PAAD, PRAD, and LUAD (*P*<0.05 for all). Survival analysis data of Sangerbox showed that the AUC (area under the curve) for 1 year, 3 years, and 5 years was moderate to high in predicting the OS of patients for ACC, COAD, KIRP, LGG, LUAD, MESO, PAAD, READ, STAD, and THYM (Figure S12).

Moreover, we utilized the univariate and multivariate Cox regression analysis to calculate the prognostic factors of OS for KIRP, ACC, LGG, COAD, READ, STAD, THYM, LUAD, MESO, and PAAD (Table S2). Clinical characteristics and BRIP1 expression were calculated. In addition, we combined the parameters with statistical significance from univariate analysis to construct the prognostic nomograms for predicting the 1 year, 3 year, and 5 year survival probability for the above cancers (Figure S13). Therefore, the high expression of BRIP1 might significantly correlate to the worse prognosis of many tumors.

### Enrichment analysis of BRIP1

**Fig. 3:**
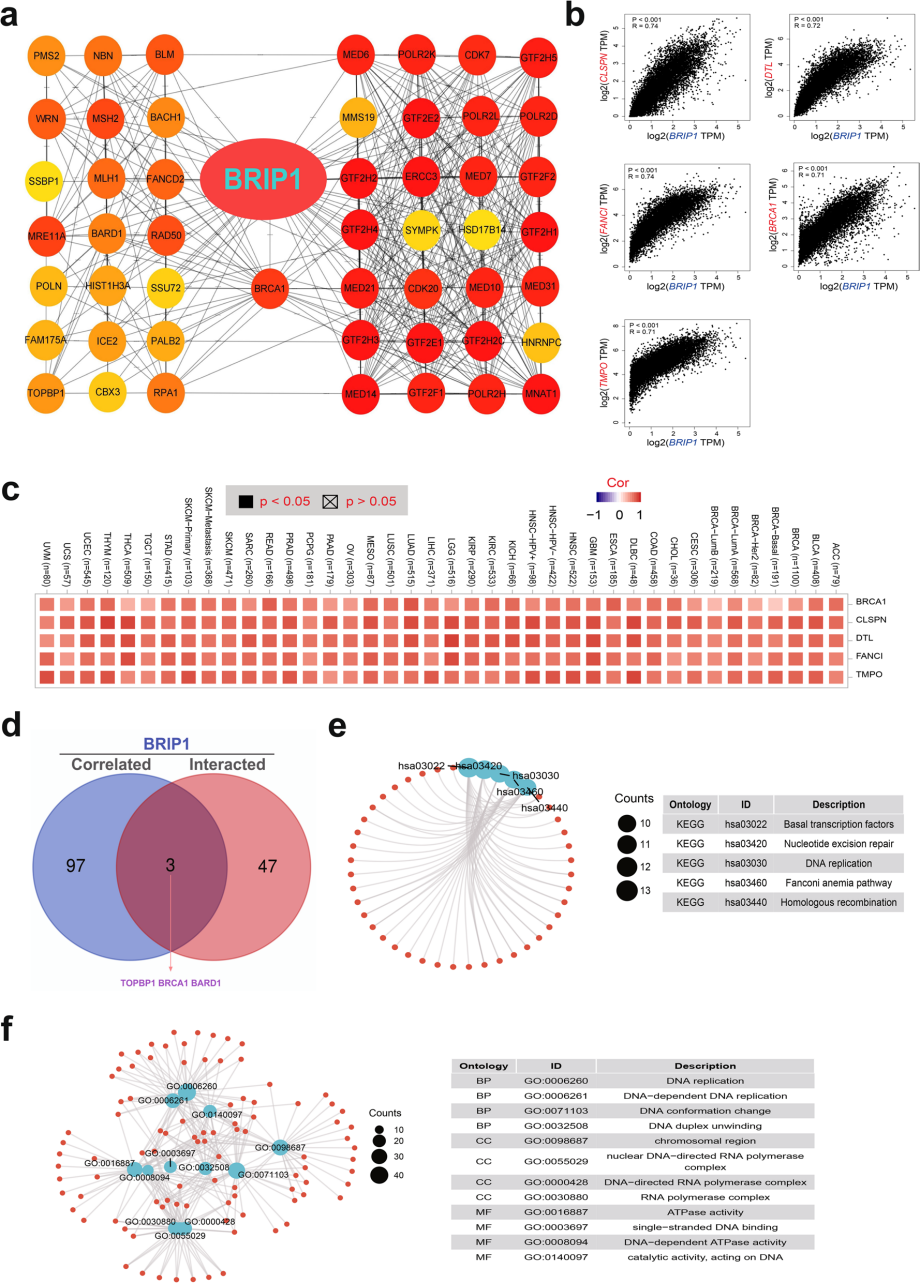
BRIP1-related gene enrichment analysis. **a** We first obtained the available experimentally determined BRIP1-binding proteins using the STRING database. **b** Using the GEPIA2 tool, we also obtained the top 100 BRIP1-correlated genes in TCGA projects and analyzed the expression correlation between BRIP1 and the top five genes with the highest correlation, including CLSPN, PLOD3, CALU, GCC1, and MYBBP1A. **c** The corresponding heatmap data in the detailed cancer types are displayed. **d** An intersection analysis of the BRIP1-binding and correlated genes was conducted. **e**, **f** KEGG and GO enrichment analyses predicted the functional roles of target genes based on four aspects, including KEGG pathway analysis, biological process, cellular components, and molecular functions. The count represents the number of genes in a GO/KEGG term

Based on the online tool of String and GEPIA2, 50 targeting BRIP1-binding proteins and 100 BRIP1-correlated genes were selected to further explore the multimolecular characteristics and role of BRIP1 in the carcinogenesis, progress, and prognosis of various tumors. Figure 3a shows the PPI network of the 50 proteins. The top 100 most correlated genes of BRIP1 were identified using GEPIA2. As shown in Fig. 3b, BRIP1 was mostly correlated to CLSPN, FANCI, DTL, BRCA1, and TMPO, with correlation coefficients of 0.74, 0.74, 0.72, 0.71, and 0.71, respectively (*P*<0.001 for all). In addition, a positive correlation was noticed in numerous cancers between the BRIP1-correlated five genes and BRIP1 (Fig. 3c). Combining the two sets of genes, we found three common members, TOPB1, BRCA1, and BARD1 (Fig. 3d).

Enrichment analyses of KEGG and GO were conducted among the combination of the two sets of genes. As shown in Fig. 3e, pathways of “basal transcription factors,” “homologous recombination,” and “nucleotide excision repair” might partially explain the role of BRIP1 on tumorigenesis of different cancers. Results from GO enrichment analysis further verified that most BRIP1-related genes are associated with DNA metabolism cellular biology or pathways (e.g., DNA replication, DNA conformation change, chromosomal region, nuclear DNA-directed RNA polymerase complex, ATPase activity, single-stranded DNA binding) (Table S3 and Fig. 3e).

### Mutation, methylation, and genome-wide association of BRIP1 analysis

**Fig. 4:**
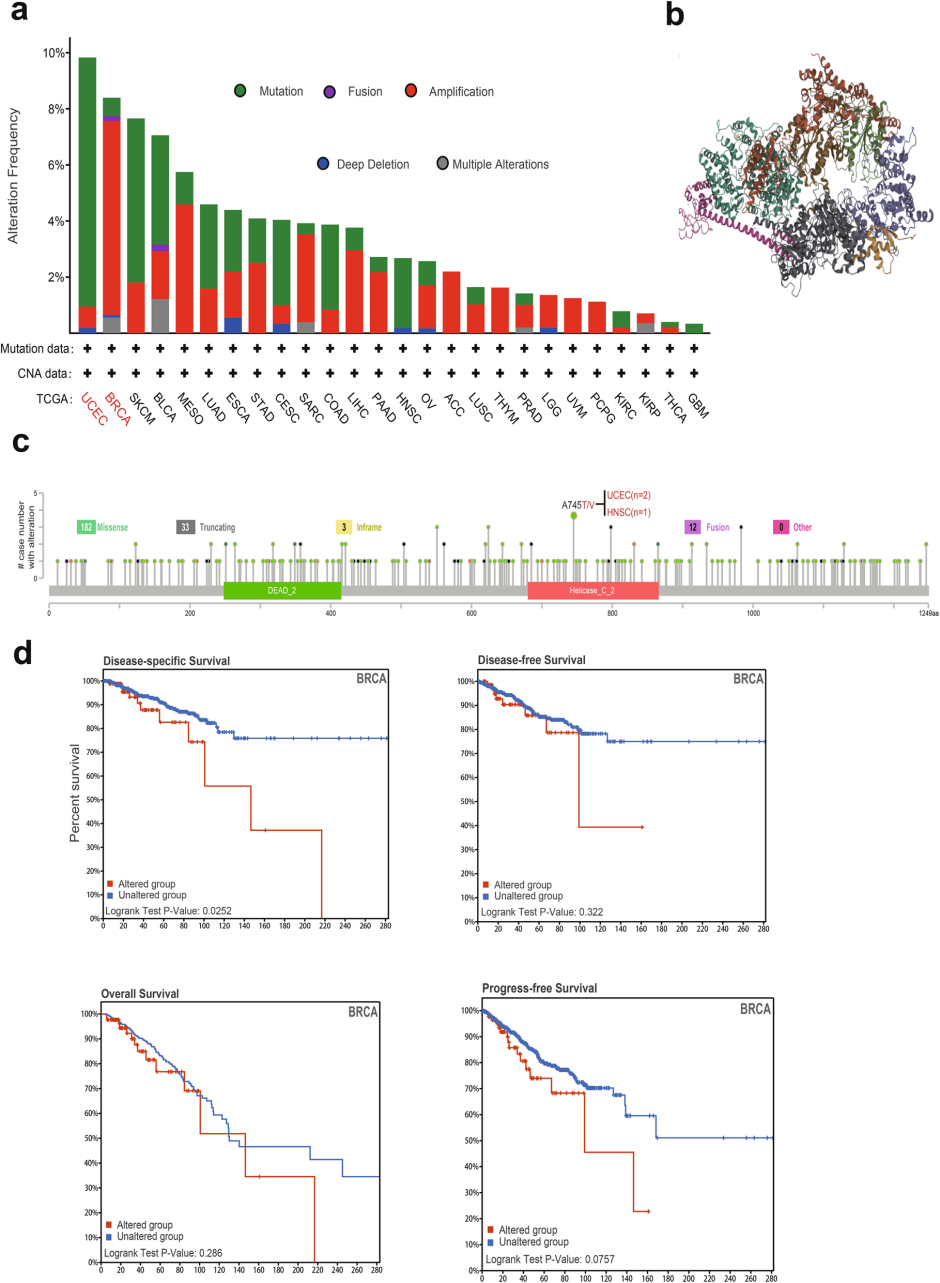
Mutation feature of BRIP1 in different TCGA tumors. We analyzed the mutation features of BRIP1 for the TCGA tumors using the cBioPortal tool. The alteration frequency with mutation type (**a**) and mutation site (**c**) are displayed. **b** 3D structure of BRIP1. **d** The potential correlation between mutation status and disease-specific, progression-free, overall, and disease-free survival of BRCA

We utilized multiple online databases to obtain the genetic alteration information of BRIP1 across different tumors. Patients with UCEC presented with the highest alteration frequency of BRIP1 (near 10%), and the primary type for BRCA patients is the “amplification” of CNA, with an alteration frequency of 8% (Fig. 4a). The 3D structure of BRIP1 was performed in Fig. 4b. Figure 4c shows the case number, sites, and types of BRIP1 alteration, and the primary genetic alteration type of “missense” mutation was observed (182 cases). Besides, A745T/V alteration in the Helicase_C_2 domain was found in two cases of UCEC and one case of HNSC. Moreover, the correlation between BRIP1 alteration and clinical prognosis for BRCA was investigated. As shown in Fig. 4d, the alteration of BRIP1 was associated with poor prognosis in DSS (*P*=0.0252) but not in PFS, OS, and DFS (*P*>0.05 for all).

**Fig. 5:**
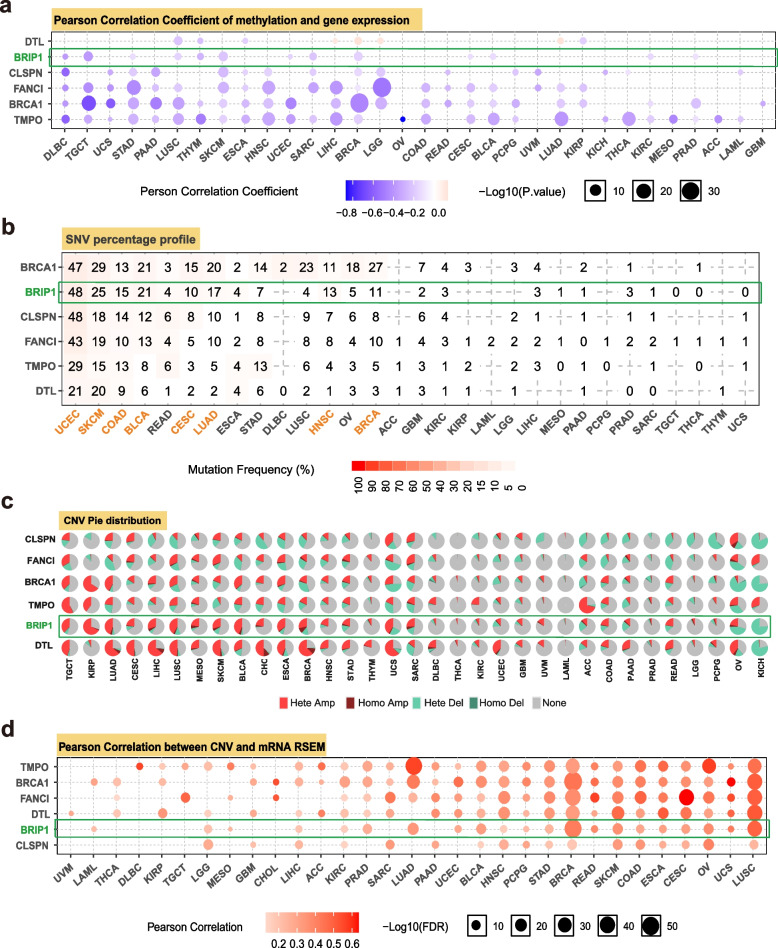
Regulatory mechanism of BRIP1 and the correlated genes expression and its role in cancer. **a** The correlation between DNA methylation and the expression levels of BRIP1 and the correlated genes in different cancers. **b** SNV frequency of BRIP1 and the correlated genes in different cancers. The deeper of color, the higher of mutate frequency. **c** Global profile that shows the constitute of heterozygous/homozygous CNV of BRIP1 and the correlated genes in each cancer. Hete Amp, heterozygous amplification; Hete Del, heterozygous deletion. Homo Amp, homozygous amplification; Homo Del, homozygous deletion; None, no CNV. **d** The association between paired mRNA expression and CNV percent samples, based on Pearson’s product moment correlation coefficient

Using the database of MEXPRESS, the potential correlation between BRIP1 expression and DNA methylation levels was explored. As shown in Table S4, a significant correlation was observed between BRIP1 expression and methylation for 24 TCGA tumors, including BLCA, BRCA, CESC, COAD, DLBC, GBM, HNSC, LAML (acute myeloid leukemia), LGG, LIHC, LUAD, LUSC, OV, PCPG, PRAD, READ, SARC, SKCM, STAD, TGCT, THCA, THYM, UCEC, and UCS (*P*<0.05 for all). The correlation between methylation and BRIP1 expression among different cancers was also evaluated by the GSCALite database. Generally, negative correlations between gene expression and methylation across cancers were observed (Fig. 5a).

In addition, the mutation frequency of SNV across TCGA tumors was also analyzed on the GSCALite tool. Figure 5b shows a high SNV in many cancers, including UCEC, SKCM, COAD, BLCA, CESC, LUAD, HNSC, and BRCA. The constitute of heterozygous/homozygous CNV of BRIP1 in 33 TCGA tumors was presented in Fig. 5c. It is worth noticing that heterozygous amplification is the primary type of CNV in KIRP, while the homozygous deletion type of CNV is the primary type in KICH. Besides, the correlation between CNV and mRNA expression of BRIP1 was also explored, and the results are shown in Fig. 5d. Notably, the highest correlation was observed for BRCA and LUSC, which indicated that CNV significantly regulates the BRIP1 expression in BRCA and LUSC.

**Fig. 6:**
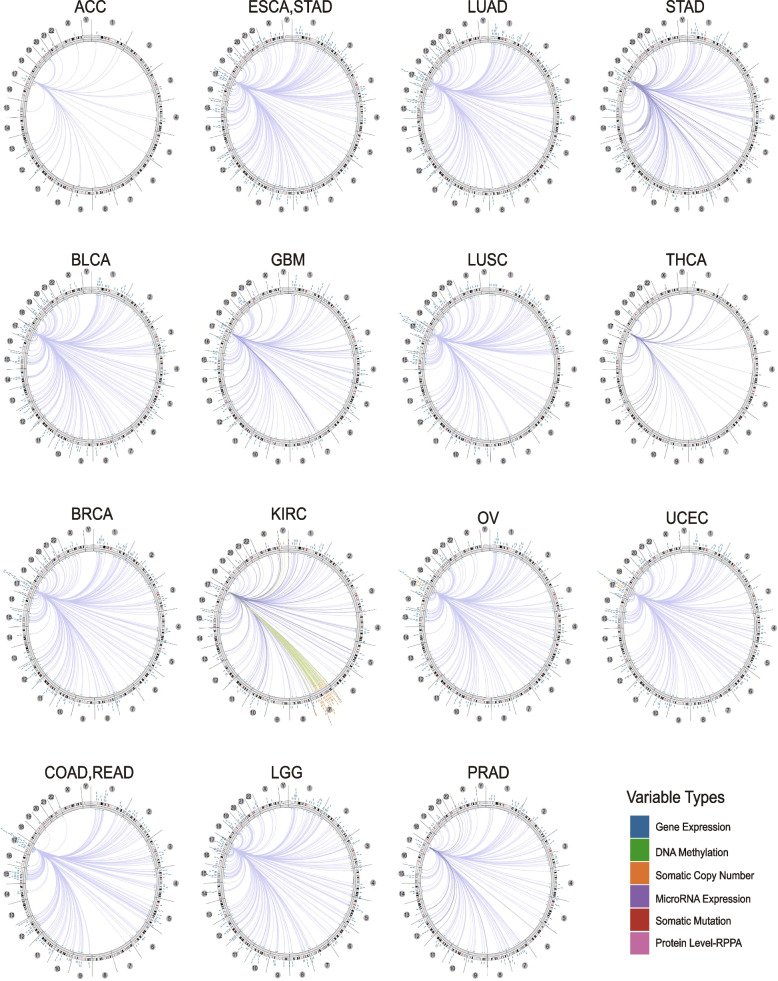
The correlation between BRIP1 and other genes from the TCGA database (Regulome program). Note: The circular layout displays the associations as edges in the center connecting the features (with genomic coordinates) displayed around the perimeter. The outer ring displays cytogenetic bands. The inner ring displays associations that contain features lacking genomic coordinates

Besides, we further explored the genome-wide association of BRIP1 mRNA in cancer by analyzing the correlation between BRIP1 and other genes using the Regulome Explorer. As shown in Fig. 6, a positive correlation was observed in multiple cancers, and the detailed information was presented in Table S5-S19. To further understand the multimolecular characteristics and role of BRIP1 in the tumorigenesis of cancers, we further analyzed the association between BRIP1 expression and inhibition or activation of ten major signaling pathways, the detailed information for calculating the pathway score was described at length elsewhere [29]. As shown in Figure S14, BRIP1 was highly correlated to the activation of apoptosis, cell cycle, DNA damage response, and inhibition of hormone ER and RAS/MAPK signaling pathways. Of note, we found extensive associations between the expression of BRIP1 and marker genes of RNA modification (Figure S15), suggesting that different types of RNA modifications may regulate the expression of BRIP1 across cancers.

### Immune analysis of BRIP1

**Fig. 7:**
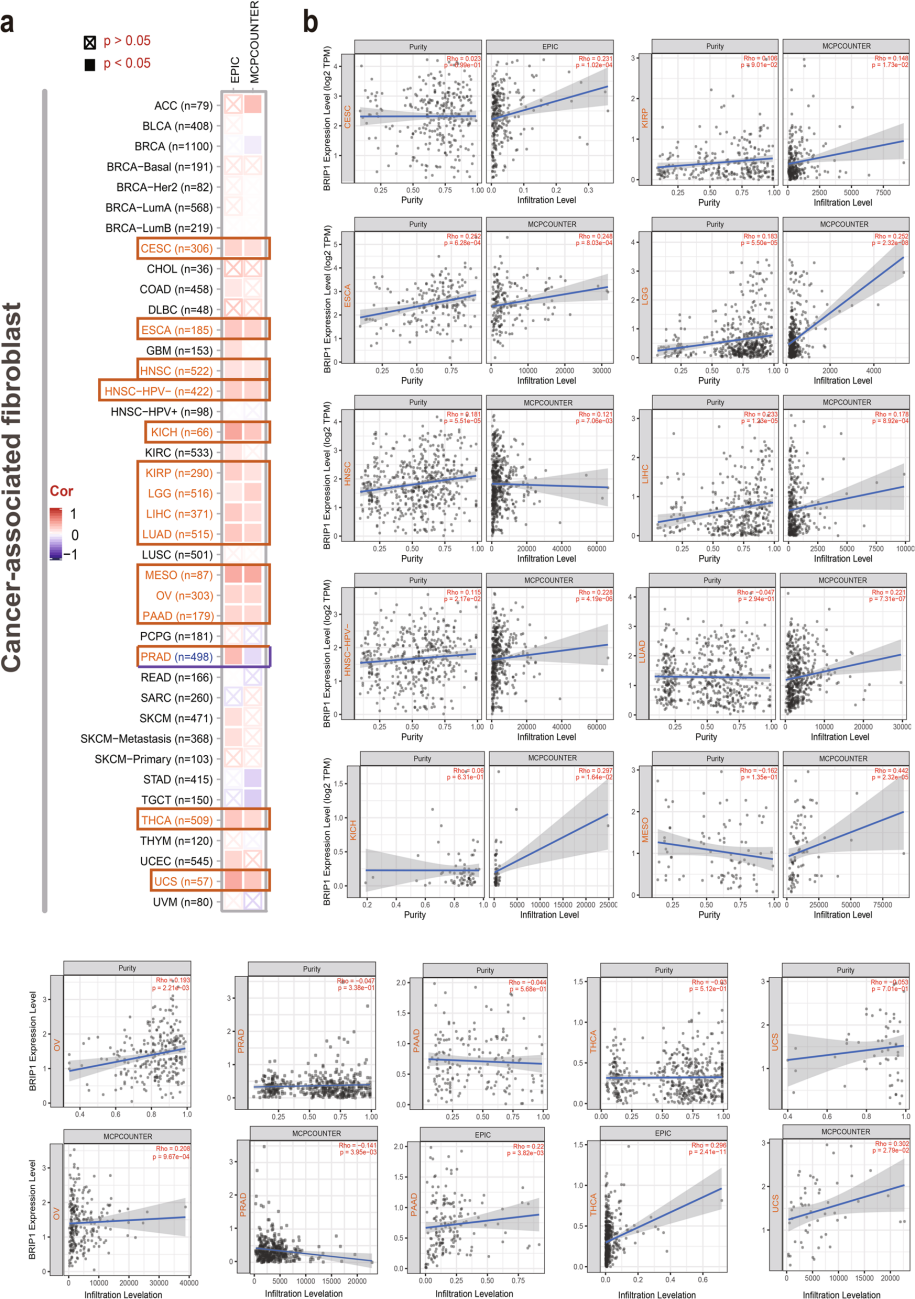
Correlation analysis between BRIP1 expression and immune infiltration of cancer-associated fibroblasts. Different algorithms were used to explore the potential correlation between BRIP1 expression levels and the infiltration levels of cancer-associated fibroblasts across all TCGA tumorss

As a prominent part of the tumor microenvironment, tumor-infiltrating immune cells complexly interact with cancer’s carcinogenesis, development, and prognosis [30–32]. As one of the most abundant stromal cells populated in the tumor microenvironment, cancer-associated fibroblasts (CAFs) are essentially involved in the progression of cancers [33, 34]. In the current study, we utilized multiple algorithms to explore the association between immune cell infiltration and BRIP1 expression across different cancers. Generally, we observed a series of positive correlations between the expression of BRIP1 and the CAFs estimated infiltration levels in CESC, ESCA, HNSC, HNSC-HPV-, KICH, KIRP, LGG, LIHC, LUAD, MESO, OV, PAAD, PRAD, THCA, and UCS (Fig. 7a). Additionally, a significantly positive correlation was noticed between the BRIP1 expression and the infiltrating CD8^+^ T cells in HNSC-HPV+, KIRC, LUAD, and THYM based on most algorithms (Figure S16a). Figure 7b and Figure S16b showed the scatter plots of the above cancers. As an example, BRIP1 expression in CESC is positively associated with the infiltration level of CAFs (Rho=0.231, *P*=1.02e−04) based on the EPIC algorithm (Fig. 7b). Besides, the relationship between BRIP1 expression and MSI (microsatellite instability)/TMB (tumor mutational burden)/neoantigen in all TCGA cancers was investigated. We observed positive correlations between BRIP1 expression and MSI in GBM, LUSC, UCEC, COAD, STAD, KIRC, READ, and KICH, but we also noticed a negative correlation in DLBC (*P*<0.05 for all) (Figure S17). BRIP1 expression is positively correlated to TMB in ACC, LUAD, PRAD, UCEC, COAD, STAD, SKCM, KIRC, and KICH, but negatively correlated to TMB in KIRP (Figure S18, *P*<0.05 for all). Besides, a positive correlation was only found between neoantigen and BRIP1 expression in PRAD, LUAD, BRCA, UCEC, and STAD (Figure S19, *P*<0.05 for all). Additionally, a statistically significant correlation between BRIP1 expression and immune checkpoints and pathways across most TCGA tumors was observed, and the heatmaps were presented in Figure S20. The above findings are worthy of further in-depth research to explore their clinical value.

## Discussion

The aberrant expression of BRIP1 is associated with many human diseases, especially cancers [13, 18, 19, 35]. However, the role of BRIP1 in the carcinogenesis of various cancers remains to be illuminated. Pan-cancer studies have recently emerged as a novel perspective for understanding the molecular mechanisms of the occurrence and the development of human cancers [36–42], and we failed to obtain the pan-cancer analysis of BRIP1 across human tumors from the literature search. Therefore, we used the multilevel data of human databases to analyze the expression, mutation, methylation, activation or inhabitation of cancer-related pathways, and immune characteristics of the BRIP1 gene in 33 TCGA tumors. Our results indicated a prognosis-related differential expression of BRIP1 between various cancers and their corresponding normal tissues. The genetic alteration, CNV, and SNV of BRIP1 were observed in various cancers, and CNV could regulate BRIP1 expression in several cancer types. BRIP1 methylation is negatively correlated to its mRNA expression in many human tumors, and the expression was associated with the activation and inhabitation of several cancer-relevant pathways. BRIP1 expression was also associated with the immune infiltration levels of CAFs and CD8^+^ T cells in specific cancers.

The phylogenetic tree analysis of BRIP1 in the current study suggests a conservative characteristic of BRIP1 protein structure in various species, indicating there might be similar mechanisms of BRIP1 under physiological state. Previous studies suggested that BRIP1 has an anti-oncogenic effect and is deregulated in many cancers [13, 43, 44]. Controversially, data from ONCOMINE, TIMER2, and HPA databases suggested that BRIP1 is highly expressed in most cancers, and results from GEPIA2 suggested that gene expression is associated with cancer stages, indicating that BRIP1 plays an oncogene role in human tumors. To further figure out the role of BRIP1 in tumors, we used various databases to explore the prognostic potential of BRIP1 in various cancers according to gene expression. Our analyses showed that high BRIP1 expression was associated with worse prognosis of PCPG, ACC, KICH, LGG, MESO, LIHC, KIRP, PAAD, UCEC, BRCA, OV, PRAD, and LUAD and was also correlated to better prognosis of COAD, READ, STAD, and THYM. We then performed a Cox regression analysis to explore the role of BRIP1 expression and several clinical characteristics in the survival of different cancers, and the results verified that BRIP1 acted as a risk factor in KIRP, ACC, LGG, MESO, and PAAD patients, and played a protective role in COAD, READ, STAD, and THYM patients. The survival data from various databases were not completely consistent, which might be due to the heterogeneity of data collection in different databases [38]. Moreover, some other factors may contribute to the diverse prognostic role of BRIP1 in cancers. Firstly, high mutation frequency with various mutation types was noticed in many cancers. The different BRIP1 variation frequency and types across cancers might contribute to their diverse regulation mechanisms in the development and survival of cancers. Secondly, extensive correlations were found between the expression of BRIP1 and marker genes of RNA modification. The regulation of RNA modification across cancers could also be responsible for the inconsistent effect of BRIP1 in cancers. Thirdly, it is well known that the high infiltration of CD8+ T cells is associated with better survival of tumors [45]. In the current study, we found that the high BRIP1 expression was related to high CD8+ T cell infiltration in COAD, STAD, and THYM, and low infiltration in KIRP and LGG. Besides, the opposite correlation between BRIP1 expression and MSI and TMB among various cancers may also result in different clinical outcomes. Lastly, as a redundant protein, the significance of the BRIP1 mutation in cancers remains to be further clarified [46]. In summary, BRIP1 has the potential to be the diagnostic/prognostic biomarker in many tumors and may act as an effective target for personalized treatment, which deserves further experiments to verify its clinical value.

In addition, the association between BRIP1 mutations and the development of breast, ovarian, and cervical cancers has been reported [18, 19, 47, 48]. In the current study, we also observed a high genetic alteration frequency in BRCA, and extensive SNVs of BRIP1 were found in several cancer types. Adhered to the previous report that the increase of copy number was always followed by the increase of gene expression [49], we also found that the increase of BRIP1 CNV could, at least in part, result in the upregulation of BRIP1 expression, especially in BRCA and LUSC. Besides, as one of the significant forms of epigenetic modification, DNA methylation is also strongly involved in regulating gene expression [50]. The current study noticed statistical significance between DNA methylation and gene expression across multiple cancers, suggesting that DNA methylation could also be one of the factors that regulate BRIP1 expression in cancers. Moreover, the correlation analysis between BRIP1 expression and ten famous cancer-related pathways among 32 cancer types suggested that BRIP1 was highly correlated to the activation of apoptosis, cell cycle, DNA damage response, and inhibition of hormone ER and RNS/MARK signaling pathways. Therefore, we hypothesize that the genetic changes were crucially evolved in regulating BRIP1 expression, and the mechanisms under the regulatory rules on BRIP1 expression across different tumors are worthy of further studies and exploration.

It is well known that the tumor cells are supervised by immune cells, and the immunity deficiency could contribute to tumorigenesis, progression, and worse clinical outcomes in cancer patients [38]. In the current work, we found a significantly positive correlation between BRIP1 expression and immune cell infiltration of CAFs and CD8^+^ T cells in many cancers. Positive correlations between MSI/TBM, and BRIP1 expression were also observed in COAD, STAD, KIRC, and KICH. Of interest, positive correlations between TMB/neoantigen and BRIP1 expression were observed in LUAD, PRAD, and STAD, suggesting BRIP1 could be an immunotherapy target in these cancers. Besides, we observed positive correlations between BRIP1 expression and the immune pathways of “activated CD4 T cell,” “memory B cell,” and “type 2 T helper cell,” and immune checkpoints in many cancers, which would help us to further understand the role of BRIP1 in specific tumor’s immune microenvironment.

As the first study to explore the multimolecular characteristics and role of BRIP1 in human tumors, several limitations need to be recognized. First, since the current study was conducted among multiple online databases, data heterogeneity would inevitably exist, resulting in the inconsistency of results. Moreover, based on the fact that all the results are observational studies, and no causal conclusion could be drawn; therefore, any over-interpretation of the data should be avoided. Second, although our work suggested that BRIP1 has the potential to be a diagnostic/prognostic biomarker for several certain cancers and could act as an effective target for immunotherapy in some tumors, further experiments in vitro/in vivo are required to verify these hypotheses.

## Conclusion

Our concentrative and systematic study of BRIP1 in pan-cancer suggested that there are significant associations between BRIP1 expression, genetic alterations, DNA methylation, activation or inhabitation of cancer-related pathways, immune cell infiltration, MSI/TMB/neoantigen, immune pathways, and immune checkpoints across multiple human cancers, which would provide a novel insight in understanding the genetic landscape and biologic function of BRIP1 in cancers.

## Supplementary Information

**Additional file 1: Figure S1.** Role of BRIP1 in Fanconi anemia pathway.

**Additional file 2: Figure S2.** Study design of the current study. CNA: Copy number alteration; SNV: Single nucleotide variation; CNV: Copy number variation. PPI: Protein-protein interaction; KEGG: Kyoto Encyclopedia of Genes and Genomes; GO: Gene Oncology; MF: molecular functions; CC: cellular components; BP: biological processes; MSI: Microsatellite instability; TMB: Tumor mutational burden.

**Additional file 3: Figure S3.** Structural characteristics of BRIP1 in various species. (a) Genomic location of human BRIP1; (b) Conserved domains of BRIP1 protein among different species.

**Additional file 4: Figure S4.** Phylogenetic tree of BRIP1. We used a constraint-based multiple alignment online tool of NCBI to get the phylogenetic tree of BRIP1 in different species.

**Additional file 5: Figure S5.** The expression levels of BRIP1 in different tumors and in different tissues, blood cells, and brain tissues under the normal physiological state. We analyzed the expression of the BRIP1 gene in different tumors using antibodies of HPA005474 (a), using the consensus datasets of HPA, GTEx, and FANTOM5 to explore the expression of BRIP1 in different tissues (b) and brain tissues (d), using the consensus datasets of HPA, Monaco, and Schmieder to explore the expression of BRIP1 in different blood cells (c).

**Additional file 6: Figure S6.** The expression levels of BRIP1 in various cancers and pathological stages. (a) The expression levels of BRIP1 in ACC, HNSC, LAML, LGG, OV, SKCM, and TGCT in the TCGA project were compared with the corresponding normal tissues of the GTEx databases. The expression levels of BRIP1 by different pathological stages of BLCA, CESC, CHOL, COAD, UCEC (b); DLBC, ESCA, HNSC, LUAD (c); and PAAD, READ, STAD, TGCT (d).

**Additional file 7: Figure S7.** Pooled analysis on the differential BRIP1 expression between normal and tumor tissues via the ONCOMINE database. (a) Breast cancer; (b) Sarcoma; (c) colorectal cancer; (d) Head & neck cancer.

**Additional file 8: Figure S8.** Differential expression analysis of BRIP1 in various subtypes across different cancers via TISIDB. Cancer types with statistical significance were shown. (a) The expression of BRIP1 in different molecular subtypes and (b) immune subtypes of cancers.

**Additional file 9: Figure S9.** Differential expression analysis of BRIP1 in various subtypes across different cancers via TISIDB. Cancer types without statistical significance were shown. (a) The expression of BRIP1 in different molecular subtypes and (b) immune subtypes of cancers.

**Additional file 10: Figure S10.** Correlation between BRIP1 expression and prognosis of cancers using the Kaplan-Meier plotter. We used the Kaplan-Meier plotter to assess the survival status of patients via the expression levels of the BRIP1 gene in breast cancer (a), ovarian cancer (b), lung cancer (c), gastric cancer (d), and liver cancer (e) cases, including OS, PPS, DMFS, PFS, FP, and RFS.

**Additional file 11: Figure S11.** Forest plots depicting the prognostic role of BRIP1 in various TCGA tumors. The hazard ratio of OS (a), DSS (b), DFI (c), and PFI (d) for patients with differential BRIP1 expression across TCGA tumors.

**Additional file 12: Figure S12.** Correlation between BRIP1 expression and survival prognosis of cancers in TCGA. We used the GEPIA2 tool to perform overall survival analyses of different tumors in TCGA by BRIP1 gene expression. The cancer types with positive results were highlighted. We used Sangerbox online tool to obtain the ROC curves of OS for the positive cancer types from GEPIA2 (THYM was not available).

**Additional file 13: Figure S13.** Regression-based prognostic nomograms for different TCGA tumors. Clinical characteristics which were significantly associated with OS and BRIP1 expression were used to construct the prognostic nomograms.

**Additional file 14: Figure S14.** The proportion of cancer types with BRIP1 and its most correlated five genes are significantly associated with activation (red) or inhibition (blue) of the ten key signaling pathways in 31 cancer types.

**Additional file 15: Figure S15.** Correlation analysis between the expression of BRIP1 and marker genes of RNA modification.

**Additional file 16: Figure S16.** Correlation analysis between BRIP1 expression and immune infiltration of CD8+ T-cells. Different algorithms were used to explore the potential correlation between the expression levels of BRIP1 and the infiltration levels of CD8+ T-cells across all types of cancer in TCGA.

**Additional file 17: Figure S17.** Correlation between BRIP1 expression and microsatellite instability. Based on the different tumors of TCGA, we explored the potential correlation between BRIP1 expression and microsatellite instability (MSI). The *P*-value is supplied. The partial correlation (cor) values of +0.33 and -0.33 are marked.

**Additional file 18: Figure S18.** Correlation between BRIP1 expression and tumor mutational burden. Based on the different tumors of TCGA, we explored the potential correlation between BRIP1 expression and tumor mutational burden (TMB). The *P*-value is supplied. The partial correlation (cor) values of +0.72 and -0.72 are marked.

**Additional file 19: Figure S19.** Correlation between BRIP1 expression and neoantigen. Based on the different tumors of TCGA, we explored the potential correlation between BRIP1 expression and neoantigen. The *P*-value is supplied. The partial correlation (cor) values of +0.26 and -0.26 are marked.

**Additional file 20: Figure S20.** Correlation between BRIP1 expression, immune pathways (a), and immune checkpoints (b) across all TCGA tumors.

**Additional file 21: Table S1**. The significant changes of BRIP1 expression in transcription level in pan-cancers (ONCOMINE database). **Table S2.** Univariate and Multivariate COX regression for clinical characteristics and BRIP1 expression in various tumors. **Table S3.** Enrichment analysis of BRIP1-related genes across pancancer. **Table S4.** Association between BRIP1 DNA methylation and gene expression for the 24 tumors of TCGA. **Table S5.** The correlation between BRIP1 and other genes of ACC from the TCGA database. **Table S6.** The correlation between BRIP1 and other genes of BLCA from the TCGA database. **Table S7.** The correlation between BRIP1 and other genes of LUSC from the TCGA database. **Table S8.** The correlation between BRIP1 and other genes of OV from the TCGA database. **Table S9.** The correlation between BRIP1 and other genes of THCA from the TCGA database. **Table S10.** The correlation between BRIP1 and other genes of UCEC from the TCGA database. **Table S11.** The correlation between BRIP1 and other genes of BRCA from the TCGA database. **Table S12.** The correlation between BRIP1 and other genes of COAD, READ from the TCGA database. **Table S13.** The correlation between BRIP1 and other genes of ESCA with STAD from the TCGA database. **Table S14.** The correlation between BRIP1 and other genes of GBM from the TCGA database. **Table S15.** The correlation between BRIP1 and other genes of KIRC from the TCGA database. **Table S16.** The correlation between BRIP1 and other genes of LGG from the TCGA database. **Table S17.** The correlation between BRIP1 and other genes of LUAD from the TCGA database. **Table S18.** The correlation between BRIP1 and other genes of PRAD from the TCGA database. **Table S19.** The correlation between BRIP1 and other genes of STAD from the TCGA database.

## Data Availability

All the data in this study are available in the public databases listed in the “Materials and methods” section.

## Abbreviations

TME: Tumor microenvironment
BRIP1: BRCA1 interacting protein C-terminal helicase 1
FA: Fanconi anemia
SARC: Sarcoma
THYM: Thymoma
OS: Overall survival
DFS: Disease-free survival
PPI: Protein-protein interaction
KEGG: Kyoto Encyclopedia of Genes and Genomes
GO: Gene Oncology
MF: Molecular function
CC: Cellular components
BP: Biological processes
CAN: Copy number alteration
3D: Three-dimensional
DSS: Disease-specific survival
PFS: Progress-free survival
SNV: Single-nucleotide variation
CNV: Copy number variation
HPA: Human protein atlas
BLCA: Bladder urothelial carcinoma
KIRC: Kidney renal clear cell carcinoma
KIRP: Kidney renal papillary cell carcinoma
BRCA: Breast invasive carcinoma
HNSC: Head and neck squamous cell carcinoma
ESCA: Esophageal carcinoma
CHOL: Cholangiocarcinoma
COAD: Colon adenocarcinoma
GBM: Glioblastoma multiforme
LUAD: Lung adenocarcinoma
LGG: Brain lower grade glioma
UCEC: Uterine corpus endometrial carcinoma
LUSC: Lung squamous cell carcinoma
STAD: Stomach adenocarcinoma
TGCT: Testicular germ cell tumors
CESC: Cervical squamous cell carcinoma
READ: Rectum adenocarcinoma
UCS: Uterine carcinosarcoma
SARC: Sarcoma
THYM: Thymoma
DLBC: Lymphoid neoplasm diffuse large B cell lymphoma
ACC: Adrenocortical carcinoma
SKCM: Skin cutaneous melanoma
LIHC: Liver hepatocellular carcinoma
THCA: Thyroid carcinoma
KICH: Kidney chromophobe
OV: Ovarian serous cystadenocarcinoma
PCPG: Pheochromocytoma and paraganglioma
MESO: Mesothelioma
PAAD: Pancreatic adenocarcinoma
DMFS: Distant metastasis-free survival
PPS: Post-progression survival
FP: First progression
RFS: Relapse-free survival
PRAD: Prostate adenocarcinoma
DSS: Disease-specific survival
DFI: Disease-free interval
PFI: Progress-free interval
UVM: Uveal melanoma
AUC: Area under curve
LAML: Acute myeloid leukemia
CAFs: Cancer-associated fibroblasts
MSI: Microsatellite instability
TMB: Tumor mutational burden
