# Supplementary figures and images for: Multimolecular characteristics and role of BRCA1 interacting protein C-terminal helicase 1 (BRIP1) in human tumors: a pan-cancer analysis
Source: World J Surg Oncol. 2023 Mar 13;21:91. doi: 10.1186/s12957-022-02877-8 (PMC10010046; doi:10.1186/s12957-022-02877-8)

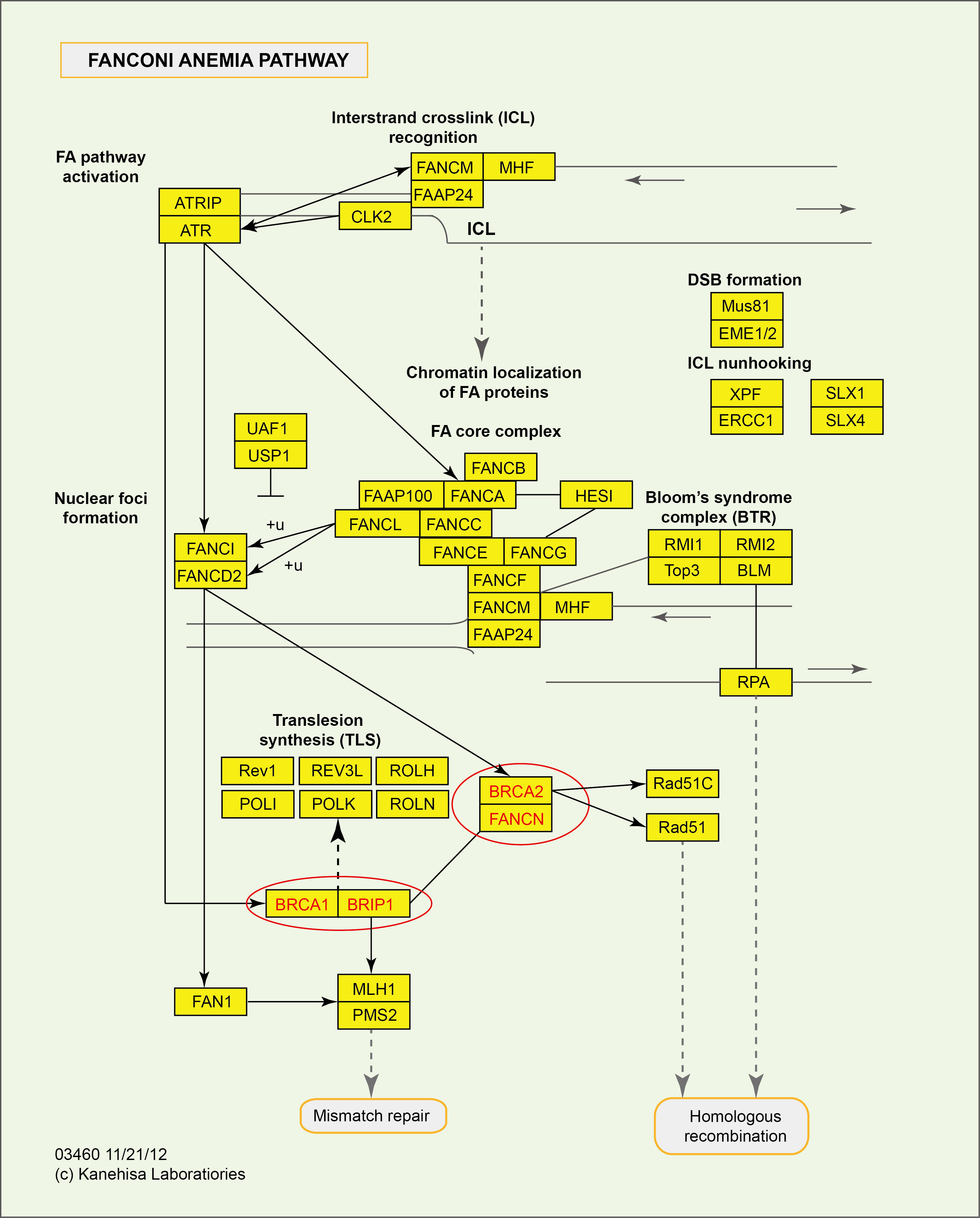

Supplement: Supplementary file 1 — Additional file 1: Figure S1. Role of BRIP1 in Fanconi anemia pathway. [file 12957_2022_2877_MOESM1_ESM.tiff]

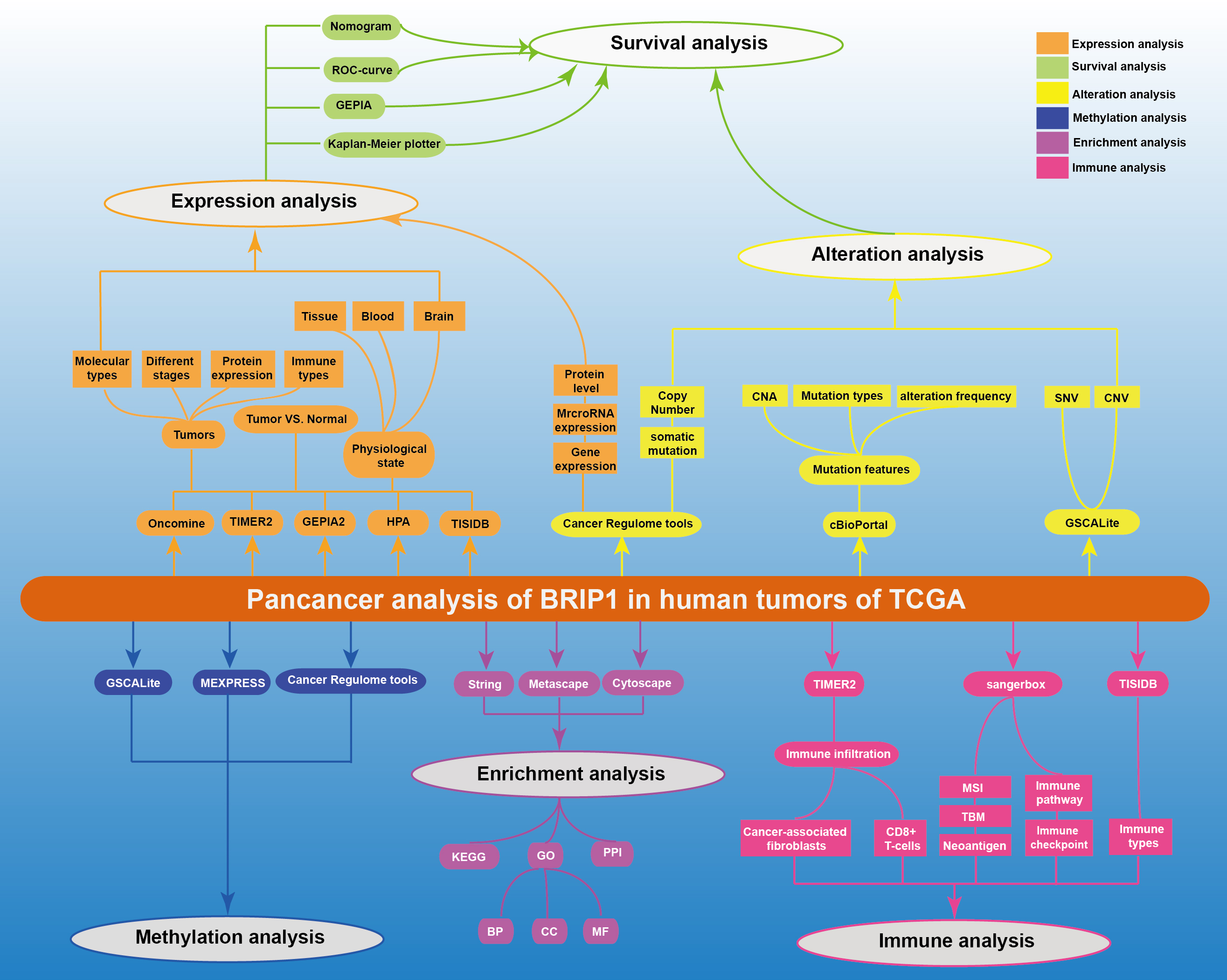

Supplement: Supplementary file 2 — Additional file 2: Figure S2. Study design of the current study. CNA: Copy number alteration; SNV: Single nucleotide variation; CNV: Copy number variation. PPI: Protein-protein interaction; KEGG: Kyoto Encyclopedia of Genes and Genomes; GO: Gene Oncology; MF: molecular functions; CC: cellular components; BP: biological processes; MSI: Microsatellite instability; TMB: Tumor mutational burden. [file 12957_2022_2877_MOESM2_ESM.tiff]

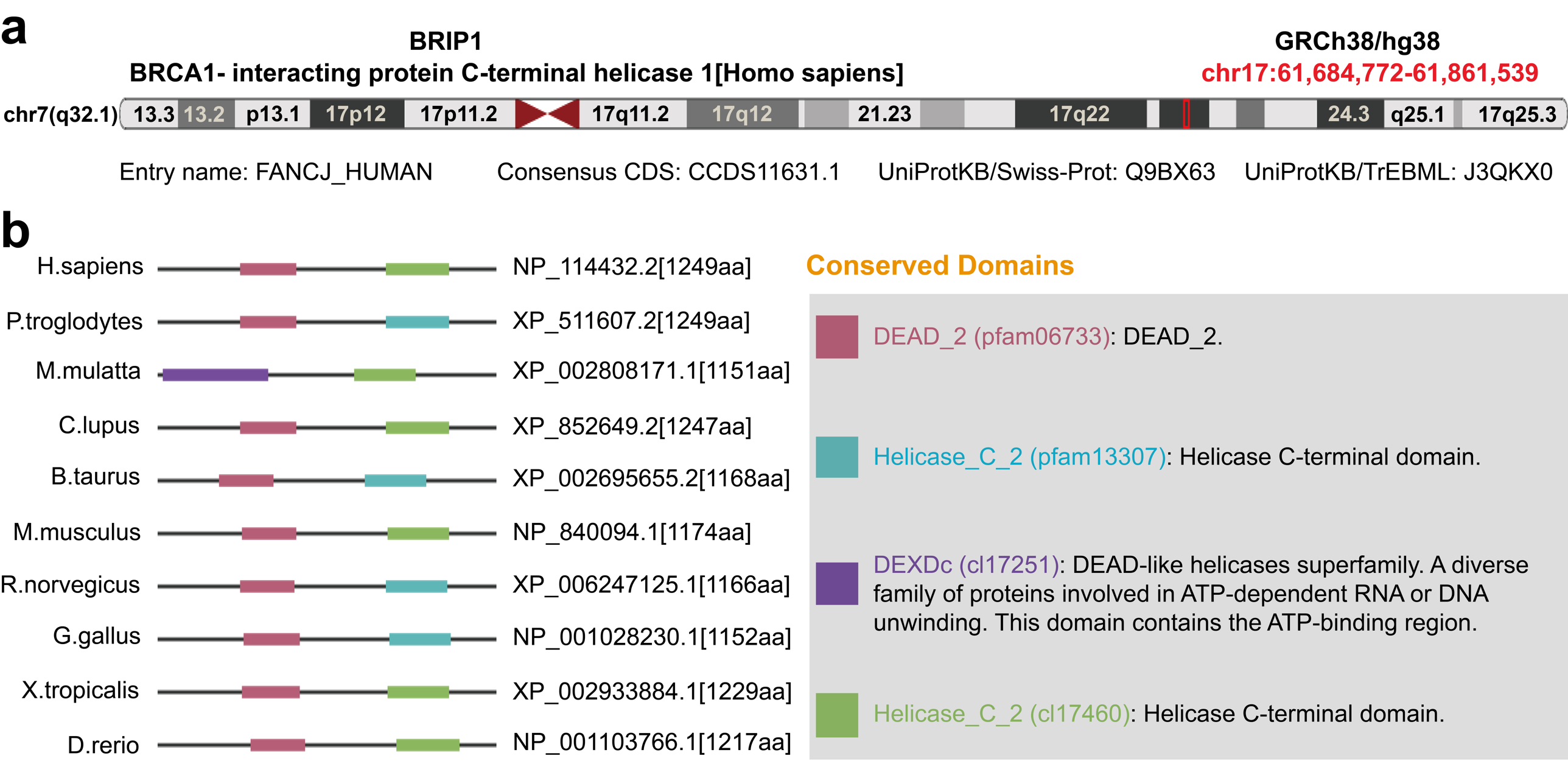

Supplement: Supplementary file 3 — Additional file 3: Figure S3. Structural characteristics of BRIP1 in various species. (a) Genomic location of human BRIP1; (b) Conserved domains of BRIP1 protein among different species. [file 12957_2022_2877_MOESM3_ESM.tiff]

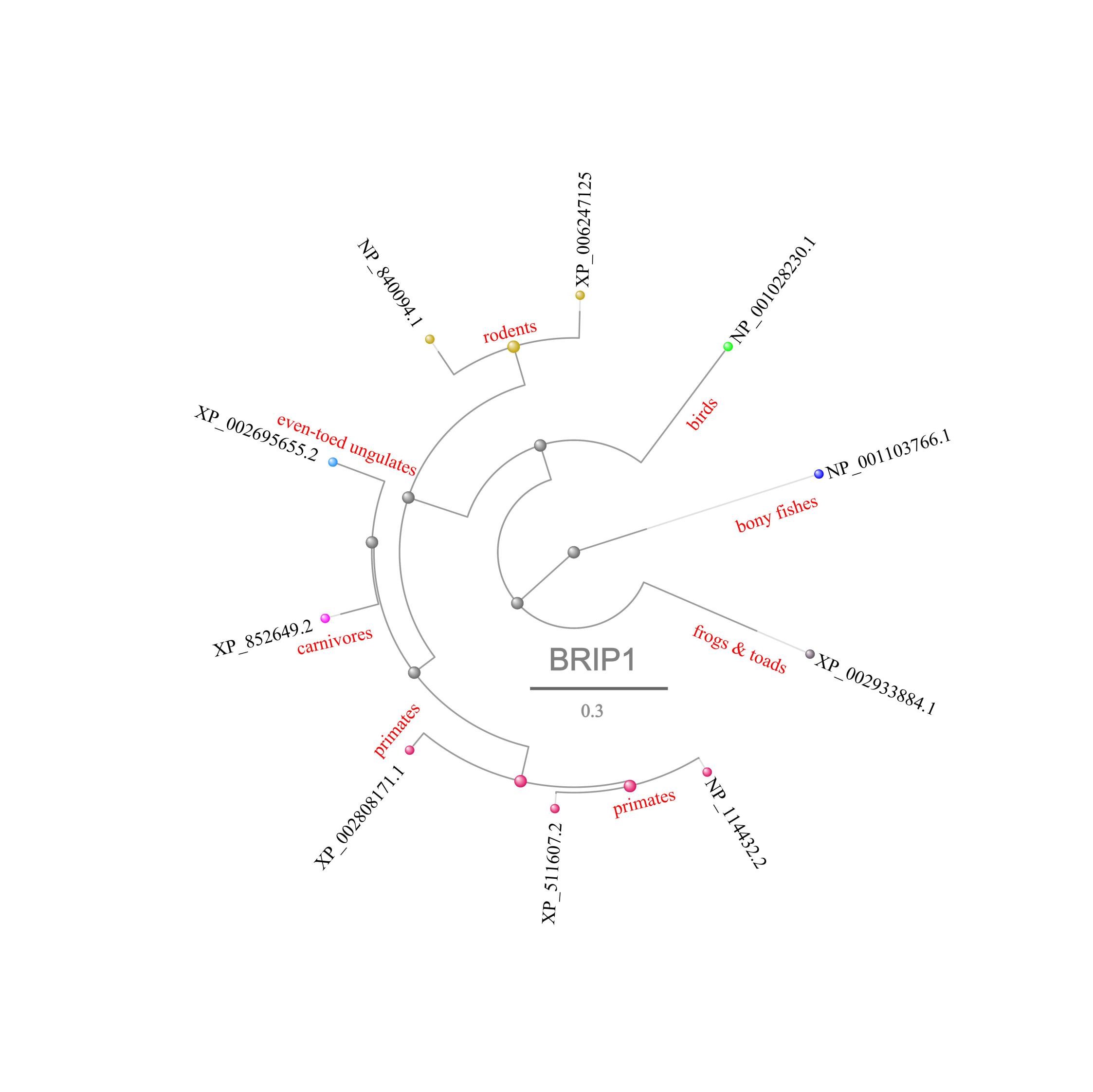

Supplement: Supplementary file 4 — Additional file 4: Figure S4. Phylogenetic tree of BRIP1. We used a constraint-based multiple alignment online tool of NCBI to get the phylogenetic tree of BRIP1 in different species. [file 12957_2022_2877_MOESM4_ESM.tiff]

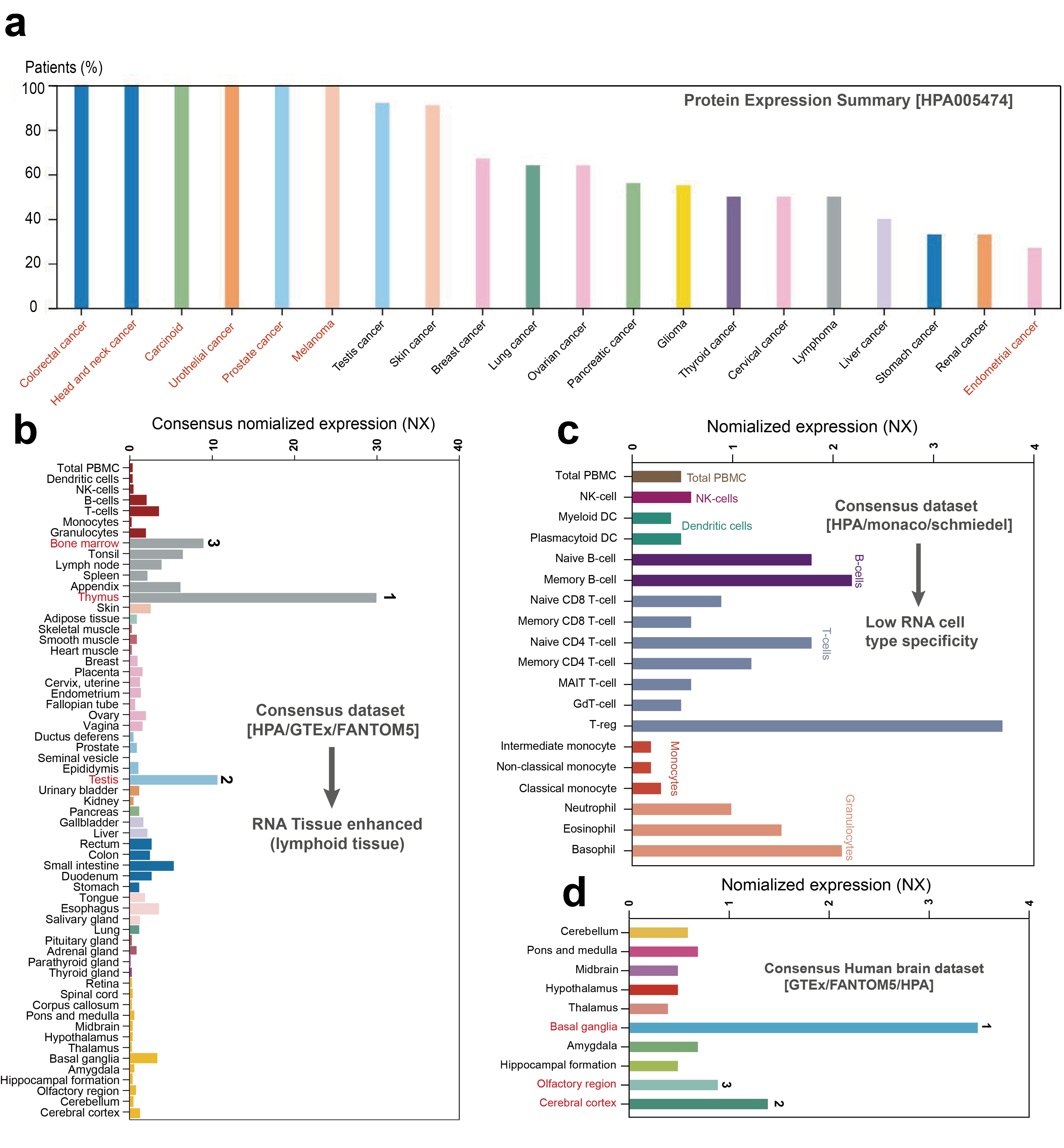

Supplement: Supplementary file 5 — Additional file 5: Figure S5. The expression levels of BRIP1 in different tumors and in different tissues, blood cells, and brain tissues under the normal physiological state. We analyzed the expression of the BRIP1 gene in different tumors using antibodies of HPA005474 (a), using the consensus datasets of HPA, GTEx, and FANTOM5 to explore the expression of BRIP1 in different tissues (b) and brain tissues (d), using the consensus datasets of HPA, Monaco, and Schmieder to explore the expression of BRIP1 in different blood cells (c). [file 12957_2022_2877_MOESM5_ESM.tiff]

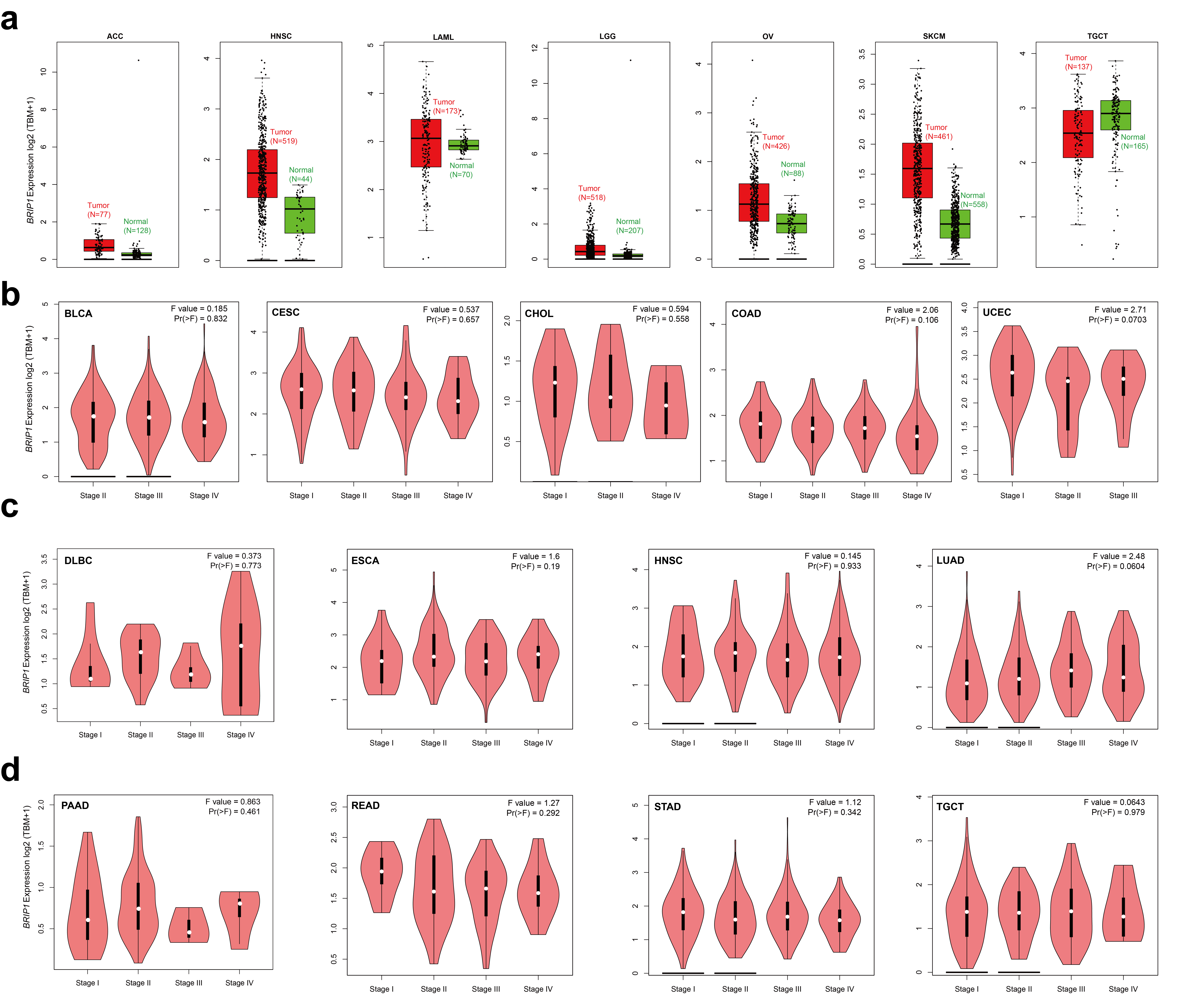

Supplement: Supplementary file 6 — Additional file 6: Figure S6. The expression levels of BRIP1 in various cancers and pathological stages. (a) The expression levels of BRIP1 in ACC, HNSC, LAML, LGG, OV, SKCM, and TGCT in the TCGA project were compared with the corresponding normal tissues of the GTEx databases. The expression levels of BRIP1 by different pathological stages of BLCA, CESC, CHOL, COAD, UCEC (b); DLBC, ESCA, HNSC, LUAD (c); and PAAD, READ, STAD, TGCT (d). [file 12957_2022_2877_MOESM6_ESM.tiff]

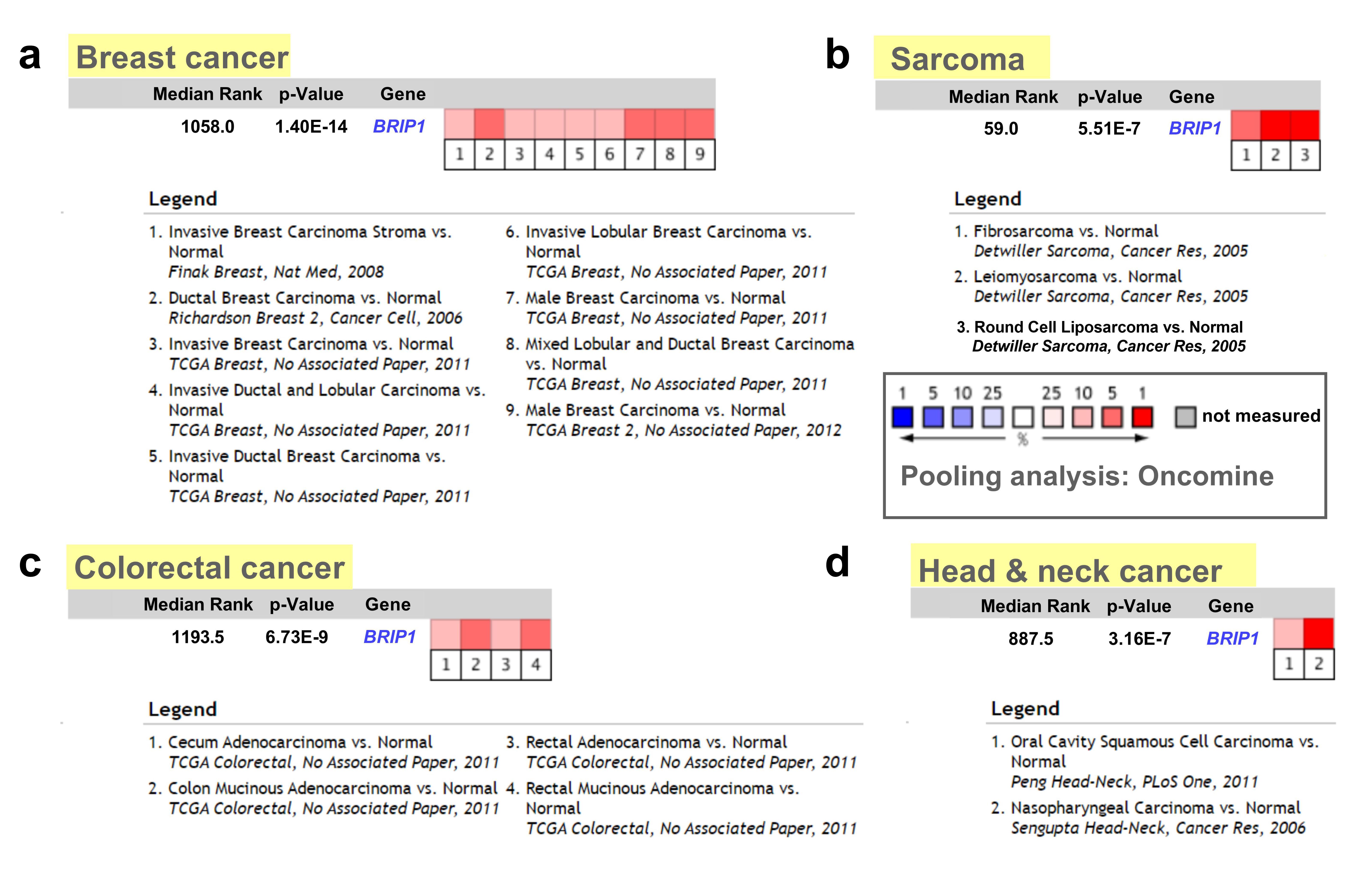

Supplement: Supplementary file 7 — Additional file 7: Figure S7. Pooled analysis on the differential BRIP1 expression between normal and tumor tissues via the ONCOMINE database. (a) Breast cancer; (b) Sarcoma; (c) colorectal cancer; (d) Head & neck cancer. [file 12957_2022_2877_MOESM7_ESM.tiff]

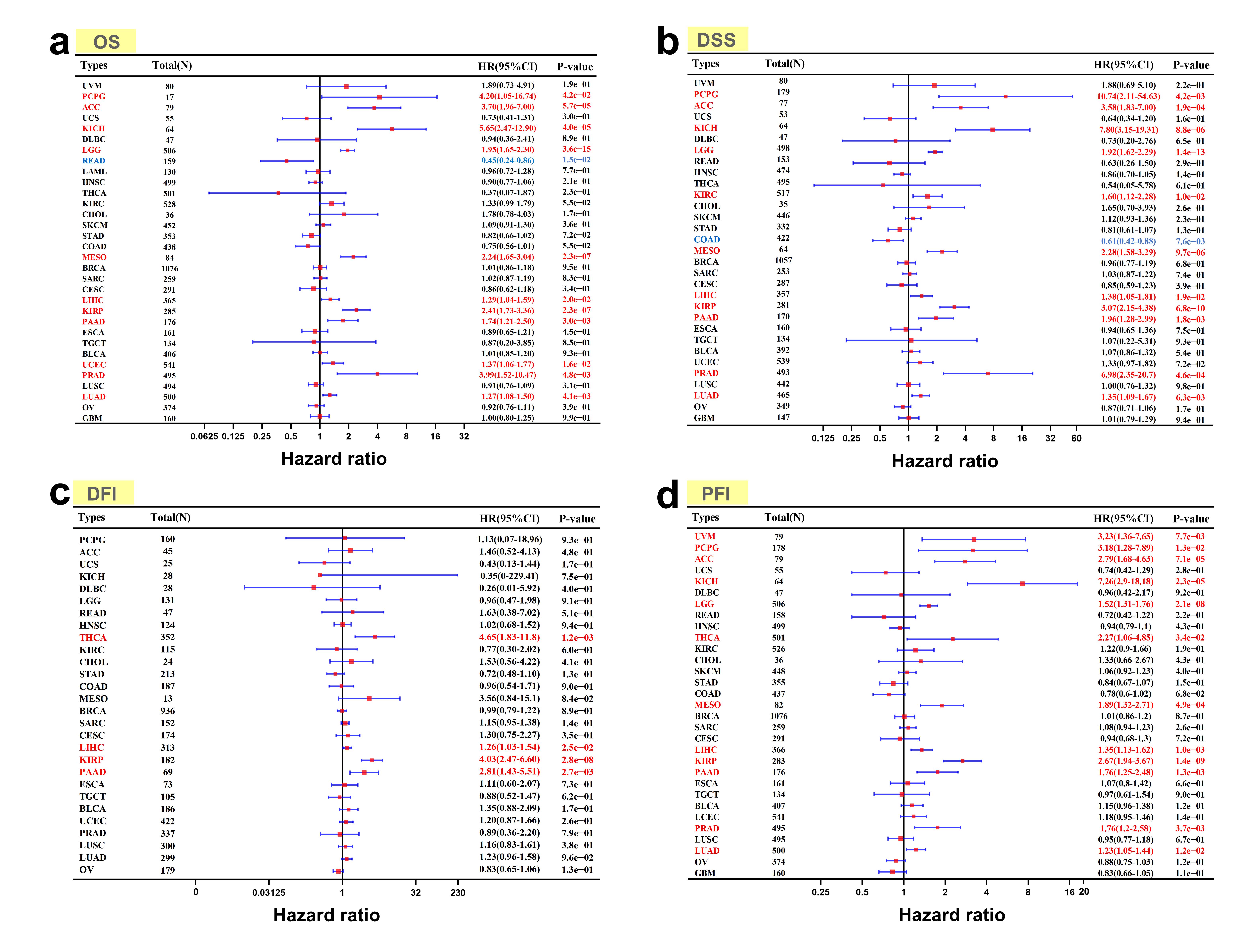

Supplement: Supplementary file 11 — Additional file 11: Figure S11. Forest plots depicting the prognostic role of BRIP1 in various TCGA tumors. The hazard ratio of OS (a), DSS (b), DFI (c), and PFI (d) for patients with differential BRIP1 expression across TCGA tumors. [file 12957_2022_2877_MOESM11_ESM.tiff]

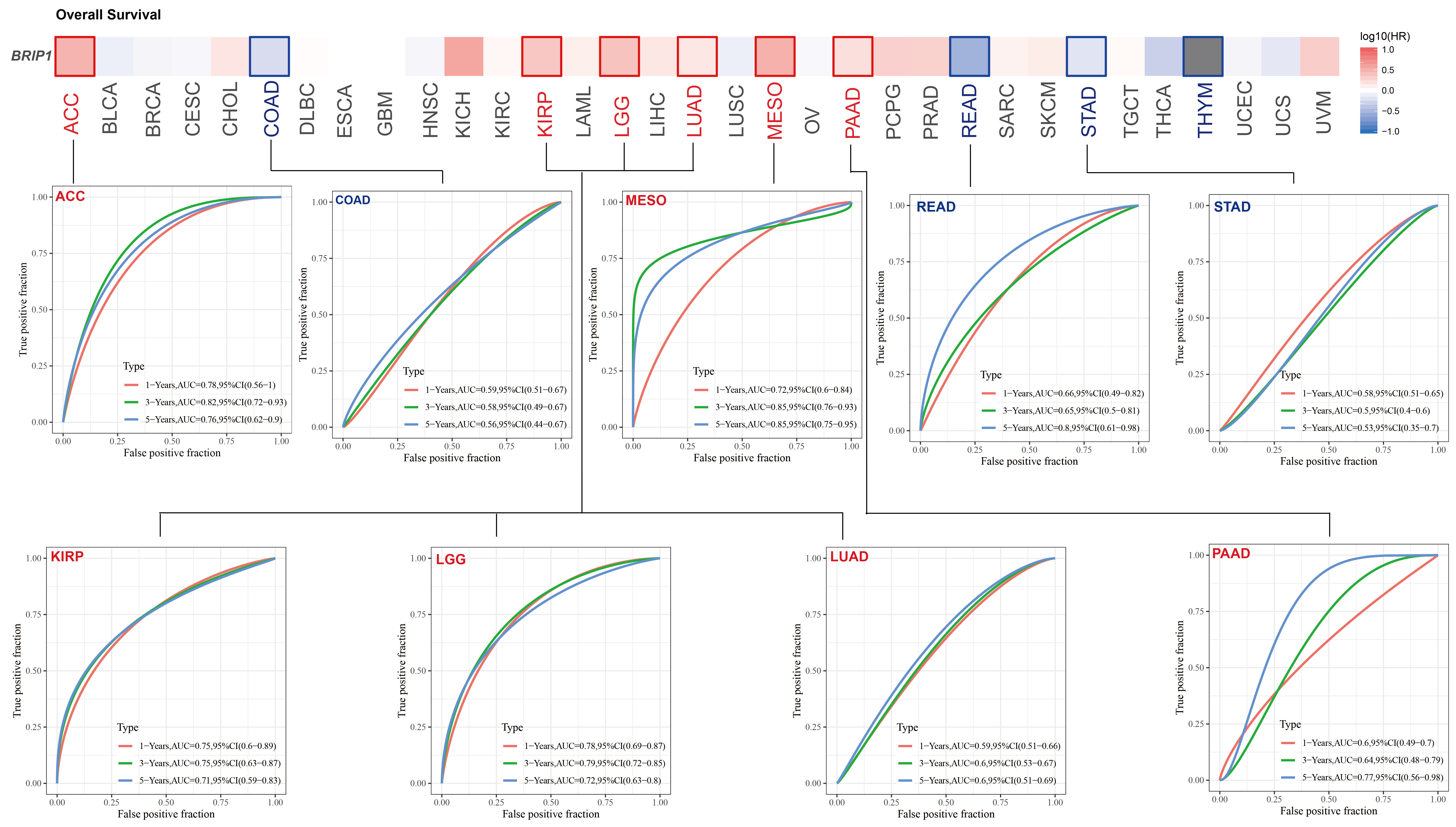

Supplement: Supplementary file 12 — Additional file 12: Figure S12. Correlation between BRIP1 expression and survival prognosis of cancers in TCGA. We used the GEPIA2 tool to perform overall survival analyses of different tumors in TCGA by BRIP1 gene expression. The cancer types with positive results were highlighted. We used Sangerbox online tool to obtain the ROC curves of OS for the positive cancer types from GEPIA2 (THYM was not available). [file 12957_2022_2877_MOESM12_ESM.tiff]

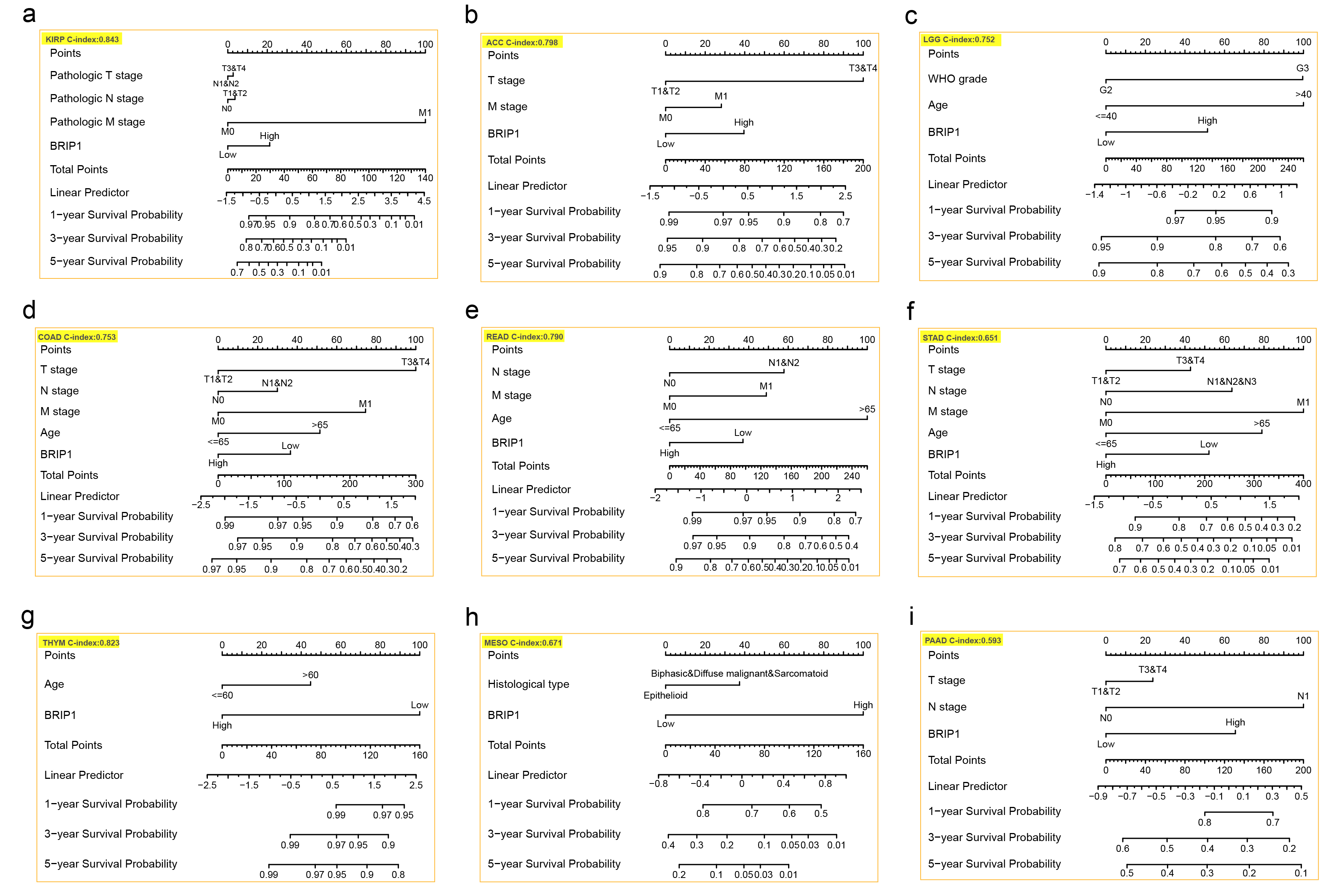

Supplement: Supplementary file 13 — Additional file 13: Figure S13. Regression-based prognostic nomograms for different TCGA tumors. Clinical characteristics which were significantly associated with OS and BRIP1 expression were used to construct the prognostic nomograms. [file 12957_2022_2877_MOESM13_ESM.tiff]

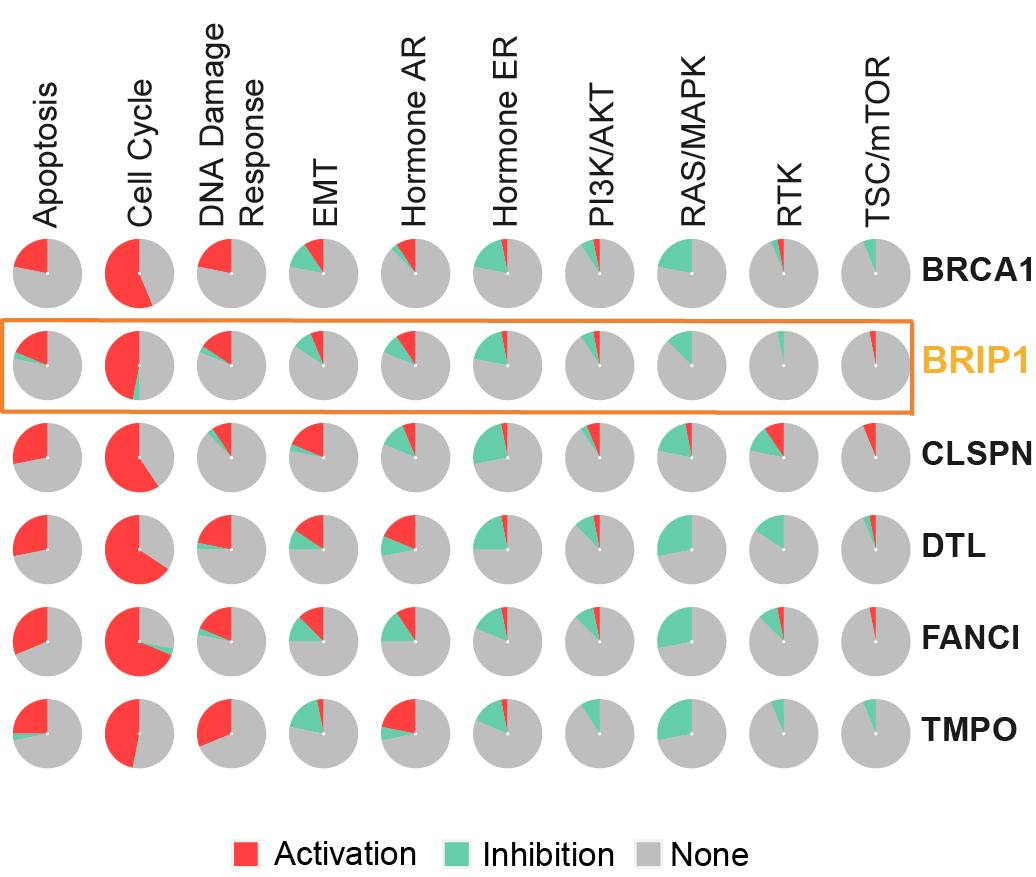

Supplement: Supplementary file 14 — Additional file 14: Figure S14. The proportion of cancer types with BRIP1 and its most correlated five genes are significantly associated with activation (red) or inhibition (blue) of the ten key signaling pathways in 31 cancer types. [file 12957_2022_2877_MOESM14_ESM.tiff]

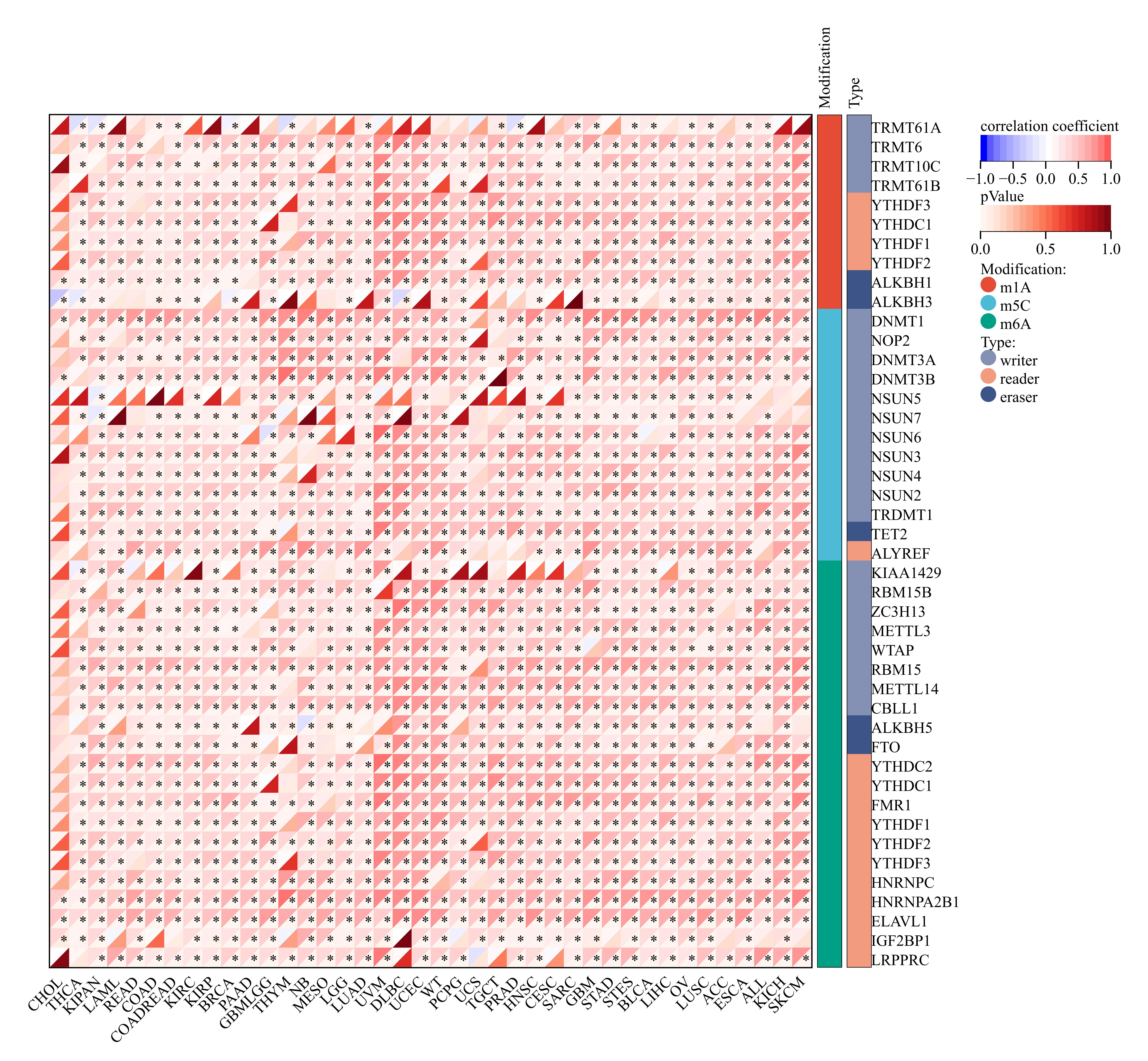

Supplement: Supplementary file 15 — Additional file 15: Figure S15. Correlation analysis between the expression of BRIP1 and marker genes of RNA modification. [file 12957_2022_2877_MOESM15_ESM.tiff]

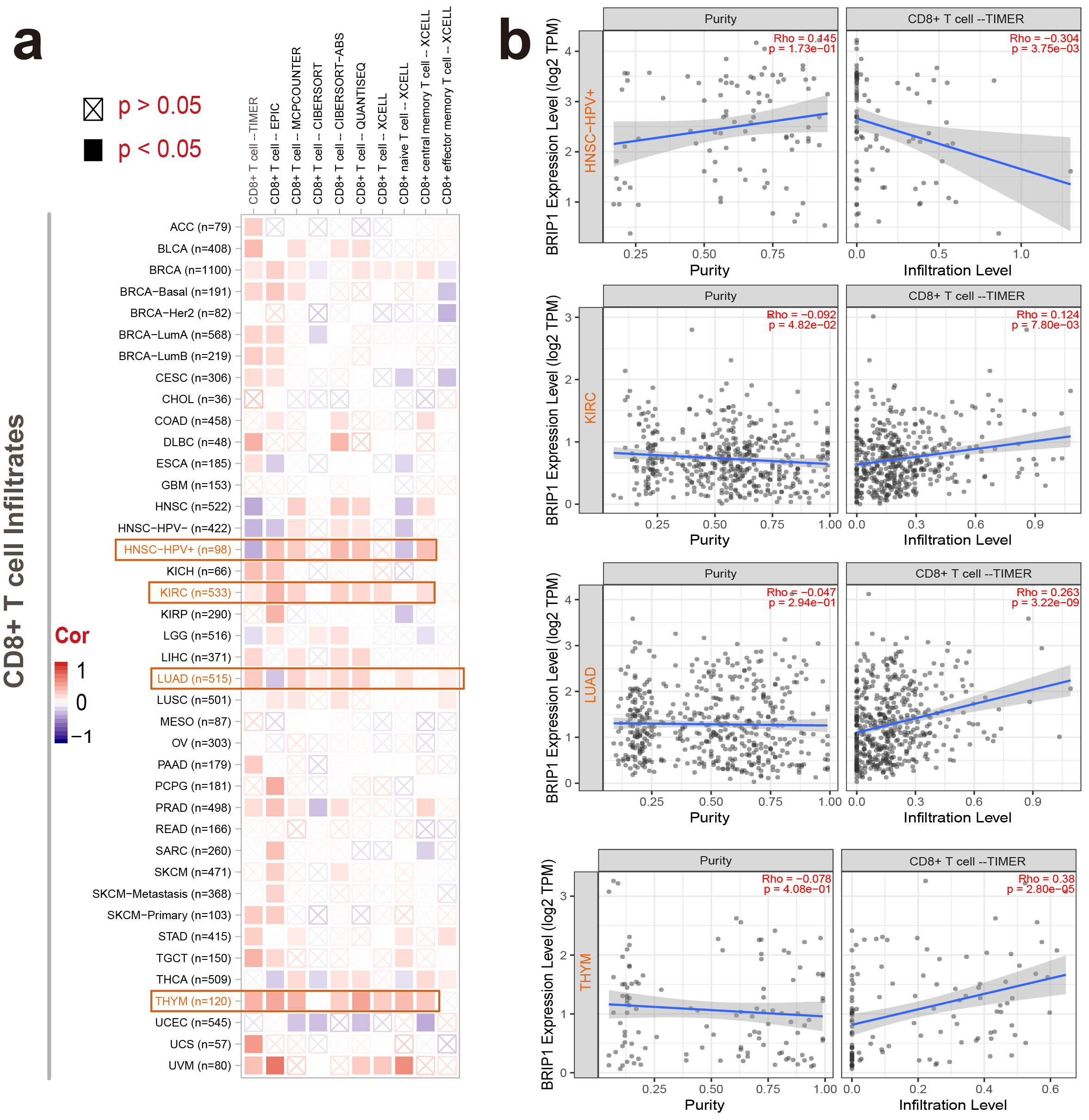

Supplement: Supplementary file 16 — Additional file 16: Figure S16. Correlation analysis between BRIP1 expression and immune infiltration of CD8+ T-cells. Different algorithms were used to explore the potential correlation between the expression levels of BRIP1 and the infiltration levels of CD8+ T-cells across all types of cancer in TCGA. [file 12957_2022_2877_MOESM16_ESM.tiff]

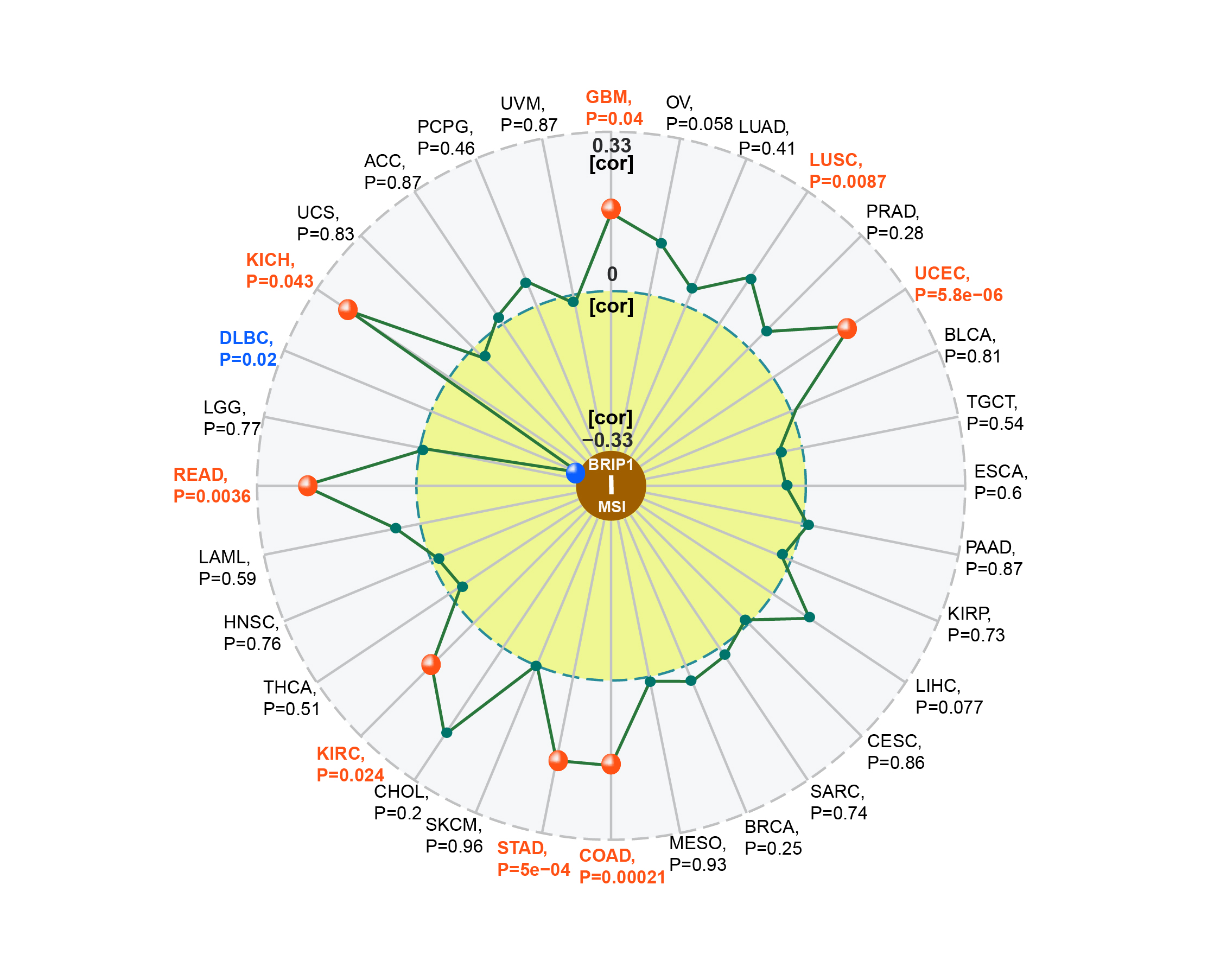

Supplement: Supplementary file 17 — Additional file 17: Figure S17. Correlation between BRIP1 expression and microsatellite instability. Based on the different tumors of TCGA, we explored the potential correlation between BRIP1 expression and microsatellite instability (MSI). The P-value is supplied. The partial correlation (cor) values of +0.33 and -0.33 are marked. [file 12957_2022_2877_MOESM17_ESM.tiff]

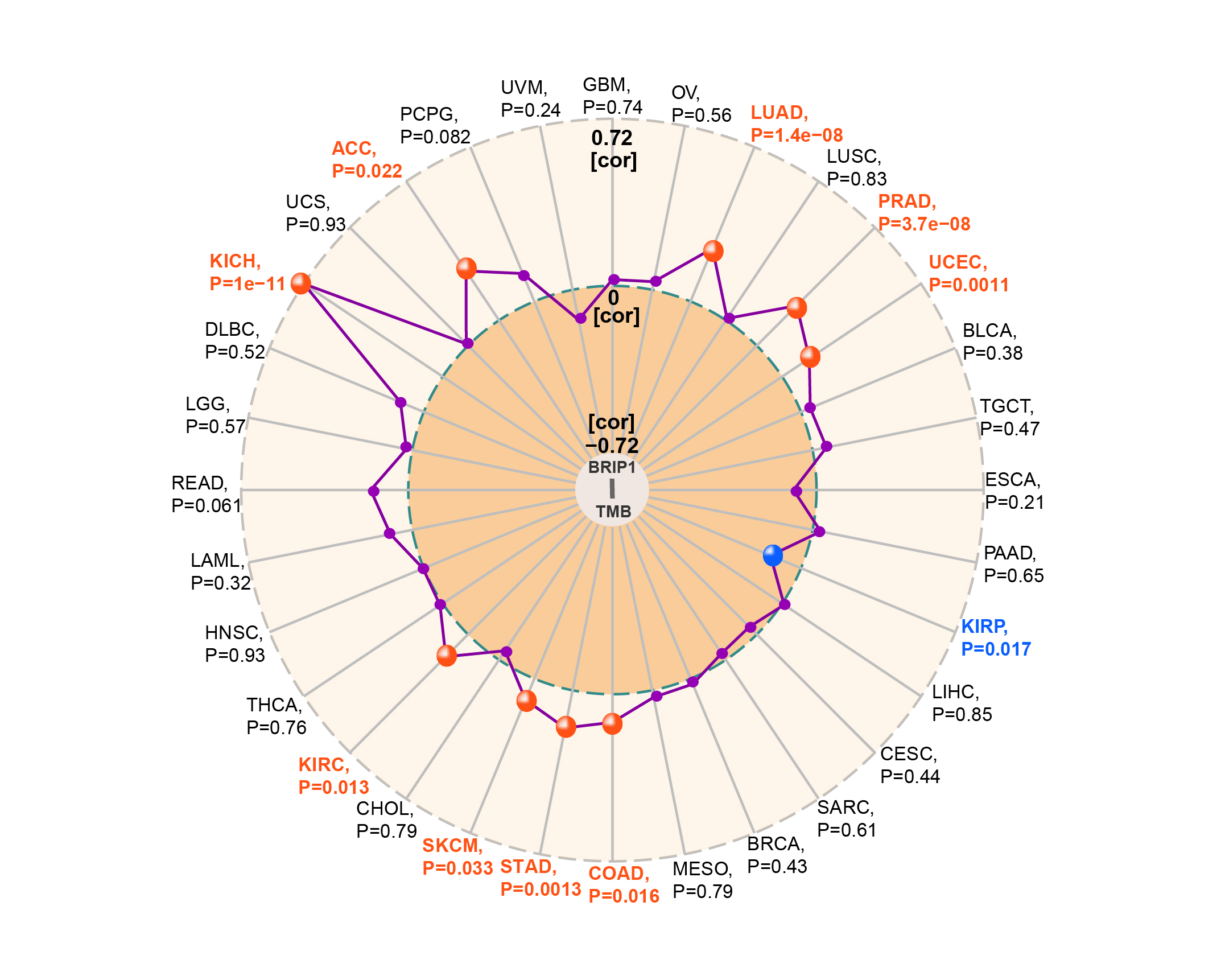

Supplement: Supplementary file 18 — Additional file 18: Figure S18. Correlation between BRIP1 expression and tumor mutational burden. Based on the different tumors of TCGA, we explored the potential correlation between BRIP1 expression and tumor mutational burden (TMB). The P-value is supplied. The partial correlation (cor) values of +0.72 and -0.72 are marked. [file 12957_2022_2877_MOESM18_ESM.tiff]

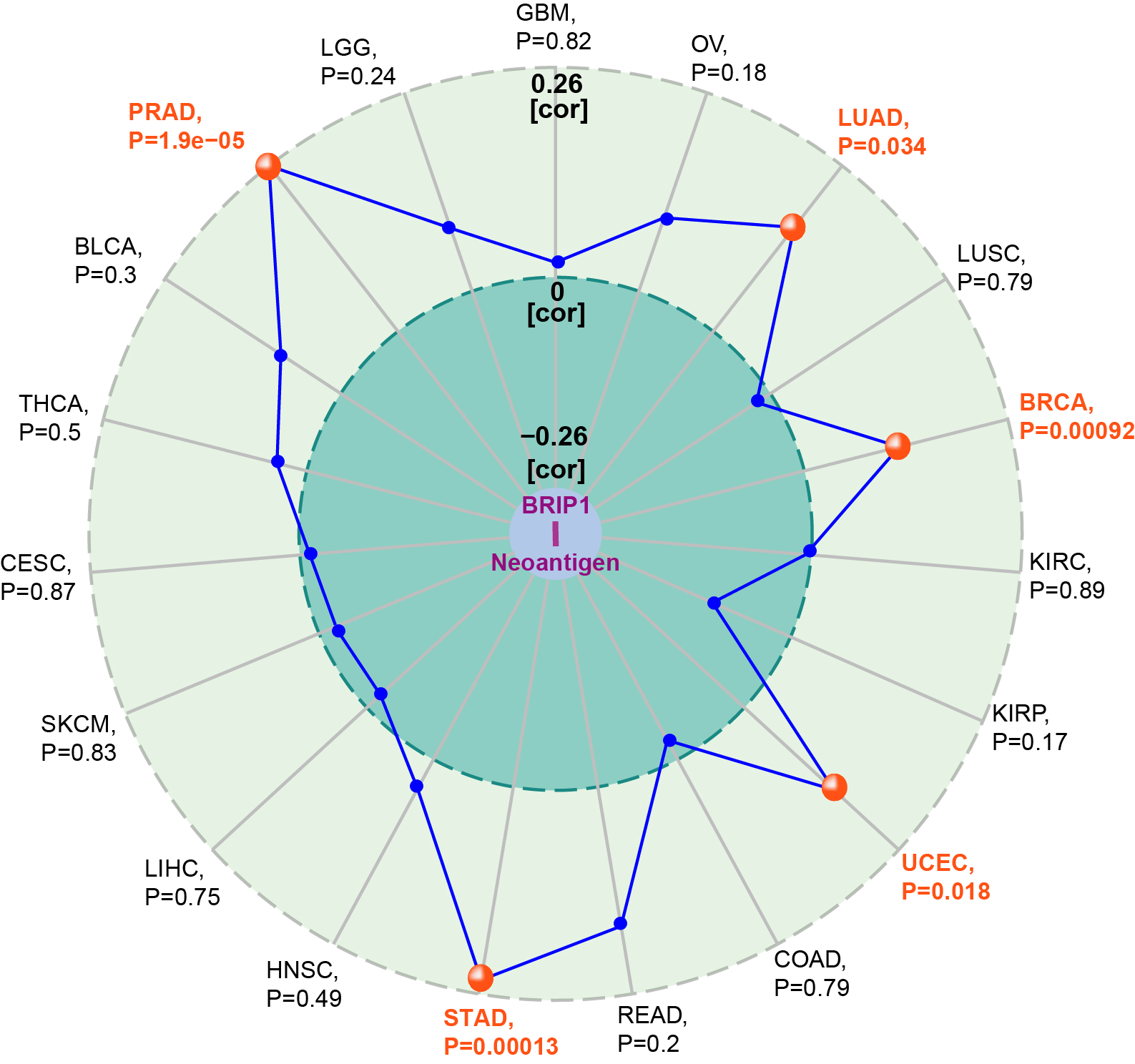

Supplement: Supplementary file 19 — Additional file 19: Figure S19. Correlation between BRIP1 expression and neoantigen. Based on the different tumors of TCGA, we explored the potential correlation between BRIP1 expression and neoantigen. The P-value is supplied. The partial correlation (cor) values of +0.26 and -0.26 are marked. [file 12957_2022_2877_MOESM19_ESM.tiff]

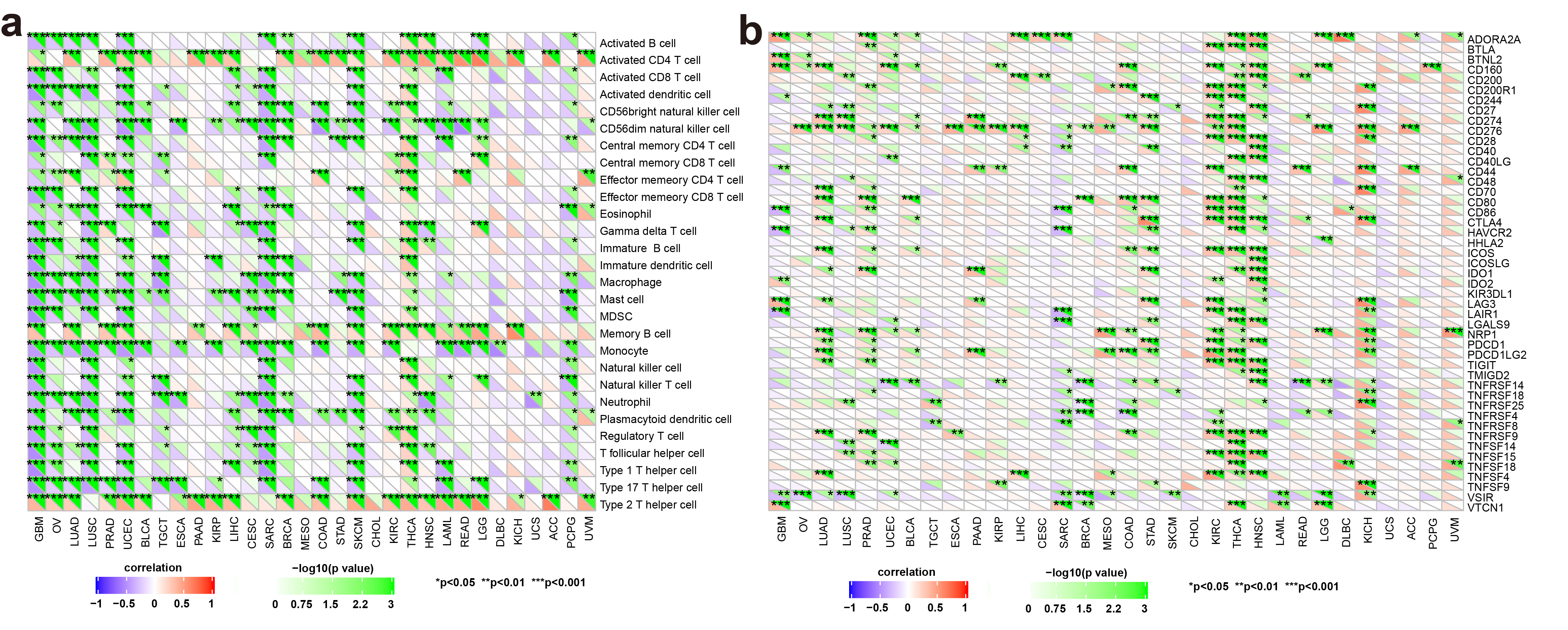

Supplement: Supplementary file 20 — Additional file 20: Figure S20. Correlation between BRIP1 expression, immune pathways (a), and immune checkpoints (b) across all TCGA tumors. [file 12957_2022_2877_MOESM20_ESM.tiff]
