# Supplementary material for: Multimolecular characteristics and role of BRCA1 interacting protein C-terminal helicase 1 (BRIP1) in human tumors: a pan-cancer analysis
Source: World J Surg Oncol. 2023 Mar 13;21:91. doi: 10.1186/s12957-022-02877-8 (PMC10010046; doi:10.1186/s12957-022-02877-8)
Supplement: Supplementary file 21 — Additional file 21: Table S1. The significant changes of BRIP1 expression in transcription level in pan-cancers (ONCOMINE database). Table S2. Univariate and Multivariate COX regression for clinical characteristics and BRIP1 expression in various tumors. Table S3. Enrichment analysis of BRIP1-related genes across pancancer. Table S4. Association between BRIP1 DNA methylation and gene expression for the 24 tumors of TCGA. Table S5. The correlation between BRIP1 and other genes of ACC from the TCGA database. Table S6. The correlation between BRIP1 and other genes of BLCA from the TCGA database. Table S7. The correlation between BRIP1 and other genes of LUSC from the TCGA database. Table S8. The correlation between BRIP1 and other genes of OV from the TCGA database. Table S9. The correlation between BRIP1 and other genes of THCA from the TCGA database. Table S10. The correlation between BRIP1 and other genes of UCEC from the TCGA database. Table S11. The correlation between BRIP1 and other genes of BRCA from the TCGA database. Table S12. The correlation between BRIP1 and other genes of COAD, READ from the TCGA database. Table S13. The correlation between BRIP1 and other genes of ESCA with STAD from the TCGA database. Table S14. The correlation between BRIP1 and other genes of GBM from the TCGA database. Table S15. The correlation between BRIP1 and other genes of KIRC from the TCGA database. Table S16. The correlation between BRIP1 and other genes of LGG from the TCGA database. Table S17. The correlation between BRIP1 and other genes of LUAD from the TCGA database. Table S18. The correlation between BRIP1 and other genes of PRAD from the TCGA database. Table S19. The correlation between BRIP1 and other genes of STAD from the TCGA database. [file 12957_2022_2877_MOESM21_ESM.doc]

Multimolecular characteristics and role of BRCA1 Interacting Protein C-Terminal Helicase 1 (BRIP1) in human tumors: a pan-cancer analysis

**Supplementary data**

**Supplementary Methods**

**Genome location, protein structure, and phylogenetic tree of BRIP1**

We logged into the UCSC ^1^ (<http://genome.ucsc.edu/>) genome browser to get the information on the BRIP1 genome location. The sequence and conserved domain architectures of BRIP1 in various species were obtained by applying the “HomoloGene” function of the National Center for Biotechnology Information (NCBI) (<https://www.ncbi.nlm.nih.gov/homologene/>). After retrieving the protein sequences of different species, we used the constraint-based multiple alignment online tool of NCBI (<https://www.ncbi.nlm.nih.gov/tools/cobalt/>) to draw the phylogenetic tree of BRIP1, and the circular tree layout was selected.

**Differential expression analysis of BRIP1**

We used the TISIDB database^2^, which can be used for tumor and immune system interaction analysis, to compare BRIP1 expression in various molecular and immune subtypes of TCGA cancer. By querying “BRIP1” in the “Quick Search” panel, we can get the distribution of BRIP1 expression across molecular and immune subtypes in the “Subtype” module.

**Survival prognosis analysis of BRIP1**

We utilized the online tool Kaplan-Meier Plotter (<http://kmplot.com/analysis/>) to assess the survival status of patients with various cancers based on the information from TCGA and GEO databases. ^3^ OS, PPS (post-progression survival), DMFS (distant metastasis-free survival), PFS (progress-free survival), FP (first progression), and RFS (relapse-free survival) analyses were included. We selected the “auto select best cutoff” option to split patients with breast, ovarian, lung, gastric, and liver cancer. The generated Kaplan-Meier plots indicated log-rank P-value, HR (hazard ratio), and 95% CI (confidence intervals).

To verify the prognostic value of BRIP1 across all TCGA tumors, we used Sangerbox tools^4^ (<http://sangerbox.com/Tool>), a free online platform to evaluate the OS, DSS (disease-specific survival), DFI (disease-free interval), and PFI (progress-free interval) of patients with differential BRIP1 expression in the “Gene-KM” module, *P*-value, HR, and 95% CI were also supplied. Besides, in the same module of Sangerbox, ROC curves of OS with values of 1-year, 3-year, and 5-year’s AUC (area under the curve) and 95%CI were indicated.

We also used the “survival” package of R software to conduct a cox regression analysis to explore the survival role of BRIP1 in various tumors. Clinical characteristics such as pathological stages (T/N/M), WHO grade, histological type, age, and gender were included. Based on the above Univariate analysis results, we combined the parameters with statistical significance in different cancers to devise the prognostic nomograms, “rms” and “survival” packages of R software were applied, and the index of concordance (C-index) for each cancer was supplied.

**Immune analysis of BRIP1**

By logging into the Sangerbox, we can explore the possible association between Microsatellite instability (MSI), Tumor Mutational Burden (TMB), neoantigen, and BRIP1 expression across all TCGA cancers in the “Gene-Immune” section under the “Gene” module. Using Pearson correlation analysis, we obtained the value of partial correlation and *P*-value, and the figures generated were modified by Adobe Illustrator CC (64Bit). Besides, we also explored the relationship between BRIP1 expression, immune pathways, and immune checkpoints across all TCGA tumors, with the query “BRIP1” in the “Gene-Immune” section.

**Supplementary figure legends**

**Figure S1.** Role of BRIP1 in Fanconi anemia pathway.

**Figure S2.** Study design of the current study. CNA: Copy number alteration; SNV: Single nucleotide variation; CNV: Copy number variation. PPI: Protein-protein interaction; KEGG: Kyoto Encyclopedia of Genes and Genomes; GO: Gene Oncology; MF: molecular functions; CC: cellular components; BP: biological processes; MSI: Microsatellite instability; TMB: Tumor mutational burden.

**Figure S3.** Structural characteristics of BRIP1 in various species. (a) Genomic location of human BRIP1; (b) Conserved domains of BRIP1 protein among different species.

**Figure S4.** Phylogenetic tree of BRIP1. We used a constraint-based multiple alignment online tool of NCBI to get the phylogenetic tree of BRIP1 in different species.

**Figure S5.** The expression levels of BRIP1 in different tumors and in different tissues, blood cells, and brain tissues under the normal physiological state. We analyzed the expression of the BRIP1 gene in different tumors using antibodies of HPA005474 (a), using the consensus datasets of HPA, GTEx, and FANTOM5 to explore the expression of BRIP1 in different tissues (b) and brain tissues (d), using the consensus datasets of HPA, Monaco, and Schmieder to explore the expression of BRIP1 in different blood cells (c).

**Figure S6.** The expression levels of BRIP1 in various cancers and pathological stages. (a) The expression levels of BRIP1 in ACC, HNSC, LAML, LGG, OV, SKCM, and TGCT in the TCGA project were compared with the corresponding normal tissues of the GTEx databases. The expression levels of BRIP1 by different pathological stages of BLCA, CESC, CHOL, COAD, UCEC (b); DLBC, ESCA, HNSC, LUAD (c); and PAAD, READ, STAD, TGCT (d).

**Figure S7.** Pooled analysis on the differential BRIP1 expression between normal and tumor tissues via the ONCOMINE database. (a) Breast cancer; (b) Sarcoma; (c) colorectal cancer; (d) Head & neck cancer.

**Figure S8.** Differential expression analysis of BRIP1 in various subtypes across different cancers via TISIDB. Cancer types with statistical significance were shown. (a) The expression of BRIP1 in different molecular subtypes and (b) immune subtypes of cancers.

**Figure S9.** Differential expression analysis of BRIP1 in various subtypes across different cancers via TISIDB. Cancer types without statistical significance were shown. (a) The expression of BRIP1 in different molecular subtypes and (b) immune subtypes of cancers.

**Figure S10.** Correlation between BRIP1 expression and prognosis of cancers using the Kaplan-Meier plotter. We used the Kaplan-Meier plotter to assess the survival status of patients via the expression levels of the BRIP1 gene in breast cancer (a), ovarian cancer (b), lung cancer (c), gastric cancer (d), and liver cancer (e) cases, including OS, PPS, DMFS, PFS, FP, and RFS.

**Figure S11.** Forest plots depicting the prognostic role of BRIP1 in various TCGA tumors. The hazard ratio of OS (a), DSS (b), DFI (c), and PFI (d) for patients with differential BRIP1 expression across TCGA tumors.

**Figure S12.** Correlation between BRIP1 expression and survival prognosis of cancers in TCGA. We used the GEPIA2 tool to perform overall survival analyses of different tumors in TCGA by BRIP1 gene expression. The cancer types with positive results were highlighted. We used Sangerbox online tool to obtain the ROC curves of OS for the positive cancer types from GEPIA2 (THYM was not available).

**Figure S13.** Regression-based prognostic nomograms for different TCGA tumors. Clinical characteristics which were significantly associated with OS and BRIP1 expression were used to construct the prognostic nomograms.

**Figure S14.** The proportion of cancer types with BRIP1 and its most correlated five genes are significantly associated with activation (red) or inhibition (blue) of the ten key signaling pathways in 31 cancer types.

**Figure S15.** Correlation analysis between the expression of BRIP1 and marker genes of RNA modification.

**Figure S16.** Correlation analysis between BRIP1 expression and immune infiltration of CD8+ T-cells. Different algorithms were used to explore the potential correlation between the expression levels of BRIP1 and the infiltration levels of CD8+ T-cells across all types of cancer in TCGA.

**Figure S17.** Correlation between BRIP1 expression and microsatellite instability. Based on the different tumors of TCGA, we explored the potential correlation between BRIP1 expression and microsatellite instability (MSI). The *P*-value is supplied. The partial correlation (cor) values of +0.33 and -0.33 are marked.

**Figure S18.** Correlation between BRIP1 expression and tumor mutational burden. Based on the different tumors of TCGA, we explored the potential correlation between BRIP1 expression and tumor mutational burden (TMB). The *P*-value is supplied. The partial correlation (cor) values of +0.72 and -0.72 are marked.

**Figure S19.** Correlation between BRIP1 expression and neoantigen. Based on the different tumors of TCGA, we explored the potential correlation between BRIP1 expression and neoantigen. The *P*-value is supplied. The partial correlation (cor) values of +0.26 and -0.26 are marked.

**Figure S20.** Correlation between BRIP1 expression, immune pathways (a), and immune checkpoints (b) across all TCGA tumors.

**Supplementary table**

**Table S1**. The significant changes of BRIP1 expression in transcription level in pan-cancers (ONCOMINE database)

**Table S2.** Univariate and Multivariate COX regression for clinical characteristics and BRIP1 expression in various tumors.

**Table S3.** Enrichment analysis of BRIP1-related genes across pancancer.

**Table S4.** Association between BRIP1 DNA methylation and gene expression for the 24 tumors of TCGA.

**Table S5.** The correlation between BRIP1 and other genes of ACC from the TCGA database.

**Table S6.** The correlation between BRIP1 and other genes of BLCA from the TCGA database.

**Table S7.** The correlation between BRIP1 and other genes of LUSC from the TCGA database.

**Table S8.** The correlation between BRIP1 and other genes of OV from the TCGA database.

**Table S9.** The correlation between BRIP1 and other genes of THCA from the TCGA database.

**Table S10.** The correlation between BRIP1 and other genes of UCEC from the TCGA database.

**Table S11.** The correlation between BRIP1 and other genes of BRCA from the TCGA database.

**Table S12.** The correlation between BRIP1 and other genes of COAD, READ from the TCGA database.

**Table S13.** The correlation between BRIP1 and other genes of ESCA with STAD from the TCGA database.

**Table S14.** The correlation between BRIP1 and other genes of GBM from the TCGA database.

**Table S15.** The correlation between BRIP1 and other genes of KIRC from the TCGA database.

**Table S16.** The correlation between BRIP1 and other genes of LGG from the TCGA database.

**Table S17.** The correlation between BRIP1 and other genes of LUAD from the TCGA database.

**Table S18.** The correlation between BRIP1 and other genes of PRAD from the TCGA database.

**Table S19.** The correlation between BRIP1 and other genes of STAD from the TCGA database.

**Reference**

1. W.J. Kent, C.W. Sugnet, T.S. Furey, K.M. Roskin, T.H. Pringle, A.M. Zahler, D. Haussler. The human genome browser at UCSC, Genome Res. 12 (2002) 996-1006.

2. Ru B, Wong CN, Tong Y, Zhong JY, Zhong SSW, Wu WC, Chu KC, Wong CY, Lau CY, Chen I, et al. TISIDB: an integrated repository portal for tumor-immune system interactions. Bioinformatics. 2019;35(20):4200–4202. doi:10.1093/bioinfor-matics/btz210.

3. Nagy A, Munkárcsy G, Győrffy B. Pancancer survival analysis of cancer hallmark genes, Scientific Reports, 2021 Mar 15;11(1):6047. doi: 10.1038/s41598-021-84787-5.

4.R. Bonneville, M.A. Krook, E.A. Kautto, J. Miya, M.R. Wing, H.Z. Chen, J.W. Reeser, L. Yu, S. Roychowdhury. Landscape of Microsatellite Instability Across 39 Cancer Types, JCO Precis Oncol. 2017 (2017).

5. Sun L, Hui AM, Su Q, Vortmeyer A, Kotliarov Y, Pastorino S, Passaniti A, Menon J, Walling J, Bailey R, Rosenblum M, Mikkelsen T, Fine HA. Neuronal and glioma-derived stem cell factor induces angiogenesis within the brain. Cancer Cell. 2006 Apr;9(4):287-300. doi: 10.1016/j.ccr.2006.03.003. PMID: 16616334.

6. Lee J, Kotliarova S, Kotliarov Y, Li A, Su Q, Donin NM, Pastorino S, Purow BW, Christopher N, Zhang W, Park JK, Fine HA. Tumor stem cells derived from glioblastomas cultured in bFGF and EGF more closely mirror the phenotype and genotype of primary tumors than do serum-cultured cell lines. Cancer Cell. 2006 May;9(5):391-403. doi: 10.1016/j.ccr.2006.03.030. PMID: 16697959.

7. Richardson AL, Wang ZC, De Nicolo A, Lu X, Brown M, Miron A, Liao X, Iglehart JD, Livingston DM, Ganesan S. X chromosomal abnormalities in basal-like human breast cancer. Cancer Cell. 2006 Feb;9(2):121-32. doi: 10.1016/j.ccr.2006.01.013. PMID: 16473279.

8. Finak G, Bertos N, Pepin F, Sadekova S, Souleimanova M, Zhao H, Chen H, Omeroglu G, Meterissian S, Omeroglu A, Hallett M, Park M. Stromal gene expression predicts clinical outcome in breast cancer. Nat Med. 2008 May;14(5):518-27. doi: 10.1038/nm1764. Epub 2008 Apr 27. PMID: 18438415.

9. Scotto L, Narayan G, Nandula SV, Arias-Pulido H, Subramaniyam S, Schneider A, Kaufmann AM, Wright JD, Pothuri B, Mansukhani M, Murty VV. Identification of copy number gain and overexpressed genes on chromosome arm 20q by an integrative genomic approach in cervical cancer: potential role in progression. Genes Chromosomes Cancer. 2008 Sep;47(9):755-65. doi: 10.1002/gcc.20577. PMID: 18506748.

10. Cui J, Chen Y, Chou WC, Sun L, Chen L, Suo J, Ni Z, Zhang M, Kong X, Hoffman LL, Kang J, Su Y, Olman V, Johnson D, Tench DW, Amster IJ, Orlando R, Puett D, Li F, Xu Y. An integrated transcriptomic and computational analysis for biomarker identification in gastric cancer. Nucleic Acids Res. 2011 Mar;39(4):1197-207. doi: 10.1093/nar/gkq960. Epub 2010 Oct 21. PMID: 20965966; PMCID: PMC3045610.

11. Sengupta S, den Boon JA, Chen IH, Newton MA, Dahl DB, Chen M, Cheng YJ, Westra WH, Chen CJ, Hildesheim A, Sugden B, Ahlquist P. Genome-wide expression profiling reveals EBV-associated inhibition of MHC class I expression in nasopharyngeal carcinoma. Cancer Res. 2006 Aug 15;66(16):7999-8006. doi: 10.1158/0008-5472.CAN-05-4399. PMID: 16912175.

12. Peng CH, Liao CT, Peng SC, Chen YJ, Cheng AJ, Juang JL, Tsai CY, Chen TC, Chuang YJ, Tang CY, Hsieh WP, Yen TC. A novel molecular signature identified by systems genetics approach predicts prognosis in oral squamous cell carcinoma. PLoS One. 2011;6(8):e23452. doi: 10.1371/journal.pone.0023452. Epub 2011 Aug 11. PMID: 21853135; PMCID: PMC3154947.

13. Detwiller KY, Fernando NT, Segal NH, Ryeom SW, D'Amore PA, Yoon SS. Analysis of hypoxia-related gene expression in sarcomas and effect of hypoxia on RNA interference of vascular endothelial cell growth factor A. Cancer Res. 2005 Jul 1;65(13):5881-9. doi: 10.1158/0008-5472.CAN-04-4078. PMID: 15994966.

14. Buchholz M, Braun M, Heidenblut A, Kestler HA, Klöppel G, Schmiegel W, Hahn SA, Lüttges J, Gress TM. Transcriptome analysis of microdissected pancreatic intraepithelial neoplastic lesions. Oncogene. 2005 Oct 6;24(44):6626-36. doi: 10.1038/sj.onc.1208804. PMID: 16103885.

15. Skotheim RI, Lind GE, Monni O, Nesland JM, Abeler VM, Fosså SD, Duale N, Brunborg G, Kallioniemi O, Andrews PW, Lothe RA. Differentiation of human embryonal carcinomas in vitro and in vivo reveals expression profiles relevant to normal development. Cancer Res. 2005 Jul 1;65(13):5588-98. doi: 10.1158/0008-5472.CAN-05-0153. PMID: 15994931.

| Table S1. The significant changes of BRIP1 expression in transcription level in pan-cancers (ONCOMINE database) | | | | | |
| --- | --- | --- | --- | --- | --- |
| Cancer Site | Types of Cancer vs Normal | Fold change | T-test | P-value | Source and/or Reference |
| Brain | Glioblastoma | 2.243 | 6.968 | 5.63E-10 | Sun Brain ^5^ |
| Brain | Glioblastoma | -3.863 | -7.681 | 4.79E-8 | [Lee Brain](https://www.oncomine.org/resource/ui/component/dataset.html?component=d:21673836) ^6^ |
| Breast | Male Breast Carcinoma | 1.663 | 6.161 | 1.35E-04 | TCGA |
|  | Male Breast Carcinoma | 5.322 | 11.466 | 1.01E-06 | TCGA |
|  | Mixed Lobular and Ductal Breast Carcinoma | 2.295 | 4.771 | 1.37E-04 | TCGA |
|  | Invasive Ductal Breast Carcinoma | 3.285 | 12.457 | 5.80E-21 | TCGA |
|  | Invasive Breast Carcinoma | 2.559 | 7.715 | 1.32E-12 | TCGA |
|  | Invasive Ductal and Lobular Carcinoma | 2.294 | 4.986 | 0.004 | TCGA |
|  | Invasive Lobular Breast Carcinoma | 2.223 | 5.768 | 5.12E-08 | TCGA |
|  | Ductal Breast Carcinoma | 2.096 | 5.482 | 1.11E-06 | Richardson Breast 2 ^7^ |
|  | Invasive Breast Carcinoma Stroma | 3.690 | 14.445 | 1.40E-14 | Finak Breast ^8^ |
| Cervical | Cervical Squamous Cell Carcinoma | 2.306 | 4.059 | 1.22E-04 | Scotto Cervix 2 ^9^ |
| Colorectal | Rectal Mucinous Adenocarcinoma | 2.288 | 8.206 | 8.19E-07 | TCGA |
|  | Colon Mucinous Adenocarcinoma | 2.769 | 7.678 | 1.08E-09 | TCGA |
|  | Cecum Adenocarcinoma | 2.582 | 6.945 | 1.24E-08 | TCGA |
|  | Rectal Adenocarcinoma | 2.103 | 8.317 | 8.49E-12 | TCGA |
| Gastric | Gastric Cancer | 1.828 | 6.104 | 3.90E-09 | Cui Gastric ^10^ |
| Head-Neck | Nasopharyngeal Carcinoma | 1.674 | 6.648 | 9.48E-08 | Sengupta Head-Neck ^11^ |
|  | Oral Cavity Squamous Cell Carcinoma | 1.642 | 5.529 | 5.38E-07 | Peng Head-Neck ^12^ |
| Sarcoma | Leiomyosarcoma | 10.721 | 9.256 | 9.02E-09 | Detwiller Sarcoma ^13^ |
|  | Round Cell Liposarcoma | 5.818 | 7.406 | 5.51E-07 | Detwiller Sarcoma ^13^ |
|  | Fibrosarcoma | 5.921 | 6.097 | 4.00E-06 | Detwiller Sarcoma ^13^ |
| Pancreas | Pancreatic Ductal Adenocarcinoma | 1.882 | 6.818 | 9.38E-06 | Buchholz Pancreas ^14^ |
| Other | Testicular Yolk Sac Tumor | -4.503 | -19.742 | 3.11E-6 | Skotheim Testis ^15^ |
|  | Testicular Embryonal Carcinoma | -4.464 | -16.454 | 1.89E-6 | Skotheim Testis ^15^ |
|  | Testicular Teratoma | -4.282 | -8.926 | 7.59E-4 | Skotheim Testis ^15^ |
|  | Testicular Seminoma | -4.211 | -11.297 | 0.001 | Skotheim Testis ^15^ |

| Table S2. Univariate and Multivariate COX regression for clinical characteristics and BRIP1 expression in various tumors | | | | | | | |
| --- | --- | --- | --- | --- | --- | --- | --- |
| Types | Characteristics | | Total  (N) | Univariate analysis | | Multivariate analysis | |
|  |  |  |  | Hazard ratio (95% CI) | P-value | Hazard ratio (95% CI) | P-value |
| KIRP | Pathologic stages | T3+T4/T1+T2 | 286 | 5.121 (2.790-9.397) | **<0.001** | 1.123 (0.221-5.710) | 0.889 |
|  |  | N1+N2/N0 | 77 | 5.003 (2.062-12.140) | **<0.001** | 1.169 (0.208-6.559) | 0.859 |
|  |  | M1/M0 | 104 | 114.966 (22.481-587.925) | **<0.001** | 72.261 (6.147-849.476) | **<0.001** |
|  | Gender | Male/Female | 288 | 0.638 (0.331-1.230) | 0.18 |  |  |
|  |  |  |  |  |  |  |  |
|  | Age | >60/<=60 | 286 | 0.944 (0.519-1.718) | 0.851 |  |  |
|  | **BRIP1** | **High/Low** | 288 | 2.376 (1.270-4.444) | **0.007** | 2.480 (0.693-8.866) | 0.162 |
| ACC | Pathologic stages | T3+T4/T1+T2 | 77 | 10.286 (3.976-26.608) | **<0.001** | 7.526 (2.514-22.525) | **<0.001** |
|  |  | N1/N0 | 77 | 2.038 (0.769-5.400) | 0.152 |  |  |
|  |  | M1/M0 | 77 | 6.150 (2.710-13.959) | **<0.001** | 1.773 (0.704-4.462) | 0.224 |
|  | Gender | Male/Female | 79 | 1.001 (0.469-2.137) | 0.999 |  |  |
|  | Age | >50/<=50 | 79 | 1.799 (0.846-3.824) | 0.127 |  |  |
|  | **BRIP1** | **High/Low** | 79 | 2.933 (1.318-6.529) | **0.008** | 2.620 (1.133-6.057) | **0.024** |
|  |  |  |  |  |  |  |  |
| LGG | WHO grade | G3/G2 | 466 | 3.059 (2.046-4.573) | **<0.001** | 2.452 (1.587-3.788) | **<0.001** |
|  | Histological type | OLAM+OLDM/ AM | 527 | 0.606 (0.430-0.853) | **0.004** | 0.699 (0.475-1.029) | 0.069 |
|  | Gender | Male/Female | 527 | 1.124 (0.800-1.580) | 0.499 |  |  |
|  | Age | >40/<=40 | 527 | 2.889 (2.009-4.155) | **<0.001** | 2.738 (1.827-4.102) | **<0.001** |
|  | **BRIP1** | **High/Low** | 527 | 2.224 (1.539-3.214) | **<0.001** | 1.674 (1.095-2.559) | **0.017** |
| COAD | Pathologic stages | T3+T4/T1+T2 | 476 | 3.072 (1.423-6.631) | **0.004** | 3.988 (1.227-12.958) | **0.021** |
|  |  | N1+N2/N0 | 477 | 2.592 (1.743-3.855) | **<0.001** | 1.513 (0.903-2.534) | 0.116 |
|  |  | M1/M0 | 414 | 4.193 (2.683-6.554) | **<0.001** | 2.805 (1.652-4.761) | **<0.001** |
|  | Gender | Male/Female | 477 | 1.101 (0.746-1.625) | 0.627 |  |  |
|  | Age | >65/<=65 | 477 | 1.610 (1.052-2.463) | **0.028** | 2.038 (1.257-3.303) | **0.004** |
|  | **BRIP1** | **High/Low** | 477 | 0.461 (0.308-0.691) | **<0.001** | 0.603 (0.385-0.945) | **0.027** |
| READ | Pathologic stages | T3+T4/T1+T2 | 164 | 1.408 (0.476-4.159) | 0.536 |  |  |
|  |  | N1+N2/N0 | 162 | 3.021 (1.244-7.336) | **0.015** | 2.614 (0.913-7.488) | 0.073 |
|  |  | M1/M0 | 149 | 3.412 (1.424-8.174) | **0.006** | 2.255 (0.846-6.008) | 0.104 |
|  | Gender | Male/Female | 166 | 0.916 (0.422-1.988) | 0.824 |  |  |
|  | Age | >65/<=65 | 166 | 3.843 (1.535-9.622) | **0.004** | 5.268 (1.764-15.729) | **0.003** |
|  | **BRIP1** | **High/Low** | 166 | 0.387 (0.172-0.874) | **0.022** | 0.540 (0.225-1.292) | 0.166 |
| STAD | Pathologic stages | T3+T4/T1+T2 | 362 | 1.719 (1.131-2.612) | **0.011** | 1.432 (0.907-2.262) | 0.123 |
|  |  | N1+N2+N3/N0 | 352 | 1.925 (1.264-2.931) | **0.002** | 1.715 (1.092-2.694) | **0.019** |
|  |  | M1/M0 | 352 | 2.254 (1.295-3.924) | **0.004** | 2.324 (1.296-4.166) | **0.005** |
|  | Gender | Male/Female | 370 | 1.267 (0.891-1.804) | 0.188 |  |  |
|  | Age | >65/<=65 | 367 | 1.620 (1.154-2.276) | **0.005** | 1.941 (1.345-2.801) | **<0.001** |
|  | **BRIP1** | **High/Low** | 370 | 0.707 (0.508-0.982) | **0.039** | 0.664 (0.469-0.941) | **0.021** |
| THYM | Masaoka stage | III+IV/I+II | 116 | 1.695 (0.348-8.268) | 0.514 |  |  |
|  | Gender | Male/Female | 118 | 0.629 (0.168-2.356) | 0.492 |  |  |
|  | Age | >60/<=60 | 118 | 4.117 (0.850-19.947) | 0.079 | 3.754 (0.673-20.949) | 0.132 |
|  | Histological type | Type B+ C/Type A | 118 | 0.931 (0.218-3.974) | 0.924 |  |  |
|  | Tumor site | Thymus/Anterior Mediastinum | 118 | 1.043 (0.216-5.027) | 0.958 |  |  |
|  | **BRIP1** | **High/Low** | 118 | 0.056 (0.007-0.483) | **0.009** | 0.052 (0.005-0.512) | **0.011** |
| LUAD | Pathologic stages | T3+T4/T1+T2 | 523 | 2.317 (1.591-3.375) | **<0.001** | 2.054 (1.355-3.112) | **<0.001** |
|  |  | N1+N2+N3/N0 | 510 | 2.601 (1.944-3.480) | **<0.001** | 2.339 (1.671-3.272) | **<0.001** |
|  |  | M1/M0 | 377 | 2.136 (1.248-3.653) | **0.006** | 1.554 (0.868-2.783) | 0.138 |
|  |  | Male/Female | 526 | 1.070 (0.803-1.426) | 0.642 |  |  |
|  | Age | >65/<=65 | 516 | 1.223 (0.916-1.635) | 0.172 |  |  |
|  | BRIP1 | High/Low | 526 | 1.302 (0.977-1.735) | 0.072 | 1.296 (0.930-1.805) | 0.125 |
| MESO | Pathologic stages | T3+T4/T1+T2 | 83 | 0.955 (0.590-1.547) | 0.852 |  |  |
|  |  | N2+N3/N0+N1 | 81 | 0.901 (0.539-1.505) | 0.69 |  |  |
|  |  | M1/M0 | 59 | 1.917 (0.454-8.089) | 0.376 |  |  |
|  | Age | >65/<=65 | 85 | 1.296 (0.805-2.085) | 0.286 |  |  |
|  | Gender | Male/Female | 85 | 0.944 (0.516-1.726) | 0.85 |  |  |
|  | Histological type | SM+BS+DM/EP | 85 | 1.750 (1.074-2.849) | **0.025** | 1.558 (0.950-2.553) | 0.079 |
|  | **BRIP1** | **High/Low** | 85 | 3.393 (2.011-5.725) | **<0.001** | 3.270 (1.927-5.549) | **<0.001** |
| PAAD | Pathologic stages | T3+T4/T1+T2 | 176 | 2.023 (1.072-3.816) | **0.03** | 1.175 (0.606-2.281) | 0.633 |
|  |  | N1/N0 | 173 | 2.154 (1.282-3.618) | **0.004** | 1.975 (1.150-3.391) | **0.014** |
|  |  | M1/M0 | 84 | 0.756 (0.181-3.157) | 0.701 |  |  |
|  | Gender | Male/Female | 178 | 0.809 (0.537-1.219) | 0.311 |  |  |
|  | Age | >65/<=65 | 178 | 1.290 (0.854-1.948) | 0.227 |  |  |
|  | **BRIP1** | **High/Low** | 178 | 1.676 (1.104-2.544) | **0.015** | 1.562 (1.022-2.387) | **0.04** |
| OLAM: Oligoastrocytoma, OLDM: Oligodendroglioma, AM: Astrocytoma, SM: Sarcomatoid, BS: Biphasic, DM: Diffuse malignant, EP: Epithelioid | | | | | | | |

| Table S3. Enrichment analysis of BRIP1-related genes across pancancer | | | | | | | |
| --- | --- | --- | --- | --- | --- | --- | --- |
| ONTOLOGY | ID | Description | GeneRatio | BgRatio | P-value | Q-value | Count |
| BP | GO:0006260 | DNA replication | 40/135 | 274/18670 | 2.38E-41 | 2.03E-38 | 40 |
| BP | GO:0006261 | DNA-dependent DNA replication | 28/135 | 153/18670 | 9.97E-32 | 4.26E-29 | 28 |
| BP | GO:0071103 | DNA conformation change | 35/135 | 327/18670 | 3.1E-31 | 8.82E-29 | 35 |
| BP | GO:0032508 | DNA duplex unwinding | 22/135 | 110/18670 | 6.04E-26 | 1.29E-23 | 22 |
| BP | GO:0006302 | double-strand break repair | 28/135 | 248/18670 | 1.25E-25 | 2.14E-23 | 28 |
| CC | GO:0098687 | chromosomal region | 33/137 | 349/19717 | 3.59E-28 | 3.55E-26 | 33 |
| CC | GO:0055029 | nuclear DNA-directed RNA polymerase complex | 21/137 | 107/19717 | 5.86E-25 | 2.38E-23 | 21 |
| CC | GO:0000428 | DNA-directed RNA polymerase... | 21/137 | 108/19717 | 7.23E-25 | 2.38E-23 | 21 |
| CC | GO:0030880 | RNA polymerase complex | 21/137 | 112/19717 | 1.64E-24 | 4.06E-23 | 21 |
| CC | GO:0016591 | RNA polymerase II, holoenzyme | 18/137 | 83/19717 | 2.3E-22 | 4.55E-21 | 18 |
| MF | GO:0016887 | ATPase activity | 29/134 | 384/17697 | 5.23E-21 | 7.87E-19 | 29 |
| MF | GO:0003697 | single-stranded DNA binding | 19/134 | 113/17697 | 1.26E-20 | 9.5E-19 | 19 |
| MF | GO:0008094 | DNA-dependent ATPase activity | 15/134 | 69/17697 | 2.77E-18 | 1.39E-16 | 15 |
| MF | GO:0140097 | catalytic activity, acting ... | 21/134 | 213/17697 | 9.61E-18 | 3.62E-16 | 21 |
| MF | GO:0003678 | DNA helicase activity | 15/134 | 81/17697 | 3.69E-17 | 1.11E-15 | 15 |
| KEGG | hsa03022 | Basal transcription factors | 13/68 | 45/8076 | 1.89E-17 | 7.36E-16 | 13 |
| KEGG | hsa03420 | Nucleotide excision repair | 12/68 | 47/8076 | 1.91E-15 | 3.71E-14 | 12 |
| KEGG | hsa03030 | DNA replication | 11/68 | 36/8076 | 3.3E-15 | 4.29E-14 | 11 |
| KEGG | hsa03460 | Fanconi anemia pathway | 11/68 | 54/8076 | 4.68E-13 | 4.55E-12 | 11 |
| KEGG | hsa03440 | Homologous recombination | 10/68 | 41/8076 | 8.22E-13 | 6.41E-12 | 10 |
| KEGG:　Kyoto Encyclopedia of Genes and Genomes; GO: Gene Oncology; MF: molecular functions; CC: cellular components; BP: biological processes | | | | | | | |

| Table S4. Association between BRIP1 DNA methylation and gene expression for the 24 tumors of TCGA | | | | | | | |
| --- | --- | --- | --- | --- | --- | --- | --- |
| Type | Probe | | | CpG | | Pearson correlation | |
|  | ID | Start | End | Location | Count | R-value | P-value |
| BLCA | cg05377417 | 61863531 | 61863580 | 61863532 | 4 | -0.212 | **<0.001** |
|  | cg00515161 | 61863539 | 61863538 | 61863540 | 4 | -0.191 | **<0.001** |
|  | cg05986149 | 61863695 | 61863744 | 61863695 | 1 | -0.1 | **<0.05** |
| BRCA | cg15549767 | 61682738 | 61682728 | 61682738 | 0 | 0.21 | **<0.001** |
|  | cg14757157 | 61834395 | 61834444 | 61834395 | 0 | 0.185 | **<0.001** |
|  | cg05377417 | 61863531 | 61863580 | 61863532 | 4 | -0.213 | **<0.001** |
|  | cg00515161 | 61863539 | 61863538 | 61863540 | 4 | -0.271 | **<0.001** |
| CESC | cg11872651 | 61863037 | 61863086 | 61863086 | 2 | -0.152 | **<0.001** |
|  | cg05377417 | 61863531 | 61863580 | 61863532 | 4 | -0.144 | **<0.05** |
|  | cg00515161 | 61863539 | 61863538 | 61863540 | 4 | -0.181 | **<0.01** |
|  | cg05986149 | 61863695 | 61863744 | 61863695 | 1 | -0.144 | **<0.05** |
|  | cg06257227 | 61863772 | 61863821 | 61863772 | 1 | -0.156 | **<0.01** |
| COAD | cg15549767 | 61682738 | 61682728 | 61682738 | 0 | 0.167 | **<0.01** |
|  | cg14757157 | 61834395 | 61834444 | 61834395 | 0 | 0.170 | **<0.01** |
|  | cg05377417 | 61863531 | 61863580 | 61863532 | 4 | -0.122 | **<0.05** |
|  | cg00515161 | 61863539 | 61863538 | 61863540 | 4 | -0.136 | **<0.05** |
| DLBC | cg15549767 | 61682738 | 61682728 | 61682738 | 0 | -0.312 | **<0.05** |
|  | cg05986149 | 61863695 | 61863744 | 61863695 | 1 | -0.314 | **<0.05** |
| GBM | cg15549767 | 61682738 | 61682728 | 61682738 | 0 | 0.285 | **<0.05** |
|  | cg11529614 | 61863690 | 61863739 | 61863690 | 3 | 0.254 | **<0.05** |
| HNSC | cg00515161 | 61863539 | 61863538 | 61863540 | 4 | -0.086 | **<0.05** |
|  | cg06257227 | 61863772 | 61863821 | 61863772 | 1 | 0.094 | **<0.05** |
| LAML | cg11529614 | 61863690 | 61863739 | 61863690 | 3 | -0.201 | **<0.05** |
| LGG | cg12435611 | 61863692 | 61863741 | 61863692 | 2 | 0.124 | **<0.01** |
| LIHC | cg11872651 | 61863037 | 61863086 | 61863086 | 2 | -0.109 | **<0.05** |
|  | cg05377417 | 61863531 | 61863580 | 61863532 | 4 | -0.172 | **<0.001** |
|  | cg00515161 | 61863539 | 61863538 | 61863540 | 4 | -0.155 | **<0.01** |
|  | cg06257227 | 61863772 | 61863821 | 61863772 | 1 | -0.098 | **<0.05** |
| LUAD | cg05377417 | 61863531 | 61863580 | 61863532 | 4 | -0.097 | **<0.05** |
| LUSC | cg05377417 | 61863531 | 61863580 | 61863532 | 4 | -0.171 | **<0.001** |
|  | cg00515161 | 61863539 | 61863538 | 61863540 | 4 | -0.194 | **<0.001** |
| OV | cg12435611 | 61863692 | 61863741 | 61863692 | 2 | -0.142 | **<0.01** |
|  | cg05986149 | 61863695 | 61863744 | 61863695 | 1 | -0.161 | **<0.01** |
| PCPG | cg00869632 | 61863349 | 61863398 | 61863349 | 2 | -0.153 | **<0.05** |
| PRAD | cg15549767 | 61682738 | 61682728 | 61682738 | 0 | 0.101 | **<0.05** |
|  | cg05377417 | 61863531 | 61863580 | 61863532 | 4 | -0.090 | **<0.05** |
|  | cg00515161 | 61863539 | 61863538 | 61863540 | 4 | -0.12 | **<0.05** |
|  | cg12435611 | 61863692 | 61863741 | 61863692 | 2 | 0.108 | **<0.05** |
| READ | cg11529614 | 61863690 | 61863739 | 61863690 | 3 | 0.208 | **<0.05** |
| SARC | cg15549767 | 61682738 | 61682728 | 61682738 | 0 | 0.181 | **<0.01** |
|  | cg14757157 | 61834395 | 61834444 | 61834395 | 0 | 0.159 | **<0.01** |
|  | cg05377417 | 61863531 | 61863580 | 61863532 | 4 | -0.233 | **<0.001** |
|  | cg00515161 | 61863539 | 61863538 | 61863540 | 4 | -0.241 | **<0.001** |
| SKCM | cg15549767 | 61682738 | 61682728 | 61682738 | 0 | 0.192 | **<0.001** |
|  | cg11872651 | 61863037 | 61863086 | 61863086 | 2 | -0.108 | **<0.05** |
|  | cg00869632 | 61863349 | 61863398 | 61863349 | 2 | -0.099 | **<0.05** |
|  | cg05377417 | 61863531 | 61863580 | 61863532 | 4 | -0.236 | **<0.001** |
|  | cg00515161 | 61863539 | 61863538 | 61863540 | 4 | -0.239 | **<0.001** |
| STAD | cg00869632 | 61863349 | 61863398 | 61863349 | 2 | -0.142 | **<0.01** |
| TGCT | cg15549767 | 61682738 | 61682728 | 61682738 | 0 | -0.475 | **<0.001** |
|  | cg14757157 | 61834395 | 61834444 | 61834395 | 0 | -0.469 | **<0.001** |
| THCA | cg15549767 | 61682738 | 61682728 | 61682738 | 0 | 0.084 | **<0.05** |
|  | cg14757157 | 61834395 | 61834444 | 61834395 | 0 | 0.108 | **<0.05** |
|  | cg00869632 | 61863349 | 61863398 | 61863349 | 2 | -0.184 | **<0.001** |
| THYM | cg14757157 | 61834395 | 61834444 | 61834395 | 0 | 0.273 | **<0.01** |
|  | cg11872651 | 61863037 | 61863086 | 61863086 | 2 | -0.385 | **<0.001** |
|  | cg00869632 | 61863349 | 61863398 | 61863349 | 2 | -0.343 | **<0.001** |
|  | cg05986149 | 61863695 | 61863744 | 61863695 | 1 | -0.298 | **<0.001** |
|  | cg06257227 | 61863772 | 61863821 | 61863772 | 1 | -0.241 | **<0.01** |
| UCEC | cg11872651 | 61863037 | 61863086 | 61863086 | 2 | -0.101 | **<0.05** |
|  | cg00869632 | 61863349 | 61863398 | 61863349 | 2 | -0.124 | **<0.01** |
|  | cg05377417 | 61863531 | 61863580 | 61863532 | 4 | -0.110 | **<0.05** |
|  | cg00515161 | 61863539 | 61863538 | 61863540 | 4 | -0.101 | **<0.05** |
|  | cg05986149 | 61863695 | 61863744 | 61863695 | 1 | -0.106 | **<0.05** |
|  | cg06257227 | 61863772 | 61863821 | 61863772 | 1 | -0.099 | **<0.05** |
| UCS | cg15549767 | 61682738 | 61682728 | 61682738 | 0 | 0.370 | **<0.01** |
|  | cg14757157 | 61834395 | 61834444 | 61834395 | 0 | 0.330 | **<0.05** |

| Table S5. The correlation between BRIP1 and other genes of ACC from the TCGA database | | | | | | |
| --- | --- | --- | --- | --- | --- | --- |
| Genes | Chr^*^ | Start^a^ | End^b^ | -Log10 (p) | Correlation | Samples |
| CENPE | 4 | 104026963 | 104119566 | 14.3 | 0.74 | 78 |
| XRCC2 | 7 | 152341864 | 152373250 | 14.2 | 0.74 | 78 |
| ZNF367 | 9 | 99148223 | 99180611 | 12.5 | 0.71 | 78 |
| RAD51AP1 | 12 | 4647950 | 4669214 | 12.3 | 0.71 | 78 |
| POLQ | 3 | 121150278 | 121264853 | 11.8 | 0.7 | 78 |
| CCDC99 | 5 | 169010638 | 169031781 | 11.8 | 0.69 | 78 |
| ESCO2 | 8 | 27629466 | 27670157 | 11.7 | 0.69 | 78 |
| CENPK | 5 | 64813593 | 64858998 | 11.7 | 0.69 | 78 |
| SMC2 | 9 | 106856541 | 106903698 | 11.3 | 0.68 | 78 |
| KIF11 | 10 | 94353043 | 94415150 | 11.2 | 0.68 | 78 |
| TIMELESS | 12 | 56810903 | 56843187 | 11.1 | 0.68 | 78 |
| CASC5 | 15 | 40886218 | 40956540 | 11.1 | 0.68 | 78 |
| FOXM1 | 12 | 2966847 | 2986206 | 11 | 0.68 | 78 |
| KIF20B | 10 | 91461367 | 91534700 | 10.9 | 0.67 | 78 |
| CLSPN | 1 | 36185819 | 36235568 | 10.9 | 0.67 | 78 |
| E2F7 | 12 | 77415027 | 77459360 | 10.8 | 0.67 | 78 |
| KIAA1524 | 3 | 108268716 | 108308491 | 10.8 | 0.67 | 78 |
| EXO1 | 1 | 242011269 | 242058450 | 10.6 | 0.67 | 78 |
| KNTC1 | 12 | 123011793 | 123110943 | 10.6 | 0.67 | 78 |
| RBL1 | 20 | 35624752 | 35724398 | 10.5 | 0.67 | 78 |
| FAM83D | 20 | 37554955 | 37581703 | 10.5 | 0.67 | 78 |
| MAD2L1 | 4 | 120976763 | 120988229 | 10.4 | 0.66 | 78 |
| CHAF1B | 21 | 37757676 | 37791313 | 10.4 | 0.66 | 78 |
| RFC5 | 12 | 118451393 | 118470935 | 10.3 | 0.66 | 78 |
| FAM111B | 11 | 58874658 | 58894883 | 10.3 | 0.66 | 78 |
| CENPI | X | 100353178 | 100418670 | 10.3 | 0.66 | 78 |
| TOP2A | 17 | 38544768 | 38574202 | 10.3 | 0.66 | 78 |
| INCENP | 11 | 61891445 | 61920635 | 10.2 | 0.66 | 78 |
| KIF14 | 1 | 200520628 | 200589862 | 10.1 | 0.66 | 78 |
| TPX2 | 20 | 30327074 | 30389608 | 10 | 0.65 | 78 |
| BRCA2 | 13 | 32889611 | 32973805 | 10.1 | 0.66 | 78 |
| BRCA1 | 17 | 41196312 | 41277500 | 10.6 | 0.67 | 78 |
| BARD1 | 2 | 215590370 | 215674428 | 12.1 | 0.7 | 78 |
| ATAD5 | 17 | 29158988 | 29222887 | 10.5 | 0.66 | 78 |
| ATAD2 | 8 | 124332090 | 124428590 | 12 | 0.7 | 78 |
| ANLN | 7 | 36429415 | 36493400 | 11.6 | 0.69 | 78 |
| ACC: Adrenocortical Carcinoma, *: Chromosome, a: chromosome starting site of the gene, b: chromosome ending site of the gene. | | | | | | |

| Table S6. The correlation between BRIP1 and other genes of BLCA from the TCGA database | | | | | | |
| --- | --- | --- | --- | --- | --- | --- |
| Genes | Chr^*^ | Start^a^ | End^b^ | -Log10 (p) | Correlation | Samples |
| CLSPN | 1 | 36197713 | 36235551 | 32.1 | 0.797 | 144 |
| PRR11 | 17 | 57232860 | 57284070 | 29.6 | 0.777 | 144 |
| RBL1 | 20 | 35626178 | 35724410 | 28.1 | 0.764 | 144 |
| STIL | 1 | 47715811 | 47779819 | 27 | 0.754 | 144 |
| DNMT1 | 19 | 10244022 | 10305755 | 25.9 | 0.744 | 144 |
| E2F7 | 12 | 77415026 | 77459360 | 25.8 | 0.743 | 144 |
| MCM10 | 10 | 13203554 | 13253104 | 25.4 | 0.739 | 144 |
| RAD51AP1 | 12 | 4647950 | 4669213 | 25.2 | 0.737 | 144 |
| CCNE2 | 8 | 95892453 | 95907482 | 25.2 | 0.737 | 144 |
| CENPI | X | 100353178 | 100417978 | 25.1 | 0.736 | 144 |
| WDHD1 | 14 | 55405656 | 55493819 | 24.7 | 0.732 | 144 |
| ORC1 | 1 | 52838501 | 52870143 | 24.4 | 0.729 | 144 |
| C15ORF42 | 15 | 90118818 | 90171253 | 24.2 | 0.727 | 144 |
| KIF14 | 1 | 200520625 | 200589862 | 24.2 | 0.727 | 144 |
| ECT2 | 3 | 172468532 | 172539263 | 24.2 | 0.727 | 144 |
| CASC5 | 15 | 40886447 | 40954881 | 24 | 0.725 | 144 |
| KNTC1 | 12 | 123011809 | 123110947 | 23.9 | 0.724 | 144 |
| KIF18A | 11 | 28042163 | 28129746 | 23.9 | 0.724 | 144 |
| DTL | 1 | 212208919 | 212278187 | 23.9 | 0.723 | 144 |
| TPX2 | 20 | 30326904 | 30389603 | 23.9 | 0.724 | 144 |
| PLK4 | 4 | 128802016 | 128820377 | 23.8 | 0.723 | 144 |
| BUB1B | 15 | 40453210 | 40513337 | 23.6 | 0.721 | 144 |
| UHRF1 | 19 | 4909510 | 4962165 | 23.5 | 0.72 | 144 |
| MELK | 9 | 36572905 | 36677679 | 22.8 | 0.711 | 144 |
| MMS22L | 6 | 97590037 | 97731061 | 22.6 | 0.71 | 144 |
| KIF24 | 9 | 34211974 | 34329198 | 22.6 | 0.71 | 144 |
| KIF4A | X | 69509879 | 69640774 | 22.5 | 0.709 | 144 |
| RACGAP1 | 12 | 50382945 | 50419307 | 22.3 | 0.706 | 144 |
| DNA2 | 10 | 70173821 | 70231879 | 22.3 | 0.706 | 144 |
| CENPE | 4 | 104026963 | 104119566 | 22.2 | 0.705 | 144 |
| DEPDC1 | 1 | 68939835 | 68962799 | 22.1 | 0.704 | 144 |
| TOP2A | 17 | 38544773 | 38574202 | 22 | 0.702 | 144 |
| SGOL2 | 2 | 201390865 | 201448818 | 22 | 0.702 | 144 |
| SMC2 | 9 | 106856541 | 106903700 | 21.9 | 0.701 | 144 |
| PRC1 | 15 | 91509268 | 91537804 | 21.9 | 0.702 | 144 |
| NCAPG2 | 7 | 158424003 | 158497520 | 21.9 | 0.701 | 144 |
| MKI67 | 10 | 129894925 | 129924468 | 21.9 | 0.701 | 144 |
| CCNA2 | 4 | 122737599 | 122745088 | 21.9 | 0.701 | 144 |
| XRCC2 | 7 | 152343587 | 152373250 | 21.8 | 0.7 | 144 |
| CHAF1B | 21 | 37757689 | 37789125 | 21.7 | 0.699 | 144 |
| EXO1 | 1 | 242011493 | 242053241 | 21.6 | 0.697 | 144 |
| TIMELESS | 12 | 56810157 | 56843200 | 21.5 | 0.697 | 144 |
| PRIM1 | 12 | 57125364 | 57146146 | 21.5 | 0.696 | 144 |
| FBXO5 | 6 | 153291658 | 153304740 | 21.5 | 0.697 | 144 |
| NUSAP1 | 15 | 41624892 | 41673248 | 21.4 | 0.696 | 144 |
| GTSE1 | 22 | 46692638 | 46726707 | 21.3 | 0.694 | 144 |
| KIF18B | 17 | 43002082 | 43025082 | 21.2 | 0.693 | 144 |
| NCAPG | 4 | 17812525 | 17846487 | 21.1 | 0.692 | 144 |
| TMPO | 12 | 98909351 | 98944157 | 21 | 0.691 | 144 |
| NDC80 | 18 | 2571510 | 2616634 | 20.9 | 0.69 | 144 |
| NEIL3 | 4 | 178230991 | 178284092 | 20.9 | 0.689 | 144 |
| GSG2 | 17 | 3627197 | 3629992 | 20.8 | 0.688 | 144 |
| FOXM1 | 12 | 2966847 | 2986321 | 20.7 | 0.687 | 144 |
| FAM111B | 11 | 58874658 | 58894888 | 20.6 | 0.686 | 144 |
| CDCA5 | 11 | 64844927 | 64851615 | 20.6 | 0.686 | 144 |
| KIF23 | 15 | 69706627 | 69740764 | 20.6 | 0.685 | 144 |
| KIAA1524 | 3 | 108268718 | 108308491 | 20.5 | 0.685 | 144 |
| GINS1 | 20 | 25388323 | 25429191 | 20.3 | 0.682 | 144 |
| TCF19 | 6 | 31126303 | 31131992 | 20.2 | 0.681 | 144 |
| RFC3 | 13 | 34392206 | 34540695 | 20.2 | 0.681 | 144 |
| MCM2 | 3 | 127317253 | 127341278 | 20.1 | 0.679 | 144 |
| DLGAP5 | 14 | 55614834 | 55658396 | 20.1 | 0.68 | 144 |
| MCM8 | 20 | 5931298 | 5975831 | 20 | 0.678 | 144 |
| KIF11 | 10 | 94352825 | 94415152 | 20 | 0.679 | 144 |
| FEN1 | 11 | 61560109 | 61564714 | 20 | 0.678 | 144 |
| HELLS | 10 | 96305543 | 96361856 | 19.9 | 0.677 | 144 |
| DCLRE1B | 1 | 114447915 | 114456708 | 19.8 | 0.676 | 144 |
| MAD2L1 | 4 | 120980579 | 120988013 | 19.7 | 0.674 | 144 |
| LMNB1 | 5 | 126112315 | 126172712 | 19.7 | 0.674 | 144 |
| FANCB | X | 14861529 | 14891184 | 19.7 | 0.675 | 144 |
| SPC24 | 19 | 11257831 | 11266484 | 19.6 | 0.673 | 144 |
| MTBP | 8 | 121457666 | 121535875 | 19.6 | 0.673 | 144 |
| KIF20B | 10 | 91461367 | 91534700 | 19.6 | 0.672 | 144 |
| CDC7 | 1 | 91966404 | 91991321 | 19.6 | 0.673 | 144 |
| CDCA8 | 1 | 38158159 | 38175391 | 19.3 | 0.669 | 144 |
| CDK1 | 10 | 62538089 | 62554610 | 19.1 | 0.666 | 144 |
| CKAP2L | 2 | 113495444 | 113522254 | 19.1 | 0.667 | 144 |
| FANCI | 15 | 89787194 | 89860362 | 19 | 0.665 | 144 |
| ESCO2 | 8 | 27632058 | 27670141 | 19 | 0.665 | 144 |
| NUP155 | 5 | 37291941 | 37371197 | 18.9 | 0.664 | 144 |
| CDC6 | 17 | 38444146 | 38459413 | 18.9 | 0.663 | 144 |
| FANCC | 9 | 97861336 | 98079991 | 18.8 | 0.662 | 144 |
| C4ORF46 | 4 | 159587831 | 159593407 | 18.8 | 0.662 | 144 |
| MIS18BP1 | 14 | 45672393 | 45722605 | 18.7 | 0.661 | 144 |
| LIN9 | 1 | 226418861 | 226497556 | 18.7 | 0.661 | 144 |
| RFC4 | 3 | 186507682 | 186524484 | 18.6 | 0.659 | 144 |
| KPNA2 | 17 | 66031848 | 66042970 | 18.6 | 0.659 | 144 |
| DEPDC1B | 5 | 59892739 | 59995993 | 18.6 | 0.66 | 144 |
| EZH2 | 7 | 148504464 | 148581441 | 18.5 | 0.658 | 144 |
| TOPBP1 | 3 | 133319449 | 133380737 | 18.5 | 0.657 | 144 |
| RAD54L | 1 | 46713367 | 46744145 | 18.5 | 0.658 | 144 |
| POLE2 | 14 | 50110270 | 50155098 | 18.5 | 0.657 | 144 |
| KIFC1 | 6 | 33359313 | 33377699 | 18.5 | 0.658 | 144 |
| INCENP | 11 | 61891445 | 61920635 | 18.5 | 0.659 | 144 |
| SMC4 | 3 | 160117430 | 160152741 | 18.4 | 0.657 | 144 |
| CIT | 12 | 120123595 | 120315095 | 18.4 | 0.657 | 144 |
| TYMS | 18 | 657604 | 673499 | 18.3 | 0.655 | 144 |
| CHEK1 | 11 | 125495031 | 125546150 | 18.3 | 0.655 | 144 |
| ZWINT | 10 | 58117199 | 58121034 | 18.1 | 0.653 | 144 |
| WDR76 | 15 | 44119112 | 44160617 | 18.1 | 0.652 | 144 |
| TTK | 6 | 80714322 | 80752244 | 18.1 | 0.652 | 144 |
| MYBL2 | 20 | 42295709 | 42345122 | 18 | 0.652 | 144 |
| FANCM | 14 | 45605136 | 45670093 | 18 | 0.65 | 144 |
| CENPK | 5 | 64813593 | 64858995 | 18 | 0.65 | 144 |
| ZNF367 | 9 | 99148225 | 99180669 | 17.8 | 0.649 | 144 |
| CCNB2 | 15 | 59397284 | 59417244 | 17.8 | 0.648 | 144 |
| USP1 | 1 | 62901975 | 62917475 | 17.7 | 0.647 | 144 |
| KIF15 | 3 | 44803209 | 44894748 | 17.6 | 0.645 | 144 |
| WHSC1 | 4 | 1873123 | 1983934 | 17.6 | 0.646 | 144 |
| KIF2C | 1 | 45205490 | 45233438 | 17.6 | 0.645 | 144 |
| CDCA2 | 8 | 25316513 | 25365425 | 17.5 | 0.644 | 144 |
| RRM2 | 2 | 10262695 | 10271546 | 17.5 | 0.643 | 144 |
| NCAPD2 | 12 | 6603298 | 6641132 | 17.5 | 0.645 | 144 |
| SUV39H2 | 10 | 14920782 | 14946304 | 17.4 | 0.643 | 144 |
| SASS6 | 1 | 100549102 | 100598511 | 17.4 | 0.643 | 144 |
| NCAPD3 | 11 | 134022337 | 134094426 | 17.4 | 0.642 | 144 |
| CENPO | 2 | 25016175 | 25045245 | 17.4 | 0.642 | 144 |
| CDKN3 | 14 | 54863673 | 54886934 | 17.4 | 0.642 | 144 |
| SPC25 | 2 | 169727401 | 169746944 | 17.2 | 0.639 | 144 |
| FANCA | 16 | 89803959 | 89883065 | 17.1 | 0.638 | 144 |
| OIP5 | 15 | 41601466 | 41624819 | 17 | 0.637 | 144 |
| NCAPH | 2 | 97001484 | 97041274 | 17 | 0.637 | 144 |
| CENPF | 1 | 214776532 | 214837914 | 17 | 0.637 | 144 |
| FIGNL1 | 7 | 50511832 | 50518088 | 16.9 | 0.635 | 144 |
| DSCC1 | 8 | 120846181 | 120868170 | 16.9 | 0.636 | 144 |
| CEP78 | 9 | 80850991 | 80886799 | 16.9 | 0.635 | 144 |
| CDC25C | 5 | 137620959 | 137674044 | 16.9 | 0.634 | 144 |
| MASTL | 10 | 27443753 | 27475848 | 16.5 | 0.629 | 144 |
| C15ORF23 | 15 | 40674922 | 40686489 | 16.5 | 0.629 | 144 |
| DBF4 | 7 | 87505544 | 87538856 | 16.4 | 0.628 | 144 |
| PLK1 | 16 | 23690201 | 23701688 | 16.3 | 0.626 | 144 |
| MPHOSPH9 | 12 | 123640946 | 123717658 | 16.3 | 0.626 | 144 |
| LRR1 | 14 | 50065415 | 50081390 | 16.3 | 0.625 | 144 |
| SKA3 | 13 | 21727734 | 21750741 | 16.2 | 0.624 | 144 |
| MCM4 | 8 | 48872763 | 48890719 | 16.2 | 0.625 | 144 |
| FANCG | 9 | 35073835 | 35080013 | 16.2 | 0.623 | 144 |
| CENPL | 1 | 173768688 | 173793777 | 16.2 | 0.624 | 144 |
| CCDC99 | 5 | 169010638 | 169031781 | 16 | 0.621 | 144 |
| SKA1 | 18 | 47901392 | 47920538 | 15.9 | 0.619 | 144 |
| CKAP5 | 11 | 46765084 | 46867859 | 15.9 | 0.62 | 144 |
| GLTSCR2 | 19 | 48248793 | 48274141 | 15.8 | -0.618 | 144 |
| ERCC6L | X | 71424507 | 71458858 | 15.8 | 0.618 | 144 |
| CEP152 | 15 | 49030135 | 49103343 | 15.8 | 0.618 | 144 |
| BUB1 | 2 | 111395409 | 111435684 | 15.8 | 0.618 | 144 |
| NUP85 | 17 | 73201597 | 73231854 | 15.8 | 0.618 | 144 |
| MLF1IP | 4 | 185615219 | 185655286 | 15.7 | 0.616 | 144 |
| CENPA | 2 | 27008882 | 27023934 | 15.7 | 0.616 | 144 |
| TEX30 | 13 | 103418463 | 103426149 | 15.6 | 0.615 | 144 |
| RFWD3 | 16 | 74655297 | 74700779 | 15.6 | 0.614 | 144 |
| PRIM2 | 6 | 57182422 | 57513376 | 15.6 | 0.615 | 144 |
| NEK2 | 1 | 211831599 | 211848972 | 15.6 | 0.615 | 144 |
| E2F1 | 20 | 32263292 | 32274210 | 15.5 | 0.612 | 144 |
| DIAPH3 | 13 | 60239723 | 60738119 | 15.5 | 0.613 | 144 |
| POLE | 12 | 133200348 | 133263945 | 15.5 | 0.613 | 144 |
| MCM7 | 7 | 99690404 | 99699427 | 15.5 | 0.613 | 144 |
| MCM6 | 2 | 136597196 | 136634011 | 15.5 | 0.614 | 144 |
| GMPS | 3 | 155588325 | 155655520 | 15.5 | 0.612 | 144 |
| RMI1 | 9 | 86595637 | 86618987 | 15.4 | 0.612 | 144 |
| DEK | 6 | 18224400 | 18264799 | 15.4 | 0.612 | 144 |
| HJURP | 2 | 234745486 | 234763212 | 15.3 | 0.609 | 144 |
| FBXO45 | 3 | 196295725 | 196315930 | 15.3 | 0.61 | 144 |
| CEP55 | 10 | 95256369 | 95288849 | 15.3 | 0.61 | 144 |
| WDR62 | 19 | 36545783 | 36596012 | 15.3 | 0.61 | 144 |
| LMNB2 | 19 | 2428164 | 2456958 | 15.3 | 0.609 | 144 |
| TMEM48 | 1 | 54231134 | 54304225 | 15.2 | 0.608 | 144 |
| IQGAP3 | 1 | 156495197 | 156542396 | 15.2 | 0.608 | 144 |
| SPAG5 | 17 | 26904583 | 26941211 | 15.1 | 0.606 | 144 |
| POLA2 | 11 | 65029432 | 65065088 | 15 | 0.605 | 144 |
| NEDD1 | 12 | 97301001 | 97347469 | 15 | 0.605 | 144 |
| NUP205 | 7 | 135242662 | 135333499 | 14.9 | 0.604 | 144 |
| HMMR | 5 | 162887517 | 162918953 | 14.9 | 0.604 | 144 |
| CEP76 | 18 | 12661955 | 12702703 | 14.8 | 0.601 | 144 |
| POLA1 | X | 24712058 | 25015102 | 14.8 | 0.601 | 144 |
| KIF20A | 5 | 137514417 | 137523404 | 14.8 | 0.601 | 144 |
| POLD1 | 19 | 50887593 | 50921271 | 14.7 | 0.599 | 144 |
| EME1 | 17 | 48450581 | 48458820 | 14.6 | 0.598 | 144 |
| DONSON | 21 | 34947783 | 35288158 | 14.6 | 0.598 | 144 |
| CSE1L | 20 | 47662838 | 47713486 | 14.6 | 0.598 | 144 |
| POLQ | 3 | 121150273 | 121264853 | 14.6 | 0.598 | 144 |
| FAM64A | 17 | 6347735 | 6354385 | 14.5 | 0.596 | 144 |
| NUP62CL | X | 106366657 | 106449670 | 14.4 | 0.595 | 144 |
| GAS2L3 | 12 | 100967489 | 101018685 | 14.4 | 0.595 | 144 |
| XPO5 | 6 | 43490068 | 43543812 | 14.4 | 0.595 | 144 |
| TROAP | 12 | 49716971 | 49725514 | 14.4 | 0.594 | 144 |
| TMEM194A | 12 | 57449426 | 57472574 | 14.3 | 0.593 | 144 |
| PSMC3IP | 17 | 40724329 | 40729747 | 14.3 | 0.593 | 144 |
| GATC | 12 | 120884241 | 120901556 | 14.3 | 0.592 | 144 |
| BRCA2 | 13 | 32889617 | 32973809 | 25.9 | 0.744 | 144 |
| BRCA1 | 17 | 41196312 | 41322420 | 27.7 | 0.76 | 144 |
| BLM | 15 | 91260579 | 91358686 | 16.6 | 0.63 | 144 |
| BIRC5 | 17 | 76210277 | 76221716 | 15.6 | 0.614 | 144 |
| AURKB | 17 | 8108049 | 8113883 | 15 | 0.605 | 144 |
| AURKA | 20 | 54944445 | 54967351 | 18.3 | 0.656 | 144 |
| ATAD5 | 17 | 29159023 | 29222295 | 22.3 | 0.706 | 144 |
| ATAD2 | 8 | 124332091 | 124428590 | 24.4 | 0.729 | 144 |
| ASPM | 1 | 197053257 | 197115824 | 23.5 | 0.719 | 144 |
| ASF1B | 19 | 14230321 | 14247440 | 16.1 | 0.622 | 144 |
| ARHGAP11B | 15 | 30916697 | 30977810 | 25.1 | 0.736 | 144 |
| ARHGAP11A | 15 | 32907345 | 32931868 | 24.9 | 0.734 | 144 |
| ANKRD32 | 5 | 93954391 | 94031573 | 16 | 0.621 | 144 |
| BLCA: Bladder Urothelial Carcinoma, *: Chromosome, a: chromosome starting site of the gene, b: chromosome ending site of the gene. | | | | | | |

| Table S7. The correlation between BRIP1 and other genes of LUSC from the TCGA database | | | | | | |
| --- | --- | --- | --- | --- | --- | --- |
| Genes | Chr^*^ | Start^a^ | End^b^ | -Log10 (p) | Correlation | Samples |
| DTL | 1 | 212208919 | 212278187 | 37.6 | 0.691 | 259 |
| TMEM194A | 12 | 57449426 | 57472574 | 33.4 | 0.661 | 259 |
| FANCI | 15 | 89787194 | 89860362 | 33 | 0.657 | 259 |
| INTS2 | 17 | 59942728 | 60005377 | 31.6 | 0.646 | 259 |
| MED13 | 17 | 60019966 | 60142643 | 31.5 | 0.646 | 259 |
| MSH6 | 2 | 48010221 | 48034092 | 31.4 | 0.645 | 259 |
| E2F7 | 12 | 77415026 | 77459360 | 31.2 | 0.643 | 259 |
| DHX9 | 1 | 182808439 | 182857117 | 30.8 | 0.64 | 259 |
| SPAG5 | 17 | 26904583 | 26941211 | 30.4 | 0.636 | 259 |
| CHAF1A | 19 | 4402660 | 4443394 | 29.3 | 0.627 | 259 |
| TIMELESS | 12 | 56810157 | 56843200 | 28.4 | 0.62 | 259 |
| KIF18B | 17 | 43002082 | 43025082 | 28.3 | 0.618 | 259 |
| MMS22L | 6 | 97590037 | 97731061 | 28 | 0.616 | 259 |
| TLK2 | 17 | 60536019 | 60692841 | 27.7 | 0.613 | 259 |
| KIF15 | 3 | 44803209 | 44894748 | 27.6 | 0.612 | 259 |
| KIF14 | 1 | 200520625 | 200589862 | 27.3 | 0.61 | 259 |
| C15ORF42 | 15 | 90118818 | 90171253 | 27 | 0.607 | 259 |
| RBL1 | 20 | 35626178 | 35724410 | 27 | 0.607 | 259 |
| XRCC2 | 7 | 152343587 | 152373250 | 25.6 | 0.594 | 259 |
| CEP152 | 15 | 49030135 | 49103343 | 25.6 | 0.593 | 259 |
| SENP1 | 12 | 48436757 | 48500091 | 25.5 | 0.593 | 259 |
| KAT7 | 17 | 47865981 | 47906458 | 25.5 | 0.592 | 259 |
| CENPO | 2 | 25016175 | 25045245 | 24.6 | 0.583 | 259 |
| KIF4A | X | 69509879 | 69640774 | 24.5 | 0.582 | 259 |
| POLA1 | X | 24712058 | 25015102 | 24.4 | 0.582 | 259 |
| GNPTG | 16 | 1401900 | 1413352 | 24.4 | -0.581 | 259 |
| SMC2 | 9 | 106856541 | 106903700 | 23.9 | 0.577 | 259 |
| USP13 | 3 | 179370933 | 179507189 | 23.7 | 0.574 | 259 |
| TOP2A | 17 | 38544773 | 38574202 | 23.7 | 0.574 | 259 |
| MCM8 | 20 | 5931298 | 5975831 | 23.5 | 0.572 | 259 |
| RIF1 | 2 | 152266397 | 152364527 | 23.4 | 0.571 | 259 |
| KPNB1 | 17 | 45727275 | 45761004 | 23.4 | 0.571 | 259 |
| ECT2 | 3 | 172468532 | 172539263 | 23.3 | 0.57 | 259 |
| TOPBP1 | 3 | 133319449 | 133380737 | 23.1 | 0.568 | 259 |
| CDC6 | 17 | 38444146 | 38459413 | 23.1 | 0.568 | 259 |
| MCM6 | 2 | 136597196 | 136634011 | 23 | 0.567 | 259 |
| DCAF7 | 17 | 61627822 | 61671642 | 23 | 0.567 | 259 |
| CENPI | X | 100353178 | 100417978 | 22.9 | 0.566 | 259 |
| POLQ | 3 | 121150273 | 121264853 | 22.8 | 0.565 | 259 |
| SMC1A | X | 53401070 | 53449618 | 22.6 | 0.563 | 259 |
| GSG2 | 17 | 3627197 | 3629992 | 22.6 | 0.562 | 259 |
| EME1 | 17 | 48450581 | 48458820 | 22.6 | 0.563 | 259 |
| CBX1 | 17 | 46147414 | 46178883 | 22.6 | 0.562 | 259 |
| CASC5 | 15 | 40886447 | 40954881 | 22.6 | 0.562 | 259 |
| SMC4 | 3 | 160117430 | 160152741 | 22.1 | 0.558 | 259 |
| UHRF1 | 19 | 4909510 | 4962165 | 21.8 | 0.554 | 259 |
| MCM3 | 6 | 52128812 | 52149582 | 21.8 | 0.554 | 259 |
| KIF24 | 9 | 34211974 | 34329198 | 21.7 | 0.553 | 259 |
| NCAPH | 2 | 97001484 | 97041274 | 21.6 | 0.551 | 259 |
| BUB1B | 15 | 40453210 | 40513337 | 21.6 | 0.551 | 259 |
| KIF23 | 15 | 69706627 | 69740764 | 21.5 | 0.55 | 259 |
| POLD4 | 11 | 67119019 | 67121052 | 21.4 | -0.549 | 259 |
| CLSPN | 1 | 36197713 | 36235551 | 21.4 | 0.549 | 259 |
| CEP78 | 9 | 80850991 | 80886799 | 21.4 | 0.549 | 259 |
| CST3 | 20 | 23608534 | 23618574 | 21.3 | -0.548 | 259 |
| CD63 | 12 | 56119230 | 56122910 | 21.3 | -0.548 | 259 |
| TRIM37 | 17 | 57060000 | 57184266 | 21.2 | 0.547 | 259 |
| KNTC1 | 12 | 123011809 | 123110947 | 21 | 0.545 | 259 |
| BUB1 | 2 | 111395409 | 111435684 | 21 | 0.545 | 259 |
| EXO1 | 1 | 242011493 | 242053241 | 20.8 | 0.542 | 259 |
| CDK2 | 12 | 56360556 | 56366568 | 20.8 | 0.543 | 259 |
| ZNF367 | 9 | 99148225 | 99180669 | 20.7 | 0.541 | 259 |
| SUZ12 | 17 | 30264044 | 30328057 | 20.7 | 0.541 | 259 |
| SRSF1 | 17 | 56078280 | 56084707 | 20.7 | 0.541 | 259 |
| FOXM1 | 12 | 2966847 | 2986321 | 20.6 | 0.54 | 259 |
| FANCC | 9 | 97861336 | 98079991 | 20.5 | 0.539 | 259 |
| DNMT1 | 19 | 10244022 | 10305755 | 20.5 | 0.54 | 259 |
| CHAF1B | 21 | 37757689 | 37789125 | 20.5 | 0.539 | 259 |
| CENPF | 1 | 214776532 | 214837914 | 20.5 | 0.539 | 259 |
| CCP110 | 16 | 19535179 | 19564728 | 20.5 | 0.539 | 259 |
| LHPP | 10 | 126150341 | 126302710 | 20.4 | -0.537 | 259 |
| RFWD3 | 16 | 74655297 | 74700779 | 20.3 | 0.537 | 259 |
| KHSRP | 19 | 6413119 | 6424822 | 20.2 | 0.535 | 259 |
| NUSAP1 | 15 | 41624892 | 41673248 | 20 | 0.533 | 259 |
| SGOL1 | 3 | 20202085 | 20227724 | 19.9 | 0.531 | 259 |
| ESPL1 | 12 | 53662083 | 53687427 | 19.8 | 0.531 | 259 |
| CPSF6 | 12 | 69633317 | 69668138 | 19.8 | 0.53 | 259 |
| MTBP | 8 | 121457666 | 121535875 | 19.7 | 0.529 | 259 |
| HELZ | 17 | 65066554 | 65241319 | 19.7 | 0.53 | 259 |
| LMNB1 | 5 | 126112315 | 126172712 | 19.6 | 0.528 | 259 |
| ZNF326 | 1 | 90460678 | 90494094 | 19.4 | 0.526 | 259 |
| XPO1 | 2 | 61705069 | 61765418 | 19.4 | 0.527 | 259 |
| PPM1D | 17 | 58677544 | 58743640 | 19.4 | 0.526 | 259 |
| MTMR4 | 17 | 56566893 | 56595251 | 19.4 | 0.526 | 259 |
| RFX7 | 15 | 56379479 | 56535483 | 19.3 | 0.525 | 259 |
| CDC25A | 3 | 48198668 | 48229801 | 19.3 | 0.525 | 259 |
| FAM161A | 2 | 62051983 | 62081278 | 19.2 | 0.523 | 259 |
| SEPW1 | 19 | 48281842 | 48287943 | 19.1 | -0.522 | 259 |
| RRAS | 19 | 50138552 | 50143400 | 19.1 | -0.523 | 259 |
| FANCD2 | 3 | 10068113 | 10143614 | 19.1 | 0.522 | 259 |
| FANCB | X | 14861529 | 14891184 | 19.1 | 0.522 | 259 |
| CNTLN | 9 | 17135038 | 17503917 | 19 | 0.521 | 259 |
| CEP192 | 18 | 12991361 | 13125051 | 19 | 0.521 | 259 |
| CDK12 | 17 | 37617739 | 37690800 | 18.9 | 0.519 | 259 |
| CD151 | 11 | 832952 | 838835 | 18.9 | -0.52 | 259 |
| GEN1 | 2 | 17935162 | 17966632 | 18.8 | 0.519 | 259 |
| PRC1 | 15 | 91509268 | 91537804 | 18.7 | 0.518 | 259 |
| CASP8AP2 | 6 | 90539619 | 90584155 | 18.6 | 0.516 | 259 |
| TUBGCP4 | 15 | 43663313 | 43698240 | 18.5 | 0.514 | 259 |
| LOC100128191 | 12 | 98906751 | 98910004 | 18.5 | 0.515 | 259 |
| SKP2 | 5 | 36152145 | 36184142 | 18.4 | 0.514 | 259 |
| SCML2 | X | 18257433 | 18372844 | 18.4 | 0.514 | 259 |
| FAM123B | X | 63404997 | 63425624 | 18.4 | 0.513 | 259 |
| TET3 | 2 | 74273450 | 74335302 | 18.3 | 0.512 | 259 |
| PHC3 | 3 | 169805368 | 169899537 | 18.3 | 0.512 | 259 |
| ZNF519 | 18 | 14075989 | 14132489 | 18.3 | 0.512 | 259 |
| PRR11 | 17 | 57232860 | 57284070 | 18.2 | 0.511 | 259 |
| ZBTB33 | X | 119384607 | 119392251 | 18.2 | 0.511 | 259 |
| USP37 | 2 | 219314974 | 219433084 | 18.2 | 0.511 | 259 |
| TTC21B | 2 | 166713986 | 166810348 | 18.2 | 0.511 | 259 |
| TMPO | 12 | 98909351 | 98944157 | 18.2 | 0.511 | 259 |
| NUP153 | 6 | 17615269 | 17706818 | 18.1 | 0.51 | 259 |
| NCAPD2 | 12 | 6603298 | 6641132 | 18.1 | 0.509 | 259 |
| MSI2 | 17 | 55333212 | 55757299 | 18 | 0.509 | 259 |
| HAUS6 | 9 | 19053141 | 19102902 | 18 | 0.509 | 259 |
| KLHL11 | 17 | 40009799 | 40021629 | 17.9 | 0.507 | 259 |
| CENPJ | 13 | 25456412 | 25497085 | 17.9 | 0.507 | 259 |
| DEK | 6 | 18224400 | 18264799 | 17.7 | 0.504 | 259 |
| CIT | 12 | 120123595 | 120315095 | 17.7 | 0.504 | 259 |
| C11ORF82 | 11 | 82612737 | 82669319 | 17.7 | 0.504 | 259 |
| PSMC3IP | 17 | 40724329 | 40729747 | 17.7 | 0.505 | 259 |
| UBXN7 | 3 | 196080369 | 196159345 | 17.6 | 0.503 | 259 |
| RPA1 | 17 | 1733273 | 1802848 | 17.6 | 0.503 | 259 |
| RAD51C | 17 | 56769963 | 56811692 | 17.6 | 0.504 | 259 |
| PLK4 | 4 | 128802016 | 128820377 | 17.6 | 0.503 | 259 |
| DDX46 | 5 | 134094461 | 134166812 | 17.6 | 0.503 | 259 |
| C2ORF44 | 2 | 24252206 | 24270296 | 17.6 | 0.504 | 259 |
| TMEM97 | 17 | 26646121 | 26655711 | 17.5 | 0.501 | 259 |
| LOC730101 | 6 | 52529199 | 52533951 | 17.5 | 0.501 | 259 |
| SLC9A3R2 | 16 | 2076869 | 2089027 | 17.4 | -0.501 | 259 |
| NCAPG2 | 7 | 158424003 | 158497520 | 17.4 | 0.5 | 259 |
| CYB5R1 | 1 | 202931001 | 202936404 | 17.4 | -0.5 | 259 |
| DONSON | 21 | 34947783 | 35288158 | 17.3 | 0.499 | 259 |
| DHX33 | 17 | 5344232 | 5372380 | 17.3 | 0.499 | 259 |
| NOL11 | 17 | 65714061 | 65740266 | 17.3 | 0.499 | 259 |
| ZNF711 | X | 84498997 | 84528368 | 17.2 | 0.497 | 259 |
| PHF6 | X | 133507342 | 133562822 | 17.2 | 0.498 | 259 |
| HELLS | 10 | 96305543 | 96361856 | 17.1 | 0.497 | 259 |
| SUGP2 | 19 | 19101697 | 19144832 | 17.1 | 0.497 | 259 |
| RBM12 | 20 | 34236847 | 34252878 | 17.1 | 0.496 | 259 |
| GSTK1 | 7 | 142960522 | 142966222 | 17 | -0.495 | 259 |
| GMPS | 3 | 155588325 | 155655520 | 17 | 0.496 | 259 |
| PSIP1 | 9 | 15464065 | 15511003 | 16.9 | 0.494 | 259 |
| PDZK1IP1 | 1 | 47649261 | 47655771 | 16.9 | -0.494 | 259 |
| TMEM219 | 16 | 29952206 | 29984373 | 16.8 | -0.492 | 259 |
| VPRBP | 3 | 51433298 | 51534018 | 16.8 | 0.492 | 259 |
| TPX2 | 20 | 30326904 | 30389603 | 16.8 | 0.493 | 259 |
| KANSL3 | 2 | 97258907 | 97304115 | 16.7 | 0.491 | 259 |
| IREB2 | 15 | 78730518 | 78793798 | 16.7 | 0.491 | 259 |
| FAM83D | 20 | 37554955 | 37581703 | 16.7 | 0.492 | 259 |
| TET1 | 10 | 70320117 | 70454239 | 16.7 | 0.49 | 259 |
| ERCC6L2 | 9 | 98637900 | 98801358 | 16.7 | 0.49 | 259 |
| TCF19 | 6 | 31126303 | 31131992 | 16.7 | 0.491 | 259 |
| SIN3A | 15 | 75661720 | 75748124 | 16.7 | 0.491 | 259 |
| MAGEE1 | X | 75648046 | 75651746 | 16.7 | 0.491 | 259 |
| KIF11 | 10 | 94352825 | 94415152 | 16.7 | 0.492 | 259 |
| DIAPH3 | 13 | 60239723 | 60738119 | 16.6 | 0.489 | 259 |
| VASP | 19 | 46010688 | 46030240 | 16.6 | -0.49 | 259 |
| PYGO1 | 15 | 55838221 | 55881050 | 16.6 | 0.489 | 259 |
| LMNB2 | 19 | 2428164 | 2456958 | 16.6 | 0.489 | 259 |
| KPNA2 | 17 | 66031848 | 66042970 | 16.6 | 0.49 | 259 |
| ELAVL1 | 19 | 8023457 | 8070529 | 16.6 | 0.489 | 259 |
| ZNF507 | 19 | 32836514 | 32878573 | 16.5 | 0.488 | 259 |
| SMCHD1 | 18 | 2655886 | 2805015 | 16.5 | 0.489 | 259 |
| KIAA1731 | 11 | 93394816 | 93463522 | 16.5 | 0.488 | 259 |
| CEBPB | 20 | 48807120 | 48809227 | 16.5 | -0.488 | 259 |
| TRA2B | 3 | 185632358 | 185655924 | 16.4 | 0.487 | 259 |
| SERTAD1 | 19 | 40928409 | 40931932 | 16.4 | -0.486 | 259 |
| CRIP2 | 14 | 105939343 | 105946500 | 16.4 | -0.487 | 259 |
| CKAP2L | 2 | 113495444 | 113522254 | 16.4 | 0.487 | 259 |
| CEP97 | 3 | 101443494 | 101486181 | 16.4 | 0.487 | 259 |
| CEP95 | 17 | 62503158 | 62534062 | 16.4 | 0.486 | 259 |
| C17ORF53 | 17 | 42219274 | 42239844 | 16.4 | 0.487 | 259 |
| SMC5 | 9 | 72873878 | 72969789 | 16.3 | 0.486 | 259 |
| MSRB2 | 10 | 23384427 | 23410942 | 16.3 | -0.485 | 259 |
| FKBP2 | 11 | 64008413 | 64011607 | 16.3 | -0.486 | 259 |
| C5ORF25 | 5 | 175665370 | 177099278 | 16.3 | 0.485 | 259 |
| SMC6 | 2 | 17845079 | 17981462 | 16.2 | 0.485 | 259 |
| NDC80 | 18 | 2571510 | 2616634 | 16.2 | 0.484 | 259 |
| HNRNPR | 1 | 23636276 | 23670853 | 16.2 | 0.485 | 259 |
| TYMS | 18 | 657604 | 673499 | 16.2 | 0.484 | 259 |
| VWA1 | 1 | 1370903 | 1378262 | 16.1 | -0.483 | 259 |
| TAF1 | X | 70586114 | 70752224 | 16.1 | 0.483 | 259 |
| BRCA2 | 13 | 32889617 | 32973809 | 29.8 | 0.631 | 259 |
| BRCA1 | 17 | 41196312 | 41322420 | 28.9 | 0.624 | 259 |
| BPTF | 17 | 65821780 | 65980494 | 20.4 | 0.538 | 259 |
| BOK | 2 | 242498146 | 242513553 | 17.1 | -0.497 | 259 |
| BLM | 15 | 91260579 | 91358686 | 29.5 | 0.629 | 259 |
| ATP6V0D1 | 16 | 67471917 | 67515089 | 17 | -0.495 | 259 |
| ATAD5 | 17 | 29159023 | 29222295 | 38.1 | 0.694 | 259 |
| ATAD2B | 2 | 23971534 | 24149936 | 19.3 | 0.524 | 259 |
| ASPM | 1 | 197053257 | 197115824 | 21.2 | 0.547 | 259 |
| ARHGAP11B | 15 | 30916697 | 30977810 | 20.7 | 0.541 | 259 |
| ARHGAP11A | 15 | 32907345 | 32931868 | 20.3 | 0.537 | 259 |
| ANKRD40 | 17 | 48770551 | 48785270 | 17.9 | 0.507 | 259 |
| ANAPC1 | 2 | 87407211 | 112641741 | 20.2 | 0.536 | 259 |
| AGTRAP | 1 | 11796142 | 11810828 | 17.5 | -0.502 | 259 |
| LUSC: Lung squamous cell carcinoma, *: Chromosome, a: chromosome starting site of the gene, b: chromosome ending site of the gene. | | | | | | |

| Table S8. The correlation between BRIP1 and other genes of OV from the TCGA database | | | | | | |
| --- | --- | --- | --- | --- | --- | --- |
| Genes | Chr^*^ | Start^a^ | End^b^ | -Log10 (p) | Correlation | Samples |
| TTK | 6 | 80714322 | 80752244 | 48.4 | 0.561 | 574 |
| CASC5 | 15 | 40886447 | 40954881 | 47.6 | 0.557 | 574 |
| CDC6 | 17 | 38444146 | 38459413 | 46.6 | 0.552 | 574 |
| KPNA2 | 17 | 66031848 | 66042970 | 45.5 | 0.546 | 574 |
| MCM10 | 10 | 13203554 | 13253104 | 41.6 | 0.525 | 574 |
| NCAPH | 2 | 97001484 | 97041274 | 40.9 | 0.521 | 574 |
| SKA2 | 17 | 57187308 | 57232800 | 40.7 | 0.52 | 574 |
| KIF18A | 11 | 28042163 | 28129746 | 39.9 | 0.516 | 574 |
| NDC80 | 18 | 2571510 | 2616634 | 39.3 | 0.512 | 574 |
| DNA2 | 10 | 70173821 | 70231879 | 39.3 | 0.512 | 574 |
| UBE2T | 1 | 202300785 | 202311094 | 37.5 | 0.502 | 574 |
| EXO1 | 1 | 242011493 | 242053241 | 37.4 | 0.501 | 574 |
| DEPDC1 | 1 | 68939835 | 68962799 | 36.8 | 0.498 | 574 |
| BUB1 | 2 | 111395409 | 111435684 | 36.7 | 0.497 | 574 |
| MED13 | 17 | 60019966 | 60142643 | 36.1 | 0.493 | 574 |
| TYMS | 18 | 657604 | 673499 | 35.9 | 0.492 | 574 |
| DLGAP5 | 14 | 55614834 | 55658396 | 35.6 | 0.49 | 574 |
| KIF23 | 15 | 69706627 | 69740764 | 34.2 | 0.481 | 574 |
| CCNA2 | 4 | 122737599 | 122745088 | 34.1 | 0.48 | 574 |
| KIF2C | 1 | 45205490 | 45233438 | 34 | 0.48 | 574 |
| CHEK1 | 11 | 125495031 | 125546150 | 34 | 0.48 | 574 |
| SKA1 | 18 | 47901392 | 47920538 | 33.8 | 0.479 | 574 |
| CENPA | 2 | 27008882 | 27023934 | 33.7 | 0.478 | 574 |
| CDC25C | 5 | 137620959 | 137674044 | 33.6 | 0.478 | 574 |
| NEIL3 | 4 | 178230991 | 178284092 | 33.4 | 0.476 | 574 |
| CENPE | 4 | 104026963 | 104119566 | 33.4 | 0.476 | 574 |
| CDCA5 | 11 | 64844927 | 64851615 | 33.3 | 0.476 | 574 |
| WDHD1 | 14 | 55405656 | 55493819 | 33.2 | 0.475 | 574 |
| MELK | 9 | 36572905 | 36677679 | 33.2 | 0.475 | 574 |
| KIAA0101 | 15 | 64657211 | 64673702 | 33.1 | 0.474 | 574 |
| TK1 | 17 | 76170160 | 76183285 | 32.9 | 0.473 | 574 |
| ZWINT | 10 | 58117199 | 58121034 | 32.8 | 0.473 | 574 |
| CCNB2 | 15 | 59397284 | 59417244 | 32.8 | 0.472 | 574 |
| RAD54L | 1 | 46713367 | 46744145 | 32.7 | 0.472 | 574 |
| FANCI | 15 | 89787194 | 89860362 | 32.5 | 0.471 | 574 |
| KIF14 | 1 | 200520625 | 200589862 | 32.2 | 0.468 | 574 |
| NUF2 | 1 | 163291723 | 163325553 | 32 | 0.467 | 574 |
| FBXO5 | 6 | 153291658 | 153304740 | 32 | 0.467 | 574 |
| TPX2 | 20 | 30326904 | 30389603 | 31.8 | 0.466 | 574 |
| PPM1D | 17 | 58677544 | 58743640 | 31.8 | 0.466 | 574 |
| RACGAP1 | 12 | 50382945 | 50419307 | 31.7 | 0.465 | 574 |
| HELLS | 10 | 96305543 | 96361856 | 31.7 | 0.465 | 574 |
| ERCC6L | X | 71424507 | 71458858 | 31.6 | 0.464 | 574 |
| DONSON | 21 | 34947783 | 35288158 | 31.5 | 0.464 | 574 |
| KIFC1 | 6 | 33359313 | 33377699 | 31.3 | 0.462 | 574 |
| ECT2 | 3 | 172468532 | 172539263 | 31.3 | 0.462 | 574 |
| TOP2A | 17 | 38544773 | 38574202 | 31.1 | 0.461 | 574 |
| BUB1B | 15 | 40453210 | 40513337 | 31.1 | 0.461 | 574 |
| KIF11 | 10 | 94352825 | 94415152 | 30.7 | 0.458 | 574 |
| CENPI | X | 100353178 | 100417978 | 30.7 | 0.458 | 574 |
| MMS22L | 6 | 97590037 | 97731061 | 30.5 | 0.457 | 574 |
| E2F8 | 11 | 19245610 | 19263167 | 30.5 | 0.457 | 574 |
| KIF4B | 5 | 154393260 | 154397685 | 30.3 | 0.455 | 574 |
| ORC1 | 1 | 52838501 | 52870143 | 30 | 0.454 | 574 |
| TMPO | 12 | 98909351 | 98944157 | 29.6 | 0.45 | 574 |
| CDC45 | 22 | 19467414 | 19508135 | 29.6 | 0.45 | 574 |
| SPAG5 | 17 | 26904583 | 26941211 | 29.4 | 0.449 | 574 |
| RRM2 | 2 | 10262695 | 10271546 | 29.4 | 0.449 | 574 |
| DEPDC1B | 5 | 59892739 | 59995993 | 29.4 | 0.449 | 574 |
| OIP5 | 15 | 41601466 | 41624819 | 29.3 | 0.448 | 574 |
| TIMELESS | 12 | 56810157 | 56843200 | 29.2 | 0.448 | 574 |
| PLK4 | 4 | 128802016 | 128820377 | 28.9 | 0.445 | 574 |
| C15ORF42 | 15 | 90118818 | 90171253 | 28.9 | 0.445 | 574 |
| MND1 | 4 | 154265801 | 154336247 | 28.8 | 0.444 | 574 |
| CKAP2L | 2 | 113495444 | 113522254 | 28.6 | 0.443 | 574 |
| CDC7 | 1 | 91966404 | 91991321 | 28.4 | 0.442 | 574 |
| GINS2 | 16 | 85711280 | 85722588 | 28.3 | 0.441 | 574 |
| KIF4A | X | 69509879 | 69640774 | 28 | 0.439 | 574 |
| SPC25 | 2 | 169727401 | 169746944 | 27.9 | 0.438 | 574 |
| NUSAP1 | 15 | 41624892 | 41673248 | 27.9 | 0.438 | 574 |
| RBL1 | 20 | 35626178 | 35724410 | 27.7 | 0.436 | 574 |
| KNTC1 | 12 | 123011809 | 123110947 | 27.7 | 0.437 | 574 |
| KIF15 | 3 | 44803209 | 44894748 | 27.7 | 0.436 | 574 |
| FAM54A | 6 | 136552168 | 136571449 | 27.7 | 0.437 | 574 |
| WDR76 | 15 | 44119112 | 44160617 | 27.7 | 0.437 | 574 |
| GMNN | 6 | 24775159 | 24786325 | 27.6 | 0.436 | 574 |
| FEN1 | 11 | 61560109 | 61564714 | 27.6 | 0.436 | 574 |
| CDCA8 | 1 | 38158159 | 38175391 | 27.2 | 0.433 | 574 |
| MCM3 | 6 | 52128812 | 52149582 | 27.1 | 0.432 | 574 |
| TADA2A | 17 | 35766977 | 35837226 | 27.1 | 0.432 | 574 |
| GPSM2 | 1 | 109419603 | 109476957 | 26.7 | 0.429 | 574 |
| STIL | 1 | 47715811 | 47779819 | 26.5 | 0.428 | 574 |
| NEK2 | 1 | 211831599 | 211848972 | 26.5 | 0.428 | 574 |
| POLG | 15 | 89859536 | 89878026 | 26.1 | 0.424 | 574 |
| MCM4 | 8 | 48872763 | 48890719 | 26.1 | 0.425 | 574 |
| E2F1 | 20 | 32263292 | 32274210 | 26 | 0.423 | 574 |
| CENPM | 22 | 42334741 | 42343148 | 25.4 | 0.419 | 574 |
| INTS2 | 17 | 59942728 | 60005377 | 25.3 | 0.418 | 574 |
| DTL | 1 | 212208919 | 212278187 | 25.3 | 0.418 | 574 |
| CENPW | 6 | 126661253 | 126669754 | 25.3 | 0.419 | 574 |
| CENPO | 2 | 25016175 | 25045245 | 25.3 | 0.418 | 574 |
| POLQ | 3 | 121150273 | 121264853 | 24.9 | 0.415 | 574 |
| CCNB1 | 5 | 68462837 | 68474070 | 24.9 | 0.415 | 574 |
| PTTG1 | 5 | 159848865 | 159855746 | 24.8 | 0.414 | 574 |
| PTTG3P | 8 | 67679632 | 67680240 | 24.6 | 0.413 | 574 |
| RAD51 | 15 | 40987327 | 41024356 | 24.5 | 0.412 | 574 |
| MCM6 | 2 | 136597196 | 136634011 | 24.5 | 0.412 | 574 |
| CCNE2 | 8 | 95892453 | 95907482 | 24.5 | 0.412 | 574 |
| PTTG2 | 4 | 37962056 | 37962631 | 24.4 | 0.411 | 574 |
| SMC4 | 3 | 160117430 | 160152741 | 24.2 | 0.409 | 574 |
| C11ORF82 | 11 | 82612737 | 82669319 | 24.1 | 0.409 | 574 |
| RAD51C | 17 | 56769963 | 56811692 | 24 | 0.408 | 574 |
| C1ORF112 | 1 | 169631245 | 169822229 | 24 | 0.408 | 574 |
| PRIM2 | 6 | 57182422 | 57513376 | 23.8 | 0.406 | 574 |
| UBE2C | 20 | 44441255 | 44445596 | 23.8 | 0.406 | 574 |
| GINS1 | 20 | 25388323 | 25429191 | 23.7 | 0.406 | 574 |
| MCM2 | 3 | 127317253 | 127341278 | 23.7 | 0.405 | 574 |
| GSG2 | 17 | 3627197 | 3629992 | 23.7 | 0.405 | 574 |
| PCNA | 20 | 5095599 | 5107268 | 23.5 | 0.404 | 574 |
| CENPF | 1 | 214776532 | 214837914 | 23.5 | 0.404 | 574 |
| TCF19 | 6 | 31126303 | 31131992 | 23.4 | 0.403 | 574 |
| CIT | 12 | 120123595 | 120315095 | 23.3 | 0.402 | 574 |
| CEP55 | 10 | 95256369 | 95288849 | 23.2 | 0.402 | 574 |
| C5ORF34 | 5 | 43486803 | 43515273 | 23.2 | 0.402 | 574 |
| SUMO2 | 17 | 73163825 | 73179098 | 23.1 | 0.401 | 574 |
| STMN1 | 1 | 26210677 | 26233368 | 23 | 0.399 | 574 |
| RFC4 | 3 | 186507682 | 186524484 | 23 | 0.4 | 574 |
| PRC1 | 15 | 91509268 | 91537804 | 22.9 | 0.398 | 574 |
| E2F7 | 12 | 77415026 | 77459360 | 22.7 | 0.397 | 574 |
| C17ORF53 | 17 | 42219274 | 42239844 | 22.4 | 0.395 | 574 |
| PBK | 8 | 27667138 | 27695572 | 22.4 | 0.394 | 574 |
| CDK1 | 10 | 62538089 | 62554610 | 22.4 | 0.394 | 574 |
| SMG8 | 17 | 57287371 | 57292611 | 22.3 | 0.394 | 574 |
| HMGB2 | 4 | 174252527 | 174255595 | 22.3 | 0.393 | 574 |
| KIF20A | 5 | 137514417 | 137523404 | 22.2 | 0.393 | 574 |
| CDCA7 | 2 | 174219561 | 174233718 | 22.1 | 0.392 | 574 |
| UHRF1 | 19 | 4909510 | 4962165 | 22 | 0.391 | 574 |
| RMI2 | 16 | 11343506 | 11445617 | 22 | 0.391 | 574 |
| DCAF7 | 17 | 61627822 | 61671642 | 22 | 0.391 | 574 |
| PARPBP | 12 | 102513956 | 102591298 | 21.9 | 0.391 | 574 |
| TUBA1B | 12 | 49521565 | 49525304 | 21.8 | 0.389 | 574 |
| ESCO2 | 8 | 27632058 | 27670141 | 21.7 | 0.388 | 574 |
| DTX4 | 11 | 58938903 | 58976060 | 21.7 | -0.389 | 574 |
| POLA2 | 11 | 65029432 | 65065088 | 21.6 | 0.388 | 574 |
| CLSPN | 1 | 36197713 | 36235551 | 21.6 | 0.388 | 574 |
| PRPSAP1 | 17 | 74306868 | 74350279 | 21.3 | 0.385 | 574 |
| MYBL2 | 20 | 42295709 | 42345122 | 21.2 | 0.384 | 574 |
| CENPL | 1 | 173768688 | 173793777 | 21.2 | 0.384 | 574 |
| CDC20 | 1 | 43824626 | 43828873 | 21.1 | 0.384 | 574 |
| PRIM1 | 12 | 57125364 | 57146146 | 21.1 | 0.384 | 574 |
| SMC2 | 9 | 106856541 | 106903700 | 21 | 0.382 | 574 |
| METTL2B | 7 | 128116783 | 128142978 | 21 | 0.382 | 574 |
| TUBD1 | 17 | 57936841 | 57970306 | 20.9 | 0.382 | 574 |
| CLU | 8 | 27454434 | 27472328 | 20.8 | -0.381 | 574 |
| SUPT4H1 | 17 | 56422539 | 56429563 | 20.7 | 0.38 | 574 |
| MASTL | 10 | 27443753 | 27475848 | 20.7 | 0.38 | 574 |
| H2AFX | 11 | 118964585 | 118966177 | 20.7 | 0.38 | 574 |
| CENPN | 16 | 81040103 | 81066709 | 20.5 | 0.378 | 574 |
| LIN54 | 4 | 83845756 | 83934040 | 20.4 | 0.377 | 574 |
| TTF2 | 1 | 117602949 | 117645491 | 20.4 | 0.377 | 574 |
| C16ORF59 | 16 | 2510115 | 2514964 | 20.2 | 0.375 | 574 |
| SRSF12 | 6 | 89805678 | 89827800 | 20.1 | 0.374 | 574 |
| HAUS8 | 19 | 17160571 | 17186343 | 20.1 | 0.375 | 574 |
| SENP5 | 3 | 196594727 | 196661584 | 19.9 | 0.372 | 574 |
| CHRNA5 | 15 | 78857862 | 78887611 | 19.9 | 0.373 | 574 |
| KLHL11 | 17 | 40009799 | 40021629 | 19.7 | 0.37 | 574 |
| TPT1 | 13 | 45911304 | 45915318 | 19.7 | -0.37 | 574 |
| FAM83D | 20 | 37554955 | 37581703 | 19.5 | 0.369 | 574 |
| CEP76 | 18 | 12661955 | 12702703 | 19.5 | 0.369 | 574 |
| RTKN2 | 10 | 63942794 | 64028466 | 19.3 | 0.367 | 574 |
| MAD2L1 | 4 | 120980579 | 120988013 | 19.1 | 0.365 | 574 |
| DGKE | 17 | 54911460 | 54946036 | 19.1 | 0.365 | 574 |
| FAM64A | 17 | 6347735 | 6354385 | 19 | 0.364 | 574 |
| MSH6 | 2 | 48010221 | 48034092 | 18.9 | 0.363 | 574 |
| MRPL27 | 17 | 48445228 | 48450562 | 18.9 | 0.363 | 574 |
| CNTLN | 9 | 17135038 | 17503917 | 18.9 | 0.363 | 574 |
| WDR5 | 9 | 137001210 | 137025094 | 18.9 | 0.363 | 574 |
| SUV39H2 | 10 | 14920782 | 14946304 | 18.9 | 0.364 | 574 |
| GAS2L3 | 12 | 100967489 | 101018685 | 18.8 | 0.362 | 574 |
| CDC27 | 17 | 45195311 | 45266665 | 18.8 | 0.362 | 574 |
| LOC401127 | 4 | 39481875 | 39483523 | 18.8 | 0.363 | 574 |
| SKA3 | 13 | 21727734 | 21750741 | 18.7 | 0.361 | 574 |
| SCAI | 9 | 127704888 | 127905838 | 18.7 | 0.361 | 574 |
| ILF2 | 1 | 153634514 | 153643479 | 18.7 | 0.361 | 574 |
| TRIM37 | 17 | 57060000 | 57184266 | 18.6 | 0.36 | 574 |
| BLM | 15 | 91260579 | 91358686 | 28.3 | 0.441 | 574 |
| BIRC5 | 17 | 76210277 | 76221716 | 31.2 | 0.462 | 574 |
| AURKB | 17 | 8108049 | 8113883 | 22.3 | 0.394 | 574 |
| AURKA | 20 | 54944445 | 54967351 | 24.2 | 0.41 | 574 |
| ATAD5 | 17 | 29159023 | 29222295 | 42.6 | 0.531 | 574 |
| ATAD2 | 8 | 124332091 | 124428590 | 25 | 0.416 | 574 |
| ASPM | 1 | 197053257 | 197115824 | 33.6 | 0.477 | 574 |
| ARHGAP11A | 15 | 32907345 | 32931868 | 33.6 | 0.478 | 574 |
| ANLN | 7 | 36429432 | 36493400 | 23.4 | 0.403 | 574 |
| ANKRD40 | 17 | 48770551 | 48785270 | 26.3 | 0.426 | 574 |
| ANKRD32 | 5 | 93954391 | 94031573 | 21.1 | 0.383 | 574 |
| ALS2CL | 3 | 46710485 | 46735194 | 21.1 | -0.383 | 574 |
| OV: Ovarian serous cystadenocarcinoma, *: Chromosome, a: chromosome starting site of the gene, b: chromosome ending site of the gene. | | | | | | |

| Table S9. The correlation between BRIP1 and other genes of THCA from the TCGA database | | | | | | |
| --- | --- | --- | --- | --- | --- | --- |
| Genes | Chr^*^ | Start^a^ | End^b^ | -Log10 (p) | Correlation | Samples |
| CLSPN | 1 | 36185819 | 36235568 | 158.9 | 0.88 | 482 |
| MCM4 | 8 | 48872745 | 48890720 | 126.1 | 0.84 | 482 |
| DTL | 1 | 212208919 | 212280742 | 122.1 | 0.83 | 482 |
| MCM10 | 10 | 13203554 | 13253104 | 114.4 | 0.81 | 482 |
| FANCI | 15 | 89787180 | 89860492 | 114.1 | 0.81 | 482 |
| HELLS | 10 | 96305547 | 96373662 | 113.9 | 0.81 | 482 |
| WDHD1 | 14 | 55405668 | 55493823 | 111.9 | 0.81 | 482 |
| WDR76 | 15 | 44119161 | 44160617 | 106.7 | 0.8 | 482 |
| UHRF1 | 19 | 4903092 | 4962165 | 100.2 | 0.78 | 482 |
| FANCD2 | 3 | 10068098 | 10143614 | 100 | 0.78 | 482 |
| RAD51AP1 | 12 | 4647950 | 4669214 | 99.9 | 0.78 | 482 |
| XRCC2 | 7 | 152341864 | 152373250 | 96.9 | 0.77 | 482 |
| FAM111B | 11 | 58874658 | 58894883 | 96.7 | 0.77 | 482 |
| CCNE2 | 8 | 95891998 | 95908906 | 95.5 | 0.77 | 482 |
| TCF19 | 6 | 31126319 | 31134936 | 94.7 | 0.77 | 482 |
| CDC6 | 17 | 38443885 | 38459171 | 94.3 | 0.77 | 482 |
| ORC1 | 1 | 52838501 | 52870143 | 92.4 | 0.76 | 482 |
| MCM6 | 2 | 136597196 | 136633996 | 92 | 0.76 | 482 |
| KNTC1 | 12 | 123011793 | 123110943 | 89.6 | 0.76 | 482 |
| E2F7 | 12 | 77415027 | 77459360 | 88.3 | 0.75 | 482 |
| CENPK | 5 | 64813593 | 64858998 | 87.2 | 0.75 | 482 |
| GINS1 | 20 | 25388363 | 25433264 | 86.6 | 0.75 | 482 |
| STIL | 1 | 47715811 | 47779819 | 81.8 | 0.73 | 482 |
| EXO1 | 1 | 242011269 | 242058450 | 79.3 | 0.73 | 482 |
| ZWILCH | 15 | 66797297 | 66842115 | 77.9 | 0.72 | 482 |
| GINS4 | 8 | 41386725 | 41402565 | 77.3 | 0.72 | 482 |
| ZNF367 | 9 | 99148223 | 99180611 | 75.3 | 0.71 | 482 |
| NCAPG2 | 7 | 158424003 | 158497520 | 75.3 | 0.71 | 482 |
| TYMS | 18 | 657604 | 673578 | 74.2 | 0.71 | 482 |
| MMS22L | 6 | 97590037 | 97731061 | 74.2 | 0.71 | 482 |
| MELK | 9 | 36572859 | 36677678 | 73.4 | 0.71 | 482 |
| TIMELESS | 12 | 56810903 | 56843187 | 68.5 | 0.69 | 482 |
| PLK4 | 4 | 128802016 | 128820350 | 68 | 0.69 | 482 |
| ESCO2 | 8 | 27629466 | 27670157 | 67.6 | 0.69 | 482 |
| LMNB1 | 5 | 126112315 | 126172712 | 67.5 | 0.69 | 482 |
| POLQ | 3 | 121150278 | 121264853 | 66.6 | 0.68 | 482 |
| CDC45 | 22 | 19466982 | 19508135 | 64.4 | 0.67 | 482 |
| TOPBP1 | 3 | 133317019 | 133380737 | 63.2 | 0.67 | 482 |
| BUB1 | 2 | 111395275 | 111435691 | 63.1 | 0.67 | 482 |
| CDK2 | 12 | 56360553 | 56366568 | 63 | 0.67 | 482 |
| DNMT1 | 19 | 10244021 | 10341962 | 62.7 | 0.67 | 482 |
| RAD51 | 15 | 40986972 | 41024354 | 62 | 0.66 | 482 |
| PRC1 | 15 | 91509270 | 91538859 | 60.5 | 0.66 | 482 |
| TOP2A | 17 | 38544768 | 38574202 | 60.3 | 0.66 | 482 |
| CASC5 | 15 | 40886218 | 40956540 | 59.9 | 0.66 | 482 |
| KIF11 | 10 | 94353043 | 94415150 | 58.2 | 0.65 | 482 |
| C11orf82 | 11 | 82611017 | 82669319 | 57.4 | 0.65 | 482 |
| NUSAP1 | 15 | 41624892 | 41673248 | 57.3 | 0.65 | 482 |
| FANCA | 16 | 89803957 | 89883065 | 57 | 0.64 | 482 |
| ERCC6L | X | 71424510 | 71458897 | 57 | 0.64 | 482 |
| BRCA2 | 13 | 32889611 | 32973805 | 121.2 | 0.83 | 482 |
| ATAD5 | 17 | 29158988 | 29222887 | 71.8 | 0.7 | 482 |
| ATAD2 | 8 | 124332090 | 124428590 | 79.5 | 0.73 | 482 |
| ASF1B | 19 | 14230321 | 14247768 | 65.9 | 0.68 | 482 |
| THCA: Thyroid carcinoma, *: Chromosome, a: chromosome starting site of the gene, b: chromosome ending site of the gene. | | | | | | |

| Table S10. The correlation between BRIP1 and other genes of UCEC from the TCGA database | | | | | | |
| --- | --- | --- | --- | --- | --- | --- |
| Genes | Chr^*^ | Start^a^ | End^b^ | -Log10 (p) | Correlation | Samples |
| TTK | 6 | 80714322 | 80752244 | 48.4 | 0.561 | 574 |
| CASC5 | 15 | 40886447 | 40954881 | 47.6 | 0.557 | 574 |
| CDC6 | 17 | 38444146 | 38459413 | 46.6 | 0.552 | 574 |
| KPNA2 | 17 | 66031848 | 66042970 | 45.5 | 0.546 | 574 |
| MCM10 | 10 | 13203554 | 13253104 | 41.6 | 0.525 | 574 |
| NCAPH | 2 | 97001484 | 97041274 | 40.9 | 0.521 | 574 |
| SKA2 | 17 | 57187308 | 57232800 | 40.7 | 0.52 | 574 |
| KIF18A | 11 | 28042163 | 28129746 | 39.9 | 0.516 | 574 |
| NDC80 | 18 | 2571510 | 2616634 | 39.3 | 0.512 | 574 |
| DNA2 | 10 | 70173821 | 70231879 | 39.3 | 0.512 | 574 |
| UBE2T | 1 | 202300785 | 202311094 | 37.5 | 0.502 | 574 |
| EXO1 | 1 | 242011493 | 242053241 | 37.4 | 0.501 | 574 |
| DEPDC1 | 1 | 68939835 | 68962799 | 36.8 | 0.498 | 574 |
| BUB1 | 2 | 111395409 | 111435684 | 36.7 | 0.497 | 574 |
| MED13 | 17 | 60019966 | 60142643 | 36.1 | 0.493 | 574 |
| TYMS | 18 | 657604 | 673499 | 35.9 | 0.492 | 574 |
| DLGAP5 | 14 | 55614834 | 55658396 | 35.6 | 0.49 | 574 |
| KIF23 | 15 | 69706627 | 69740764 | 34.2 | 0.481 | 574 |
| CCNA2 | 4 | 122737599 | 122745088 | 34.1 | 0.48 | 574 |
| KIF2C | 1 | 45205490 | 45233438 | 34 | 0.48 | 574 |
| CHEK1 | 11 | 125495031 | 125546150 | 34 | 0.48 | 574 |
| SKA1 | 18 | 47901392 | 47920538 | 33.8 | 0.479 | 574 |
| CENPA | 2 | 27008882 | 27023934 | 33.7 | 0.478 | 574 |
| CDC25C | 5 | 137620959 | 137674044 | 33.6 | 0.478 | 574 |
| NEIL3 | 4 | 178230991 | 178284092 | 33.4 | 0.476 | 574 |
| CENPE | 4 | 104026963 | 104119566 | 33.4 | 0.476 | 574 |
| CDCA5 | 11 | 64844927 | 64851615 | 33.3 | 0.476 | 574 |
| WDHD1 | 14 | 55405656 | 55493819 | 33.2 | 0.475 | 574 |
| MELK | 9 | 36572905 | 36677679 | 33.2 | 0.475 | 574 |
| KIAA0101 | 15 | 64657211 | 64673702 | 33.1 | 0.474 | 574 |
| TK1 | 17 | 76170160 | 76183285 | 32.9 | 0.473 | 574 |
| ZWINT | 10 | 58117199 | 58121034 | 32.8 | 0.473 | 574 |
| CCNB2 | 15 | 59397284 | 59417244 | 32.8 | 0.472 | 574 |
| RAD54L | 1 | 46713367 | 46744145 | 32.7 | 0.472 | 574 |
| FANCI | 15 | 89787194 | 89860362 | 32.5 | 0.471 | 574 |
| KIF14 | 1 | 200520625 | 200589862 | 32.2 | 0.468 | 574 |
| NUF2 | 1 | 163291723 | 163325553 | 32 | 0.467 | 574 |
| FBXO5 | 6 | 153291658 | 153304740 | 32 | 0.467 | 574 |
| TPX2 | 20 | 30326904 | 30389603 | 31.8 | 0.466 | 574 |
| PPM1D | 17 | 58677544 | 58743640 | 31.8 | 0.466 | 574 |
| RACGAP1 | 12 | 50382945 | 50419307 | 31.7 | 0.465 | 574 |
| HELLS | 10 | 96305543 | 96361856 | 31.7 | 0.465 | 574 |
| ERCC6L | X | 71424507 | 71458858 | 31.6 | 0.464 | 574 |
| DONSON | 21 | 34947783 | 35288158 | 31.5 | 0.464 | 574 |
| KIFC1 | 6 | 33359313 | 33377699 | 31.3 | 0.462 | 574 |
| ECT2 | 3 | 172468532 | 172539263 | 31.3 | 0.462 | 574 |
| TOP2A | 17 | 38544773 | 38574202 | 31.1 | 0.461 | 574 |
| BUB1B | 15 | 40453210 | 40513337 | 31.1 | 0.461 | 574 |
| KIF11 | 10 | 94352825 | 94415152 | 30.7 | 0.458 | 574 |
| CENPI | X | 100353178 | 100417978 | 30.7 | 0.458 | 574 |
| MMS22L | 6 | 97590037 | 97731061 | 30.5 | 0.457 | 574 |
| E2F8 | 11 | 19245610 | 19263167 | 30.5 | 0.457 | 574 |
| KIF4B | 5 | 154393260 | 154397685 | 30.3 | 0.455 | 574 |
| ORC1 | 1 | 52838501 | 52870143 | 30 | 0.454 | 574 |
| TMPO | 12 | 98909351 | 98944157 | 29.6 | 0.45 | 574 |
| CDC45 | 22 | 19467414 | 19508135 | 29.6 | 0.45 | 574 |
| SPAG5 | 17 | 26904583 | 26941211 | 29.4 | 0.449 | 574 |
| RRM2 | 2 | 10262695 | 10271546 | 29.4 | 0.449 | 574 |
| DEPDC1B | 5 | 59892739 | 59995993 | 29.4 | 0.449 | 574 |
| OIP5 | 15 | 41601466 | 41624819 | 29.3 | 0.448 | 574 |
| TIMELESS | 12 | 56810157 | 56843200 | 29.2 | 0.448 | 574 |
| PLK4 | 4 | 128802016 | 128820377 | 28.9 | 0.445 | 574 |
| C15ORF42 | 15 | 90118818 | 90171253 | 28.9 | 0.445 | 574 |
| MND1 | 4 | 154265801 | 154336247 | 28.8 | 0.444 | 574 |
| CKAP2L | 2 | 113495444 | 113522254 | 28.6 | 0.443 | 574 |
| CDC7 | 1 | 91966404 | 91991321 | 28.4 | 0.442 | 574 |
| GINS2 | 16 | 85711280 | 85722588 | 28.3 | 0.441 | 574 |
| KIF4A | X | 69509879 | 69640774 | 28 | 0.439 | 574 |
| SPC25 | 2 | 169727401 | 169746944 | 27.9 | 0.438 | 574 |
| NUSAP1 | 15 | 41624892 | 41673248 | 27.9 | 0.438 | 574 |
| RBL1 | 20 | 35626178 | 35724410 | 27.7 | 0.436 | 574 |
| KNTC1 | 12 | 123011809 | 123110947 | 27.7 | 0.437 | 574 |
| KIF15 | 3 | 44803209 | 44894748 | 27.7 | 0.436 | 574 |
| FAM54A | 6 | 136552168 | 136571449 | 27.7 | 0.437 | 574 |
| WDR76 | 15 | 44119112 | 44160617 | 27.7 | 0.437 | 574 |
| GMNN | 6 | 24775159 | 24786325 | 27.6 | 0.436 | 574 |
| FEN1 | 11 | 61560109 | 61564714 | 27.6 | 0.436 | 574 |
| CDCA8 | 1 | 38158159 | 38175391 | 27.2 | 0.433 | 574 |
| MCM3 | 6 | 52128812 | 52149582 | 27.1 | 0.432 | 574 |
| TADA2A | 17 | 35766977 | 35837226 | 27.1 | 0.432 | 574 |
| GPSM2 | 1 | 109419603 | 109476957 | 26.7 | 0.429 | 574 |
| STIL | 1 | 47715811 | 47779819 | 26.5 | 0.428 | 574 |
| NEK2 | 1 | 211831599 | 211848972 | 26.5 | 0.428 | 574 |
| POLG | 15 | 89859536 | 89878026 | 26.1 | 0.424 | 574 |
| MCM4 | 8 | 48872763 | 48890719 | 26.1 | 0.425 | 574 |
| E2F1 | 20 | 32263292 | 32274210 | 26 | 0.423 | 574 |
| CENPM | 22 | 42334741 | 42343148 | 25.4 | 0.419 | 574 |
| INTS2 | 17 | 59942728 | 60005377 | 25.3 | 0.418 | 574 |
| DTL | 1 | 212208919 | 212278187 | 25.3 | 0.418 | 574 |
| CENPW | 6 | 126661253 | 126669754 | 25.3 | 0.419 | 574 |
| CENPO | 2 | 25016175 | 25045245 | 25.3 | 0.418 | 574 |
| POLQ | 3 | 121150273 | 121264853 | 24.9 | 0.415 | 574 |
| CCNB1 | 5 | 68462837 | 68474070 | 24.9 | 0.415 | 574 |
| PTTG1 | 5 | 159848865 | 159855746 | 24.8 | 0.414 | 574 |
| PTTG3P | 8 | 67679632 | 67680240 | 24.6 | 0.413 | 574 |
| RAD51 | 15 | 40987327 | 41024356 | 24.5 | 0.412 | 574 |
| MCM6 | 2 | 136597196 | 136634011 | 24.5 | 0.412 | 574 |
| CCNE2 | 8 | 95892453 | 95907482 | 24.5 | 0.412 | 574 |
| PTTG2 | 4 | 37962056 | 37962631 | 24.4 | 0.411 | 574 |
| SMC4 | 3 | 160117430 | 160152741 | 24.2 | 0.409 | 574 |
| C11ORF82 | 11 | 82612737 | 82669319 | 24.1 | 0.409 | 574 |
| RAD51C | 17 | 56769963 | 56811692 | 24 | 0.408 | 574 |
| C1ORF112 | 1 | 169631245 | 169822229 | 24 | 0.408 | 574 |
| PRIM2 | 6 | 57182422 | 57513376 | 23.8 | 0.406 | 574 |
| UBE2C | 20 | 44441255 | 44445596 | 23.8 | 0.406 | 574 |
| GINS1 | 20 | 25388323 | 25429191 | 23.7 | 0.406 | 574 |
| MCM2 | 3 | 127317253 | 127341278 | 23.7 | 0.405 | 574 |
| GSG2 | 17 | 3627197 | 3629992 | 23.7 | 0.405 | 574 |
| PCNA | 20 | 5095599 | 5107268 | 23.5 | 0.404 | 574 |
| CENPF | 1 | 214776532 | 214837914 | 23.5 | 0.404 | 574 |
| TCF19 | 6 | 31126303 | 31131992 | 23.4 | 0.403 | 574 |
| CIT | 12 | 120123595 | 120315095 | 23.3 | 0.402 | 574 |
| CEP55 | 10 | 95256369 | 95288849 | 23.2 | 0.402 | 574 |
| C5ORF34 | 5 | 43486803 | 43515273 | 23.2 | 0.402 | 574 |
| SUMO2 | 17 | 73163825 | 73179098 | 23.1 | 0.401 | 574 |
| STMN1 | 1 | 26210677 | 26233368 | 23 | 0.399 | 574 |
| RFC4 | 3 | 186507682 | 186524484 | 23 | 0.4 | 574 |
| PRC1 | 15 | 91509268 | 91537804 | 22.9 | 0.398 | 574 |
| E2F7 | 12 | 77415026 | 77459360 | 22.7 | 0.397 | 574 |
| C17ORF53 | 17 | 42219274 | 42239844 | 22.4 | 0.395 | 574 |
| PBK | 8 | 27667138 | 27695572 | 22.4 | 0.394 | 574 |
| CDK1 | 10 | 62538089 | 62554610 | 22.4 | 0.394 | 574 |
| SMG8 | 17 | 57287371 | 57292611 | 22.3 | 0.394 | 574 |
| HMGB2 | 4 | 174252527 | 174255595 | 22.3 | 0.393 | 574 |
| KIF20A | 5 | 137514417 | 137523404 | 22.2 | 0.393 | 574 |
| CDCA7 | 2 | 174219561 | 174233718 | 22.1 | 0.392 | 574 |
| UHRF1 | 19 | 4909510 | 4962165 | 22 | 0.391 | 574 |
| RMI2 | 16 | 11343506 | 11445617 | 22 | 0.391 | 574 |
| DCAF7 | 17 | 61627822 | 61671642 | 22 | 0.391 | 574 |
| PARPBP | 12 | 102513956 | 102591298 | 21.9 | 0.391 | 574 |
| TUBA1B | 12 | 49521565 | 49525304 | 21.8 | 0.389 | 574 |
| ESCO2 | 8 | 27632058 | 27670141 | 21.7 | 0.388 | 574 |
| DTX4 | 11 | 58938903 | 58976060 | 21.7 | -0.389 | 574 |
| POLA2 | 11 | 65029432 | 65065088 | 21.6 | 0.388 | 574 |
| CLSPN | 1 | 36197713 | 36235551 | 21.6 | 0.388 | 574 |
| PRPSAP1 | 17 | 74306868 | 74350279 | 21.3 | 0.385 | 574 |
| MYBL2 | 20 | 42295709 | 42345122 | 21.2 | 0.384 | 574 |
| CENPL | 1 | 173768688 | 173793777 | 21.2 | 0.384 | 574 |
| CDC20 | 1 | 43824626 | 43828873 | 21.1 | 0.384 | 574 |
| PRIM1 | 12 | 57125364 | 57146146 | 21.1 | 0.384 | 574 |
| SMC2 | 9 | 106856541 | 106903700 | 21 | 0.382 | 574 |
| METTL2B | 7 | 128116783 | 128142978 | 21 | 0.382 | 574 |
| TUBD1 | 17 | 57936841 | 57970306 | 20.9 | 0.382 | 574 |
| CLU | 8 | 27454434 | 27472328 | 20.8 | -0.381 | 574 |
| SUPT4H1 | 17 | 56422539 | 56429563 | 20.7 | 0.38 | 574 |
| MASTL | 10 | 27443753 | 27475848 | 20.7 | 0.38 | 574 |
| H2AFX | 11 | 118964585 | 118966177 | 20.7 | 0.38 | 574 |
| CENPN | 16 | 81040103 | 81066709 | 20.5 | 0.378 | 574 |
| LIN54 | 4 | 83845756 | 83934040 | 20.4 | 0.377 | 574 |
| TTF2 | 1 | 117602949 | 117645491 | 20.4 | 0.377 | 574 |
| C16ORF59 | 16 | 2510115 | 2514964 | 20.2 | 0.375 | 574 |
| SRSF12 | 6 | 89805678 | 89827800 | 20.1 | 0.374 | 574 |
| HAUS8 | 19 | 17160571 | 17186343 | 20.1 | 0.375 | 574 |
| SENP5 | 3 | 196594727 | 196661584 | 19.9 | 0.372 | 574 |
| CHRNA5 | 15 | 78857862 | 78887611 | 19.9 | 0.373 | 574 |
| KLHL11 | 17 | 40009799 | 40021629 | 19.7 | 0.37 | 574 |
| TPT1 | 13 | 45911304 | 45915318 | 19.7 | -0.37 | 574 |
| FAM83D | 20 | 37554955 | 37581703 | 19.5 | 0.369 | 574 |
| CEP76 | 18 | 12661955 | 12702703 | 19.5 | 0.369 | 574 |
| RTKN2 | 10 | 63942794 | 64028466 | 19.3 | 0.367 | 574 |
| MAD2L1 | 4 | 120980579 | 120988013 | 19.1 | 0.365 | 574 |
| DGKE | 17 | 54911460 | 54946036 | 19.1 | 0.365 | 574 |
| FAM64A | 17 | 6347735 | 6354385 | 19 | 0.364 | 574 |
| MSH6 | 2 | 48010221 | 48034092 | 18.9 | 0.363 | 574 |
| MRPL27 | 17 | 48445228 | 48450562 | 18.9 | 0.363 | 574 |
| CNTLN | 9 | 17135038 | 17503917 | 18.9 | 0.363 | 574 |
| WDR5 | 9 | 137001210 | 137025094 | 18.9 | 0.363 | 574 |
| SUV39H2 | 10 | 14920782 | 14946304 | 18.9 | 0.364 | 574 |
| GAS2L3 | 12 | 100967489 | 101018685 | 18.8 | 0.362 | 574 |
| CDC27 | 17 | 45195311 | 45266665 | 18.8 | 0.362 | 574 |
| LOC401127 | 4 | 39481875 | 39483523 | 18.8 | 0.363 | 574 |
| SKA3 | 13 | 21727734 | 21750741 | 18.7 | 0.361 | 574 |
| SCAI | 9 | 127704888 | 127905838 | 18.7 | 0.361 | 574 |
| ILF2 | 1 | 153634514 | 153643479 | 18.7 | 0.361 | 574 |
| TRIM37 | 17 | 57060000 | 57184266 | 18.6 | 0.36 | 574 |
| BLM | 15 | 91260579 | 91358686 | 28.3 | 0.441 | 574 |
| BIRC5 | 17 | 76210277 | 76221716 | 31.2 | 0.462 | 574 |
| AURKB | 17 | 8108049 | 8113883 | 22.3 | 0.394 | 574 |
| AURKA | 20 | 54944445 | 54967351 | 24.2 | 0.41 | 574 |
| ATAD5 | 17 | 29159023 | 29222295 | 42.6 | 0.531 | 574 |
| ATAD2 | 8 | 124332091 | 124428590 | 25 | 0.416 | 574 |
| ASPM | 1 | 197053257 | 197115824 | 33.6 | 0.477 | 574 |
| ARHGAP11A | 15 | 32907345 | 32931868 | 33.6 | 0.478 | 574 |
| ANLN | 7 | 36429432 | 36493400 | 23.4 | 0.403 | 574 |
| ANKRD40 | 17 | 48770551 | 48785270 | 26.3 | 0.426 | 574 |
| ANKRD32 | 5 | 93954391 | 94031573 | 21.1 | 0.383 | 574 |
| ALS2CL | 3 | 46710485 | 46735194 | 21.1 | -0.383 | 574 |
| UCEC: Uterine Corpus Endometrial Carcinoma, *: Chromosome, a: chromosome starting site of the gene, b: chromosome ending site of the gene. | | | | | | |

| Table S11. The correlation between BRIP1 and other genes of BRCA from the TCGA database | | | | | | |
| --- | --- | --- | --- | --- | --- | --- |
| Genes | Chr^*^ | Start^a^ | End^b^ | -Log10 (p) | Correlation | Samples |
| PRR11 | 17 | 57232860 | 57284070 | 149.2 | 0.744 | 844 |
| CLSPN | 1 | 36197713 | 36235551 | 137.7 | 0.724 | 844 |
| CKAP2L | 2 | 113495444 | 113522254 | 131.5 | 0.713 | 844 |
| WDHD1 | 14 | 55405656 | 55493819 | 119.5 | 0.689 | 844 |
| POLQ | 3 | 121150273 | 121264853 | 115.5 | 0.681 | 844 |
| CASC5 | 15 | 40886447 | 40954881 | 114.5 | 0.679 | 844 |
| ZNF367 | 9 | 99148225 | 99180669 | 114.4 | 0.678 | 844 |
| RACGAP1 | 12 | 50382945 | 50419307 | 113.3 | 0.676 | 844 |
| RBL1 | 20 | 35626178 | 35724410 | 113.1 | 0.676 | 844 |
| KNTC1 | 12 | 123011809 | 123110947 | 111.7 | 0.672 | 844 |
| DTL | 1 | 212208919 | 212278187 | 110.6 | 0.67 | 844 |
| WDR76 | 15 | 44119112 | 44160617 | 110.3 | 0.669 | 844 |
| KIF11 | 10 | 94352825 | 94415152 | 109.2 | 0.667 | 844 |
| TMEM194A | 12 | 57449426 | 57472574 | 108.5 | 0.665 | 844 |
| ECT2 | 3 | 172468532 | 172539263 | 107.9 | 0.664 | 844 |
| GAS2L3 | 12 | 100967489 | 101018685 | 107.6 | 0.663 | 844 |
| CENPI | X | 100353178 | 100417978 | 106.3 | 0.66 | 844 |
| SGOL1 | 3 | 20202085 | 20227724 | 104.3 | 0.655 | 844 |
| TOP2A | 17 | 38544773 | 38574202 | 104.1 | 0.655 | 844 |
| KPNA2 | 17 | 66031848 | 66042970 | 103.1 | 0.653 | 844 |
| FANCI | 15 | 89787194 | 89860362 | 102.9 | 0.652 | 844 |
| TIMELESS | 12 | 56810157 | 56843200 | 102.3 | 0.651 | 844 |
| CENPE | 4 | 104026963 | 104119566 | 101.7 | 0.649 | 844 |
| BUB1B | 15 | 40453210 | 40513337 | 101.4 | 0.648 | 844 |
| STIL | 1 | 47715811 | 47779819 | 101 | 0.647 | 844 |
| MKI67 | 10 | 129894925 | 129924468 | 100.7 | 0.647 | 844 |
| TMPO | 12 | 98909351 | 98944157 | 99.1 | 0.643 | 844 |
| BUB1 | 2 | 111395409 | 111435684 | 98.8 | 0.642 | 844 |
| NCAPG2 | 7 | 158424003 | 158497520 | 98.4 | 0.641 | 844 |
| CENPO | 2 | 25016175 | 25045245 | 98 | 0.64 | 844 |
| ORC1 | 1 | 52838501 | 52870143 | 97.3 | 0.638 | 844 |
| KIF4A | X | 69509879 | 69640774 | 97.1 | 0.638 | 844 |
| ESPL1 | 12 | 53662083 | 53687427 | 97.1 | 0.638 | 844 |
| PRC1 | 15 | 91509268 | 91537804 | 96.4 | 0.636 | 844 |
| KIAA1524 | 3 | 108268718 | 108308491 | 96.1 | 0.635 | 844 |
| KIF23 | 15 | 69706627 | 69740764 | 95.7 | 0.634 | 844 |
| MAD2L1 | 4 | 120980579 | 120988013 | 95.4 | 0.633 | 844 |
| MCM10 | 10 | 13203554 | 13253104 | 94.6 | 0.631 | 844 |
| KIF20B | 10 | 91461367 | 91534700 | 94.4 | 0.631 | 844 |
| MCM6 | 2 | 136597196 | 136634011 | 92.9 | 0.627 | 844 |
| TLK2 | 17 | 60536019 | 60692841 | 92.5 | 0.626 | 844 |
| TOPBP1 | 3 | 133319449 | 133380737 | 91.9 | 0.624 | 844 |
| KIF14 | 1 | 200520625 | 200589862 | 91.3 | 0.623 | 844 |
| EXO1 | 1 | 242011493 | 242053241 | 90.9 | 0.622 | 844 |
| NUSAP1 | 15 | 41624892 | 41673248 | 90.4 | 0.62 | 844 |
| CCNA2 | 4 | 122737599 | 122745088 | 90.2 | 0.619 | 844 |
| E2F7 | 12 | 77415026 | 77459360 | 90.1 | 0.619 | 844 |
| NOL11 | 17 | 65714061 | 65740266 | 90 | 0.619 | 844 |
| INTS2 | 17 | 59942728 | 60005377 | 88.9 | 0.616 | 844 |
| DEPDC1B | 5 | 59892739 | 59995993 | 88.9 | 0.616 | 844 |
| CDC6 | 17 | 38444146 | 38459413 | 88.7 | 0.616 | 844 |
| E2F8 | 11 | 19245610 | 19263167 | 88.5 | 0.615 | 844 |
| ESCO2 | 8 | 27632058 | 27670141 | 87.1 | 0.611 | 844 |
| SPAG5 | 17 | 26904583 | 26941211 | 86.7 | 0.61 | 844 |
| GSG2 | 17 | 3627197 | 3629992 | 86.6 | 0.61 | 844 |
| NCAPG | 4 | 17812525 | 17846487 | 86.5 | 0.609 | 844 |
| TPX2 | 20 | 30326904 | 30389603 | 86.1 | 0.608 | 844 |
| KIF15 | 3 | 44803209 | 44894748 | 85.6 | 0.607 | 844 |
| CIT | 12 | 120123595 | 120315095 | 85.5 | 0.606 | 844 |
| PARPBP | 12 | 102513956 | 102591298 | 85.5 | 0.607 | 844 |
| FAM111B | 11 | 58874658 | 58894888 | 85.3 | 0.606 | 844 |
| SMC2 | 9 | 106856541 | 106903700 | 85.1 | 0.605 | 844 |
| DCAF7 | 17 | 61627822 | 61671642 | 85.1 | 0.605 | 844 |
| TCF19 | 6 | 31126303 | 31131992 | 85.1 | 0.605 | 844 |
| MCM2 | 3 | 127317253 | 127341278 | 85 | 0.605 | 844 |
| NEIL3 | 4 | 178230991 | 178284092 | 84.7 | 0.604 | 844 |
| PLK4 | 4 | 128802016 | 128820377 | 84.6 | 0.604 | 844 |
| C15ORF42 | 15 | 90118818 | 90171253 | 84.5 | 0.604 | 844 |
| NCAPH | 2 | 97001484 | 97041274 | 84.4 | 0.603 | 844 |
| DEPDC1 | 1 | 68939835 | 68962799 | 84.2 | 0.603 | 844 |
| MSH6 | 2 | 48010221 | 48034092 | 84.1 | 0.602 | 844 |
| RAD51AP1 | 12 | 4647950 | 4669213 | 83.9 | 0.602 | 844 |
| HELLS | 10 | 96305543 | 96361856 | 82.7 | 0.598 | 844 |
| CENPF | 1 | 214776532 | 214837914 | 82.3 | 0.597 | 844 |
| CCNE2 | 8 | 95892453 | 95907482 | 82.3 | 0.597 | 844 |
| FANCB | X | 14861529 | 14891184 | 82.3 | 0.597 | 844 |
| KIF18A | 11 | 28042163 | 28129746 | 81.8 | 0.596 | 844 |
| FOXM1 | 12 | 2966847 | 2986321 | 81.5 | 0.595 | 844 |
| RRM2 | 2 | 10262695 | 10271546 | 81 | 0.593 | 844 |
| SGOL2 | 2 | 201390865 | 201448818 | 80.7 | 0.592 | 844 |
| UHRF1 | 19 | 4909510 | 4962165 | 80.1 | 0.591 | 844 |
| METTL2A | 17 | 60501246 | 60527454 | 80 | 0.591 | 844 |
| KIF18B | 17 | 43002082 | 43025082 | 79.6 | 0.589 | 844 |
| DIAPH3 | 13 | 60239723 | 60738119 | 79 | 0.587 | 844 |
| SMC4 | 3 | 160117430 | 160152741 | 79 | 0.587 | 844 |
| CEP55 | 10 | 95256369 | 95288849 | 78.7 | 0.586 | 844 |
| TRIM37 | 17 | 57060000 | 57184266 | 78.5 | 0.586 | 844 |
| DLGAP5 | 14 | 55614834 | 55658396 | 78 | 0.584 | 844 |
| LMNB1 | 5 | 126112315 | 126172712 | 77.8 | 0.584 | 844 |
| FANCD2 | 3 | 10068113 | 10143614 | 77.2 | 0.582 | 844 |
| PRIM1 | 12 | 57125364 | 57146146 | 76.9 | 0.581 | 844 |
| FAM83D | 20 | 37554955 | 37581703 | 76.7 | 0.58 | 844 |
| WDR62 | 19 | 36545783 | 36596012 | 75.4 | 0.576 | 844 |
| SMC1A | X | 53401070 | 53449618 | 75.1 | 0.575 | 844 |
| HMMR | 5 | 162887517 | 162918953 | 75.1 | 0.575 | 844 |
| IQGAP3 | 1 | 156495197 | 156542396 | 75 | 0.575 | 844 |
| CDCA5 | 11 | 64844927 | 64851615 | 74.4 | 0.573 | 844 |
| MED13 | 17 | 60019966 | 60142643 | 74.3 | 0.573 | 844 |
| MCM8 | 20 | 5931298 | 5975831 | 73.8 | 0.571 | 844 |
| RAD54L | 1 | 46713367 | 46744145 | 73.3 | 0.569 | 844 |
| CDC45 | 22 | 19467414 | 19508135 | 73.2 | 0.569 | 844 |
| DSN1 | 20 | 35380194 | 35402230 | 73.1 | 0.569 | 844 |
| EME1 | 17 | 48450581 | 48458820 | 72.7 | 0.568 | 844 |
| GTSE1 | 22 | 46692638 | 46726707 | 72.4 | 0.567 | 844 |
| TYMS | 18 | 657604 | 673499 | 72.1 | 0.566 | 844 |
| DNA2 | 10 | 70173821 | 70231879 | 72 | 0.565 | 844 |
| CENPK | 5 | 64813593 | 64858995 | 70.9 | 0.562 | 844 |
| CKAP5 | 11 | 46765084 | 46867859 | 70.8 | 0.561 | 844 |
| ERCC6L | X | 71424507 | 71458858 | 70.6 | 0.56 | 844 |
| SKA1 | 18 | 47901392 | 47920538 | 70.1 | 0.559 | 844 |
| PBK | 8 | 27667138 | 27695572 | 70 | 0.559 | 844 |
| NUP155 | 5 | 37291941 | 37371197 | 70 | 0.559 | 844 |
| FBXO5 | 6 | 153291658 | 153304740 | 69.4 | 0.556 | 844 |
| CCNB2 | 15 | 59397284 | 59417244 | 69.3 | 0.556 | 844 |
| XRCC2 | 7 | 152343587 | 152373250 | 69.2 | 0.556 | 844 |
| KIF2C | 1 | 45205490 | 45233438 | 69.1 | 0.555 | 844 |
| E2F2 | 1 | 23832920 | 23857712 | 68.9 | 0.555 | 844 |
| CDC25C | 5 | 137620959 | 137674044 | 68.8 | 0.555 | 844 |
| SKA2 | 17 | 57187308 | 57232800 | 68.5 | 0.553 | 844 |
| MPHOSPH9 | 12 | 123640946 | 123717658 | 68.3 | 0.553 | 844 |
| CHEK1 | 11 | 125495031 | 125546150 | 68.2 | 0.552 | 844 |
| FANCA | 16 | 89803959 | 89883065 | 67.8 | 0.551 | 844 |
| PSMD12 | 17 | 65336619 | 65362721 | 67.7 | 0.551 | 844 |
| CDK2 | 12 | 56360556 | 56366568 | 67.7 | 0.551 | 844 |
| CDC7 | 1 | 91966404 | 91991321 | 67.4 | 0.55 | 844 |
| SMG8 | 17 | 57287371 | 57292611 | 67.3 | 0.549 | 844 |
| PLK1 | 16 | 23690201 | 23701688 | 67.3 | 0.549 | 844 |
| ZWINT | 10 | 58117199 | 58121034 | 66.5 | 0.547 | 844 |
| NEK2 | 1 | 211831599 | 211848972 | 66.5 | 0.547 | 844 |
| CDCA2 | 8 | 25316513 | 25365425 | 66.4 | 0.546 | 844 |
| FEN1 | 11 | 61560109 | 61564714 | 66.3 | 0.546 | 844 |
| SPC24 | 19 | 11257831 | 11266484 | 65.9 | 0.544 | 844 |
| SHCBP1 | 16 | 46614468 | 46655311 | 65.9 | 0.545 | 844 |
| CDC25A | 3 | 48198668 | 48229801 | 65.7 | 0.544 | 844 |
| E2F1 | 20 | 32263292 | 32274210 | 65.4 | 0.543 | 844 |
| INCENP | 11 | 61891445 | 61920635 | 65.4 | 0.543 | 844 |
| KIF20A | 5 | 137514417 | 137523404 | 65 | 0.541 | 844 |
| HJURP | 2 | 234745486 | 234763212 | 65 | 0.541 | 844 |
| CEP152 | 15 | 49030135 | 49103343 | 65 | 0.541 | 844 |
| GINS1 | 20 | 25388323 | 25429191 | 64.7 | 0.54 | 844 |
| RAD51 | 15 | 40987327 | 41024356 | 64.7 | 0.54 | 844 |
| MCM4 | 8 | 48872763 | 48890719 | 64.3 | 0.539 | 844 |
| CDCA8 | 1 | 38158159 | 38175391 | 64.3 | 0.539 | 844 |
| MELK | 9 | 36572905 | 36677679 | 64.1 | 0.538 | 844 |
| CDK1 | 10 | 62538089 | 62554610 | 63.9 | 0.537 | 844 |
| DHX9 | 1 | 182808439 | 182857117 | 63.8 | 0.537 | 844 |
| TRIP13 | 5 | 892969 | 918164 | 63.3 | 0.535 | 844 |
| POLA1 | X | 24712058 | 25015102 | 62.9 | 0.534 | 844 |
| NUP205 | 7 | 135242662 | 135333499 | 62.6 | 0.533 | 844 |
| CHAF1B | 21 | 37757689 | 37789125 | 62.3 | 0.532 | 844 |
| DONSON | 21 | 34947783 | 35288158 | 62.2 | 0.531 | 844 |
| XPO1 | 2 | 61705069 | 61765418 | 62 | 0.531 | 844 |
| MTMR4 | 17 | 56566893 | 56595251 | 61.9 | 0.53 | 844 |
| RRM1 | 11 | 4115924 | 4223759 | 61.8 | 0.53 | 844 |
| TNFSF12 | 17 | 7452374 | 7461207 | 61.7 | -0.53 | 844 |
| EZH2 | 7 | 148504464 | 148581441 | 61.4 | 0.528 | 844 |
| KIFC1 | 6 | 33359313 | 33377699 | 61.3 | 0.528 | 844 |
| TTK | 6 | 80714322 | 80752244 | 61.1 | 0.527 | 844 |
| MMS22L | 6 | 97590037 | 97731061 | 61.1 | 0.527 | 844 |
| SPC25 | 2 | 169727401 | 169746944 | 60.9 | 0.526 | 844 |
| USP1 | 1 | 62901975 | 62917475 | 60.8 | 0.526 | 844 |
| CENPA | 2 | 27008882 | 27023934 | 60.7 | 0.526 | 844 |
| MASTL | 10 | 27443753 | 27475848 | 60.6 | 0.525 | 844 |
| FBXO45 | 3 | 196295725 | 196315930 | 60.6 | 0.525 | 844 |
| PPM1D | 17 | 58677544 | 58743640 | 60.1 | 0.523 | 844 |
| MLF1IP | 4 | 185615219 | 185655286 | 59.8 | 0.522 | 844 |
| FAM72A | 1 | 206138911 | 206155074 | 59.8 | 0.522 | 844 |
| NUF2 | 1 | 163291723 | 163325553 | 59.7 | 0.522 | 844 |
| MDC1 | 6 | 30667584 | 30685458 | 59.3 | 0.52 | 844 |
| C17ORF53 | 17 | 42219274 | 42239844 | 58.9 | 0.519 | 844 |
| GNA13 | 17 | 63005407 | 63052920 | 58.6 | 0.518 | 844 |
| GINS3 | 16 | 58426298 | 58440048 | 58 | 0.515 | 844 |
| HELZ | 17 | 65066554 | 65241319 | 57.6 | 0.514 | 844 |
| NUP153 | 6 | 17615269 | 17706818 | 57.5 | 0.513 | 844 |
| C4ORF21 | 4 | 113460489 | 113558151 | 57.5 | 0.513 | 844 |
| GEN1 | 2 | 17935162 | 17966632 | 57.2 | 0.512 | 844 |
| RAD54B | 8 | 95384188 | 95487343 | 57.1 | 0.512 | 844 |
| ZWILCH | 15 | 66797431 | 66841822 | 57 | 0.512 | 844 |
| RFWD3 | 16 | 74655297 | 74700779 | 56.6 | 0.51 | 844 |
| RFC5 | 12 | 118454506 | 118470042 | 56.6 | 0.51 | 844 |
| NDC80 | 18 | 2571510 | 2616634 | 56.2 | 0.508 | 844 |
| TUBD1 | 17 | 57936841 | 57970306 | 56.1 | 0.508 | 844 |
| FAM64A | 17 | 6347735 | 6354385 | 56.1 | 0.508 | 844 |
| DSCC1 | 8 | 120846181 | 120868170 | 55.9 | 0.507 | 844 |
| CENPL | 1 | 173768688 | 173793777 | 55.9 | 0.507 | 844 |
| MSH2 | 2 | 47630206 | 47906510 | 55.8 | 0.507 | 844 |
| KIF21A | 12 | 39687030 | 39837192 | 55.8 | 0.507 | 844 |
| BRCA2 | 13 | 32889617 | 32973809 | 70.2 | 0.559 | 844 |
| BPTF | 17 | 65821780 | 65980494 | 62.6 | 0.533 | 844 |
| BLM | 15 | 91260579 | 91358686 | 93 | 0.627 | 844 |
| BIRC5 | 17 | 76210277 | 76221716 | 68 | 0.552 | 844 |
| BARD1 | 2 | 215593275 | 215674428 | 71.4 | 0.563 | 844 |
| AURKA | 20 | 54944445 | 54967351 | 67.1 | 0.549 | 844 |
| ATAD5 | 17 | 29159023 | 29222295 | 90.4 | 0.62 | 844 |
| ATAD2 | 8 | 124332091 | 124428590 | 74.9 | 0.575 | 844 |
| ASPM | 1 | 197053257 | 197115824 | 102.6 | 0.651 | 844 |
| ASF1B | 19 | 14230321 | 14247440 | 59 | 0.519 | 844 |
| ARHGAP11B | 15 | 30916697 | 30977810 | 112.7 | 0.675 | 844 |
| ARHGAP11A | 15 | 32907345 | 32931868 | 118.5 | 0.687 | 844 |
| ANLN | 7 | 36429432 | 36493400 | 69.2 | 0.556 | 844 |
| BRCA: Breast invasive carcinoma, *: Chromosome, a: chromosome starting site of the gene, b: chromosome ending site of the gene. | | | | | | |

| Table S12. The correlation between BRIP1 and other genes of COAD, READ from the TCGA database | | | | | | |
| --- | --- | --- | --- | --- | --- | --- |
| Genes | Chr^*^ | Start^a^ | End^b^ | -Log10 (p) | Correlation | Samples |
| INTS2 | 17 | 57360159 | 57360159 | 58.9 | 0.793 | 270 |
| SUZ12 | 17 | 27288378 | 27350135 | 55.5 | 0.779 | 270 |
| KNTC1 | 12 | 121580563 | 121676728 | 54.3 | 0.774 | 270 |
| CDC27 | 17 | 42553299 | 42621537 | 54.1 | 0.773 | 270 |
| C4orf21 | 4 | 113680151 | 113773781 | 53.4 | 0.769 | 270 |
| KPNB1 | 17 | 43082609 | 43114769 | 52.9 | 0.767 | 270 |
| RPS6KB1 | 17 | 55325327 | 55378931 | 51.8 | 0.762 | 270 |
| SASS6 | 1 | 100323471 | 100370958 | 51.2 | 0.759 | 270 |
| GEN1 | 2 | 17804691 | 17826687 | 50.8 | 0.757 | 270 |
| MPHOSPH9 | 12 | 122207306 | 122272287 | 50.7 | 0.757 | 270 |
| PLK4 | 4 | 129022528 | 129039146 | 50.5 | 0.756 | 270 |
| RIF1 | 2 | 151975183 | 152039831 | 48.9 | 0.748 | 270 |
| CEP78 | 9 | 80041086 | 80071498 | 48.8 | 0.748 | 270 |
| ZNF367 | 9 | 98190339 | 98220135 | 47.9 | 0.743 | 270 |
| PDS5A | 4 | 39576370 | 39654592 | 47.7 | 0.742 | 270 |
| CDC7 | 1 | 91739861 | 91762580 | 47.3 | 0.74 | 270 |
| DTL | 1 | 210275855 | 210342905 | 46.9 | 0.738 | 270 |
| MED13 | 17 | 57378610 | 57497348 | 46.2 | 0.734 | 270 |
| C6orf167 | 6 | 97701402 | 97837074 | 46.1 | 0.733 | 270 |
| HAUS6 | 9 | 19046340 | 19092649 | 45.8 | 0.732 | 270 |
| E2F7 | 12 | 75941925 | 75982546 | 45.7 | 0.731 | 270 |
| KIF11 | 10 | 94343112 | 94403533 | 45.6 | 0.731 | 270 |
| TRIM37 | 17 | 54415060 | 54538604 | 45.5 | 0.73 | 270 |
| CLTC | 17 | 55052274 | 55125995 | 45.5 | 0.73 | 270 |
| SMC2 | 9 | 105897486 | 105941417 | 45 | 0.727 | 270 |
| CCDC99 | 5 | 168947998 | 168963789 | 44.8 | 0.726 | 270 |
| TOP2A | 17 | 35799296 | 35827569 | 44.7 | 0.726 | 270 |
| FANCI | 15 | 87591882 | 87660694 | 44.7 | 0.725 | 270 |
| XPO1 | 2 | 61559458 | 61614536 | 44.6 | 0.725 | 270 |
| POLQ | 3 | 122633840 | 122747414 | 44.5 | 0.724 | 270 |
| NEDD1 | 12 | 95825520 | 95869962 | 44.4 | 0.724 | 270 |
| CASC5 | 15 | 38682421 | 38741678 | 44.4 | 0.724 | 270 |
| TMEM194A | 12 | 55739928 | 55758795 | 44.3 | 0.724 | 270 |
| PSIP1 | 9 | 15455517 | 15500186 | 44.1 | 0.722 | 270 |
| BUB1B | 15 | 38240713 | 38300252 | 44.1 | 0.723 | 270 |
| NUP50 | 22 | 43942722 | 43959200 | 44 | 0.722 | 270 |
| STIL | 1 | 47489394 | 47548578 | 44 | 0.722 | 270 |
| PPM1D | 17 | 56032557 | 56095695 | 43.7 | 0.72 | 270 |
| CASP8AP2 | 6 | 90613029 | 90640249 | 43.6 | 0.72 | 270 |
| SKA2 | 17 | 54544419 | 54587306 | 43.5 | 0.719 | 270 |
| RFC1 | 4 | 38966775 | 39044256 | 43.4 | 0.718 | 270 |
| CLSPN | 1 | 35974683 | 36008058 | 43.2 | 0.717 | 270 |
| KIAA1524 | 3 | 109752685 | 109790912 | 42.8 | 0.715 | 270 |
| CENPE | 4 | 104246827 | 104338925 | 42.6 | 0.714 | 270 |
| USP1 | 1 | 62678126 | 62689240 | 42.5 | 0.713 | 270 |
| RFX7 | 15 | 54172834 | 54322775 | 42.5 | 0.713 | 270 |
| HELLS | 10 | 96295668 | 96351369 | 42.3 | 0.712 | 270 |
| NCAPG | 4 | 17421798 | 17454146 | 42 | 0.71 | 270 |
| DDX46 | 5 | 134122527 | 134192295 | 42 | 0.71 | 270 |
| SMC4 | 3 | 161601308 | 161634291 | 42 | 0.71 | 270 |
| CNOT6 | 5 | 179888882 | 179933806 | 41.9 | 0.71 | 270 |
| GUF1 | 4 | 44375396 | 44395455 | 41.8 | 0.709 | 270 |
| NOL11 | 17 | 63144525 | 63170437 | 41.6 | 0.708 | 270 |
| EXO1 | 1 | 240080350 | 240119525 | 41.6 | 0.708 | 270 |
| NUFIP2 | 17 | 24615682 | 24645203 | 41.5 | 0.707 | 270 |
| MSH2 | 2 | 47483834 | 47563592 | 41.5 | 0.707 | 270 |
| TLK2 | 17 | 57912218 | 58043658 | 41.4 | 0.707 | 270 |
| CCDC43 | 17 | 40111900 | 40122647 | 41.1 | 0.705 | 270 |
| TNPO1 | 5 | 72148320 | 72236993 | 41 | 0.704 | 270 |
| SFRS12 | 5 | 65490855 | 65510452 | 40.9 | 0.704 | 270 |
| IREB2 | 15 | 76517734 | 76577540 | 40.8 | 0.703 | 270 |
| CDK2 | 12 | 54647059 | 54651676 | 40.7 | 0.702 | 270 |
| TOPBP1 | 3 | 134802783 | 134862661 | 40.7 | 0.703 | 270 |
| NAA25 | 12 | 110951717 | 111030960 | 40.7 | 0.702 | 270 |
| KIF18A | 11 | 27999322 | 28076070 | 40.7 | 0.702 | 270 |
| HELZ | 17 | 62504829 | 62645382 | 40.7 | 0.702 | 270 |
| LOC144438 | 12 | 47372496 | 47372496 | 40.5 | 0.701 | 270 |
| YTHDC2 | 5 | 112877491 | 112956979 | 40.4 | 0.701 | 270 |
| SSX2IP | 1 | 84885703 | 84909490 | 40.3 | 0.699 | 270 |
| CEP152 | 15 | 46817737 | 46885138 | 40.2 | 0.699 | 270 |
| ZNF770 | 15 | 33060851 | 33062927 | 40.1 | 0.699 | 270 |
| EPT1 | 2 | 26422604 | 26465475 | 40.1 | 0.698 | 270 |
| ZBTB11 | 3 | 102852699 | 102878448 | 39.9 | 0.697 | 270 |
| WDHD1 | 14 | 54477957 | 54563255 | 39.9 | 0.697 | 270 |
| LARP4 | 12 | 49141538 | 49141538 | 39.9 | 0.697 | 270 |
| DNA2 | 10 | 69844801 | 69901885 | 39.4 | 0.694 | 270 |
| POLD3 | 11 | 73981351 | 74029459 | 39.3 | 0.694 | 270 |
| PMS1 | 2 | 190364780 | 190450407 | 39.3 | 0.693 | 270 |
| MASTL | 10 | 27484361 | 27515471 | 39.3 | 0.694 | 270 |
| FAR1 | 11 | 13672888 | 13706897 | 39.3 | 0.693 | 270 |
| CCDC18 | 1 | 93418675 | 93516622 | 39.3 | 0.693 | 270 |
| ETAA1 | 2 | 67478084 | 67490674 | 39.2 | 0.693 | 270 |
| CP110 | 16 | 19446704 | 19470094 | 39.2 | 0.693 | 270 |
| CCDC15 | 11 | 124329838 | 124415817 | 39.2 | 0.693 | 270 |
| KIF20B | 10 | 91455031 | 91523785 | 39.2 | 0.693 | 270 |
| MCM6 | 2 | 136314874 | 136350405 | 39.1 | 0.692 | 270 |
| KIF14 | 1 | 198789138 | 198854474 | 39.1 | 0.692 | 270 |
| CHEK1 | 11 | 125001873 | 125030425 | 39.1 | 0.692 | 270 |
| ZCCHC8 | 12 | 121523996 | 121551340 | 39 | 0.691 | 270 |
| NEK4 | 3 | 52720832 | 52779802 | 39 | 0.692 | 270 |
| HAUS3 | 4 | 2203451 | 2212471 | 39 | 0.692 | 270 |
| NUP107 | 12 | 67367112 | 67422509 | 38.8 | 0.69 | 270 |
| PSMD12 | 17 | 62767420 | 62793097 | 38.8 | 0.69 | 270 |
| NUSAP1 | 15 | 39412447 | 39459676 | 38.8 | 0.69 | 270 |
| CCAR1 | 10 | 70152267 | 70221029 | 38.6 | 0.689 | 270 |
| MDM1 | 12 | 67006532 | 67012292 | 38.6 | 0.689 | 270 |
| FANCM | 14 | 44674984 | 44738961 | 38.6 | 0.689 | 270 |
| OTUD4 | 4 | 146300100 | 146315310 | 38.5 | 0.688 | 270 |
| NUP155 | 5 | 37327758 | 37406836 | 38.5 | 0.688 | 270 |
| ZWILCH | 15 | 64628876 | 64628876 | 38.4 | 0.688 | 270 |
| SLC4A7 | 3 | 27393258 | 27473178 | 38.3 | 0.687 | 270 |
| CAND1 | 12 | 65949764 | 65992877 | 38.3 | 0.687 | 270 |
| ZFP91 | 11 | 58103330 | 58141755 | 38.3 | 0.687 | 270 |
| GTF2H3 | 12 | 122684359 | 122710735 | 38.2 | 0.686 | 270 |
| BUB1 | 2 | 111112012 | 111152043 | 38.2 | 0.686 | 270 |
| CHD1 | 5 | 98219983 | 98289990 | 38.1 | 0.686 | 270 |
| PPAT | 4 | 56956274 | 56996400 | 37.8 | 0.683 | 270 |
| ZNF180 | 19 | 49672458 | 49696132 | 37.6 | 0.682 | 270 |
| KIAA1731 | 11 | 93039521 | 93103083 | 37.5 | 0.681 | 270 |
| C10orf18 | 10 | 5802611 | 5845001 | 37.5 | 0.681 | 270 |
| NPAT | 11 | 107534878 | 107598473 | 37.4 | 0.681 | 270 |
| DPP8 | 15 | 63526274 | 63591944 | 37.4 | 0.681 | 270 |
| NPEPPS | 17 | 42963665 | 43054285 | 37.2 | 0.679 | 270 |
| FBXO5 | 6 | 153333990 | 153338414 | 37.2 | 0.68 | 270 |
| TCERG1 | 5 | 145807105 | 145870398 | 37 | 0.678 | 270 |
| C11orf82 | 11 | 82303428 | 82323025 | 36.9 | 0.678 | 270 |
| PHIP | 6 | 79707128 | 79844504 | 36.9 | 0.677 | 270 |
| LRRC8B | 1 | 89820797 | 89831190 | 36.9 | 0.677 | 270 |
| WDR36 | 5 | 110455885 | 110490480 | 36.8 | 0.677 | 270 |
| DCAF17 | 2 | 171999333 | 172045870 | 36.8 | 0.677 | 270 |
| STXBP4 | 17 | 50418579 | 50592271 | 36.7 | 0.676 | 270 |
| UBE3A | 15 | 23135376 | 23201702 | 36.6 | 0.676 | 270 |
| S100PBP | 1 | 33064287 | 33072226 | 36.6 | 0.676 | 270 |
| MSH6 | 2 | 47863876 | 47887503 | 36.6 | 0.675 | 270 |
| MATR3 | 5 | 138657568 | 138692983 | 36.6 | 0.675 | 270 |
| SMC5 | 9 | 72063814 | 72157067 | 36.5 | 0.675 | 270 |
| METTL14 | 4 | 119826137 | 119850905 | 36.5 | 0.675 | 270 |
| DEPDC1 | 1 | 68715202 | 68735270 | 36.5 | 0.675 | 270 |
| CKAP2L | 2 | 113212870 | 113238646 | 36.5 | 0.675 | 270 |
| TRPM7 | 15 | 48641166 | 48766022 | 36.4 | 0.674 | 270 |
| DENND4A | 15 | 63741242 | 63835842 | 36.4 | 0.674 | 270 |
| DCAF16 | 4 | 17414211 | 17414862 | 36.4 | 0.674 | 270 |
| ZMYM1 | 1 | 35332175 | 35353447 | 36.3 | 0.674 | 270 |
| SENP1 | 12 | 46725371 | 46781522 | 36.3 | 0.673 | 270 |
| CDC6 | 17 | 35699198 | 35711779 | 36.3 | 0.674 | 270 |
| TMPO | 12 | 97433776 | 97465767 | 36.2 | 0.673 | 270 |
| RAPGEF6 | 5 | 130799542 | 130998622 | 36.1 | 0.672 | 270 |
| PPP4R2 | 3 | 73128878 | 73197563 | 36.1 | 0.672 | 270 |
| CEP120 | 5 | 122710111 | 122782079 | 36 | 0.671 | 270 |
| C2orf69 | 2 | 200484406 | 200498854 | 36 | 0.671 | 270 |
| RRM1 | 11 | 4072818 | 4116189 | 36 | 0.671 | 270 |
| THUMPD1 | 16 | 20655702 | 20660562 | 35.9 | 0.671 | 270 |
| LRRC58 | 3 | 121532736 | 121550780 | 35.9 | 0.67 | 270 |
| MED1 | 17 | 34817253 | 34860841 | 35.8 | 0.67 | 270 |
| KIF27 | 9 | 85641735 | 85720326 | 35.7 | 0.669 | 270 |
| KIF23 | 15 | 67493858 | 67527200 | 35.7 | 0.669 | 270 |
| PTAR1 | 9 | 71523077 | 71564674 | 35.7 | 0.669 | 270 |
| PKN2 | 1 | 88922851 | 89071719 | 35.7 | 0.669 | 270 |
| MRPL19 | 2 | 75727441 | 75735919 | 35.7 | 0.669 | 270 |
| MCM10 | 10 | 13246208 | 13291316 | 35.7 | 0.669 | 270 |
| SMAD5 | 5 | 135517348 | 135541067 | 35.6 | 0.668 | 270 |
| DIS3L | 15 | 64372988 | 64412704 | 35.6 | 0.668 | 270 |
| CEP97 | 3 | 102926210 | 102967085 | 35.6 | 0.668 | 270 |
| VPS13C | 15 | 59947414 | 60139865 | 35.5 | 0.668 | 270 |
| SR140 | 3 | 144203160 | 144257982 | 35.5 | 0.668 | 270 |
| HPS5 | 11 | 18258004 | 18295981 | 35.5 | 0.667 | 270 |
| BRCA1 | 17 | 38451220 | 38529639 | 52.9 | 0.767 | 270 |
| BLM | 15 | 89091626 | 89159513 | 43.2 | 0.717 | 270 |
| BCLAF1 | 6 | 136623940 | 136642697 | 41.8 | 0.709 | 270 |
| ATAD5 | 17 | 26183491 | 26246057 | 64.4 | 0.814 | 270 |
| ASPM | 1 | 195320076 | 195382190 | 41.1 | 0.705 | 270 |
| ARHGAP11B | 15 | 28706315 | 28715125 | 36.3 | 0.674 | 270 |
| ARHGAP11A | 15 | 30695704 | 30717338 | 46.5 | 0.736 | 270 |
| APPBP2 | 17 | 55879723 | 55958074 | 38.4 | 0.688 | 270 |
| ANLN | 7 | 36396160 | 36458734 | 37.4 | 0.681 | 270 |
| ANKRD32 | 5 | 93990271 | 94056773 | 48.5 | 0.746 | 270 |
| AGL | 1 | 100099615 | 100159795 | 37.4 | 0.681 | 270 |
| COAD: Colon adenocarcinoma, READ：Rectum adenocarcinoma, *: Chromosome, a: chromosome starting site of the gene, b: chromosome ending site of the gene. | | | | | | |

| Table S13. The correlation between BRIP1 and other genes of ESCA with STAD from the TCGA database | | | | | | |
| --- | --- | --- | --- | --- | --- | --- |
| Genes | Chr^*^ | Start^a^ | End^b^ | -Log10 (p) | Correlation | Samples |
| WDHD1 | 14 | 55405656 | 55493819 | 98 | 0.74 | 558 |
| E2F7 | 12 | 77415026 | 77459360 | 97.7 | 0.74 | 558 |
| INTS2 | 17 | 59942728 | 60005377 | 91.5 | 0.73 | 558 |
| CIT | 12 | 120123595 | 120315095 | 84.4 | 0.71 | 558 |
| POLQ | 3 | 121150273 | 121264853 | 84.2 | 0.7 | 558 |
| DTL | 1 | 212208919 | 212278187 | 83.3 | 0.7 | 558 |
| CASC5 | 15 | 40886447 | 40954881 | 82.4 | 0.7 | 558 |
| FANCI | 15 | 89787194 | 89860362 | 80.7 | 0.69 | 558 |
| HELLS | 10 | 96305543 | 96361856 | 79.3 | 0.69 | 558 |
| RBL1 | 20 | 35626178 | 35724410 | 79.2 | 0.69 | 558 |
| MPHOSPH9 | 12 | 123640946 | 123717658 | 78.3 | 0.69 | 558 |
| KNTC1 | 12 | 123011809 | 123110947 | 77.4 | 0.68 | 558 |
| ORC1 | 1 | 52838501 | 52870143 | 76 | 0.68 | 558 |
| DNMT1 | 19 | 10244022 | 10305755 | 76 | 0.68 | 558 |
| RFWD3 | 16 | 74655297 | 74700779 | 73.5 | 0.67 | 558 |
| CENPI | X | 100353178 | 100417978 | 73.5 | 0.67 | 558 |
| TOPBP1 | 3 | 133319449 | 133380737 | 73.4 | 0.67 | 558 |
| C15orf42 | 15 | 90118818 | 90171253 | 73.2 | 0.67 | 558 |
| KIF11 | 10 | 94352825 | 94415152 | 72.9 | 0.67 | 558 |
| RRM1 | 11 | 4115924 | 4223759 | 71.7 | 0.66 | 558 |
| TIMELESS | 12 | 56810157 | 56843200 | 71.1 | 0.66 | 558 |
| DHX9 | 1 | 182808439 | 182857117 | 70.7 | 0.66 | 558 |
| CENPF | 1 | 214776532 | 214837914 | 70.3 | 0.66 | 558 |
| KIF14 | 1 | 200520625 | 200589862 | 69.6 | 0.66 | 558 |
| SMC2 | 9 | 106856541 | 106903700 | 68.8 | 0.65 | 558 |
| MMS22L | 6 | 97590037 | 97731061 | 68.7 | 0.65 | 558 |
| UHRF1 | 19 | 4909510 | 4962165 | 68.4 | 0.65 | 558 |
| EXO1 | 1 | 242011493 | 242053241 | 68 | 0.65 | 558 |
| TTF2 | 1 | 117602949 | 117645491 | 67.9 | 0.65 | 558 |
| PLK4 | 4 | 128802016 | 128820377 | 67.8 | 0.65 | 558 |
| DNA2 | 10 | 70173821 | 70231879 | 67.5 | 0.65 | 558 |
| FANCM | 14 | 45605136 | 45670093 | 67.4 | 0.65 | 558 |
| KIF15 | 3 | 44803209 | 44894748 | 66.5 | 0.65 | 558 |
| CDC7 | 1 | 91966404 | 91991321 | 66.5 | 0.65 | 558 |
| KIF23 | 15 | 69706627 | 69740764 | 66.3 | 0.65 | 558 |
| KIF18B | 17 | 43002082 | 43025082 | 64.9 | 0.64 | 558 |
| MSH6 | 2 | 48010221 | 48034092 | 64.5 | 0.64 | 558 |
| BUB1B | 15 | 40453210 | 40513337 | 64.5 | 0.64 | 558 |
| POLE | 12 | 133200348 | 133263945 | 64.4 | 0.64 | 558 |
| CEP78 | 9 | 80850991 | 80886799 | 64.4 | 0.64 | 558 |
| NCAPG2 | 7 | 158424003 | 158497520 | 64 | 0.64 | 558 |
| C4orf21 | 4 | 113460489 | 113558151 | 64 | 0.64 | 558 |
| NUP205 | 7 | 135242662 | 135333499 | 63.7 | 0.64 | 558 |
| NOL11 | 17 | 65714061 | 65740266 | 63.6 | 0.63 | 558 |
| STIL | 1 | 47715811 | 47779819 | 63.4 | 0.63 | 558 |
| CEP152 | 15 | 49030135 | 49103343 | 63.4 | 0.63 | 558 |
| XRCC2 | 7 | 152343587 | 152373250 | 63 | 0.63 | 558 |
| CEP85 | 1 | 26560693 | 26605299 | 62.8 | 0.63 | 558 |
| CLSPN | 1 | 36197713 | 36235551 | 62.1 | 0.63 | 558 |
| MCM2 | 3 | 127317253 | 127341278 | 61.3 | 0.63 | 558 |
| RAD54L | 1 | 46713367 | 46744145 | 61.1 | 0.63 | 558 |
| NCAPH | 2 | 97001484 | 97041274 | 60.5 | 0.62 | 558 |
| GINS3 | 16 | 58426298 | 58440048 | 60.3 | 0.62 | 558 |
| ZNF367 | 9 | 99148225 | 99180669 | 60 | 0.62 | 558 |
| MCM6 | 2 | 136597196 | 136634011 | 59.6 | 0.62 | 558 |
| MSH2 | 2 | 47630206 | 47906510 | 59.3 | 0.62 | 558 |
| KIF4A | X | 69509879 | 69640774 | 59 | 0.62 | 558 |
| RFC5 | 12 | 118454506 | 118470042 | 58.8 | 0.62 | 558 |
| MKI67 | 10 | 129894925 | 129924468 | 58.8 | 0.62 | 558 |
| FANCD2 | 3 | 10068113 | 10143614 | 58.7 | 0.62 | 558 |
| FANCB | X | 14861529 | 14891184 | 58.7 | 0.62 | 558 |
| WDR76 | 15 | 44119112 | 44160617 | 58.4 | 0.61 | 558 |
| SPAG5 | 17 | 26904583 | 26941211 | 57.9 | 0.61 | 558 |
| MCM4 | 8 | 48872763 | 48890719 | 57.9 | 0.61 | 558 |
| NEDD1 | 12 | 97301001 | 97347469 | 57.8 | 0.61 | 558 |
| ERCC6L | X | 71424507 | 71458858 | 57.8 | 0.61 | 558 |
| KIF4B | 5 | 154393260 | 154397685 | 57.7 | 0.61 | 558 |
| CENPE | 4 | 104026963 | 104119566 | 57.4 | 0.61 | 558 |
| RRAS | 19 | 50138552 | 50143400 | 57.3 | -0.61 | 558 |
| USP1 | 1 | 62901975 | 62917475 | 57.3 | 0.61 | 558 |
| CKAP2L | 2 | 113495444 | 113522254 | 56.6 | 0.61 | 558 |
| C11orf82 | 11 | 82612737 | 82669319 | 56.6 | 0.61 | 558 |
| RIF1 | 2 | 152266397 | 152364527 | 56.4 | 0.61 | 558 |
| PRKDC | 8 | 48685669 | 48872743 | 55.9 | 0.6 | 558 |
| SUZ12 | 17 | 30264044 | 30328057 | 55.8 | 0.6 | 558 |
| XPO1 | 2 | 61705069 | 61765418 | 55.7 | 0.6 | 558 |
| MED13 | 17 | 60019966 | 60142643 | 55.4 | 0.6 | 558 |
| SMC4 | 3 | 160117430 | 160152741 | 55.3 | 0.6 | 558 |
| KIF18A | 11 | 28042163 | 28129746 | 55.3 | 0.6 | 558 |
| DLGAP5 | 14 | 55614834 | 55658396 | 55.3 | 0.6 | 558 |
| RAD51AP1 | 12 | 4647950 | 4669213 | 55.2 | 0.6 | 558 |
| KIF24 | 9 | 34211974 | 34329198 | 55.2 | 0.6 | 558 |
| PRC1 | 15 | 91509268 | 91537804 | 54.9 | 0.6 | 558 |
| KIAA1524 | 3 | 108268718 | 108308491 | 54.8 | 0.6 | 558 |
| GSG2 | 17 | 3627197 | 3629992 | 54.6 | 0.6 | 558 |
| TOP2A | 17 | 38544773 | 38574202 | 54.5 | 0.6 | 558 |
| NCAPD2 | 12 | 6603298 | 6641132 | 54.5 | 0.6 | 558 |
| RACGAP1 | 12 | 50382945 | 50419307 | 54.4 | 0.6 | 558 |
| CCDC138 | 2 | 109403219 | 109492847 | 54.4 | 0.6 | 558 |
| CAND1 | 12 | 67663061 | 67708388 | 54.3 | 0.6 | 558 |
| MCM10 | 10 | 13203554 | 13253104 | 54.2 | 0.6 | 558 |
| DEPDC1 | 1 | 68939835 | 68962799 | 54.2 | 0.6 | 558 |
| CCNE2 | 8 | 95892453 | 95907482 | 54.1 | 0.6 | 558 |
| GEN1 | 2 | 17935162 | 17966632 | 53.9 | 0.59 | 558 |
| CDC45 | 22 | 19467414 | 19508135 | 53.8 | 0.59 | 558 |
| SGOL2 | 2 | 201390865 | 201448818 | 53.7 | 0.59 | 558 |
| BUB1 | 2 | 111395409 | 111435684 | 53.6 | 0.59 | 558 |
| KPNB1 | 17 | 45727275 | 45761004 | 53.5 | 0.59 | 558 |
| CKAP5 | 11 | 46765084 | 46867859 | 53.5 | 0.59 | 558 |
| CCDC107 | 9 | 35658287 | 35661500 | 53.4 | -0.59 | 558 |
| PRIM1 | 12 | 57125364 | 57146146 | 53.1 | 0.59 | 558 |
| RMI1 | 9 | 86595637 | 86618987 | 52.8 | 0.59 | 558 |
| INTS7 | 1 | 212113741 | 212209002 | 52.7 | 0.59 | 558 |
| NCAPD3 | 11 | 134022337 | 134094426 | 52.5 | 0.59 | 558 |
| CDCA2 | 8 | 25316513 | 25365425 | 52.5 | 0.59 | 558 |
| CENPO | 2 | 25016175 | 25045245 | 52.3 | 0.59 | 558 |
| GTF2H3 | 12 | 124118381 | 124145334 | 51.9 | 0.59 | 558 |
| NCAPG | 4 | 17812525 | 17846487 | 51.5 | 0.58 | 558 |
| EPT1 | 2 | 26568954 | 26618759 | 51.5 | 0.58 | 558 |
| CST3 | 20 | 23608534 | 23618574 | 51.2 | -0.58 | 558 |
| UTP20 | 12 | 101673905 | 101780397 | 50.9 | 0.58 | 558 |
| PALB2 | 16 | 23614483 | 23652678 | 50.9 | 0.58 | 558 |
| NUP107 | 12 | 69080731 | 69136473 | 50.6 | 0.58 | 558 |
| RRM2 | 2 | 10262695 | 10271546 | 50.5 | 0.58 | 558 |
| CHAF1B | 21 | 37757689 | 37789125 | 50.5 | 0.58 | 558 |
| GTSE1 | 22 | 46692638 | 46726707 | 50.3 | 0.58 | 558 |
| RBM12 | 20 | 34236847 | 34252878 | 50.2 | 0.58 | 558 |
| SRSF1 | 17 | 56078280 | 56084707 | 49.9 | 0.58 | 558 |
| NUP50 | 22 | 45559726 | 45583890 | 49.8 | 0.58 | 558 |
| PRIM2 | 6 | 57182422 | 57513376 | 49.8 | 0.58 | 558 |
| GMPS | 3 | 155588325 | 155655520 | 49.5 | 0.57 | 558 |
| WHSC1 | 4 | 1873123 | 1983934 | 49.1 | 0.57 | 558 |
| WDR62 | 19 | 36545783 | 36596012 | 49 | 0.57 | 558 |
| ECT2 | 3 | 172468532 | 172539263 | 48.9 | 0.57 | 558 |
| TMEM194A | 12 | 57449426 | 57472574 | 48.8 | 0.57 | 558 |
| SASS6 | 1 | 100549102 | 100598511 | 48.7 | 0.57 | 558 |
| POLA1 | X | 24712058 | 25015102 | 48.5 | 0.57 | 558 |
| EZH2 | 7 | 148504464 | 148581441 | 48.5 | 0.57 | 558 |
| PSMD12 | 17 | 65336619 | 65362721 | 48.4 | 0.57 | 558 |
| FAM111B | 11 | 58874658 | 58894888 | 48.1 | 0.57 | 558 |
| DCLRE1A | 10 | 115594484 | 115613859 | 47.9 | 0.57 | 558 |
| PARPBP | 12 | 102513956 | 102591298 | 47.8 | 0.57 | 558 |
| NUP160 | 11 | 47799670 | 47870057 | 47.8 | 0.57 | 558 |
| PRR11 | 17 | 57232860 | 57284070 | 47.7 | 0.57 | 558 |
| SHCBP1 | 16 | 46614468 | 46655311 | 47.4 | 0.56 | 558 |
| NUP155 | 5 | 37291941 | 37371197 | 47.4 | 0.56 | 558 |
| KIF20B | 10 | 91461367 | 91534700 | 47.4 | 0.56 | 558 |
| ORC6 | 16 | 46723558 | 46732306 | 47.1 | 0.56 | 558 |
| ESPL1 | 12 | 53662083 | 53687427 | 47.1 | 0.56 | 558 |
| TRIM37 | 17 | 57060000 | 57184266 | 47 | 0.56 | 558 |
| MTBP | 8 | 121457666 | 121535875 | 47 | 0.56 | 558 |
| FBXO5 | 6 | 153291658 | 153304740 | 47 | 0.56 | 558 |
| GTF3C4 | 9 | 135545728 | 135565470 | 46.8 | 0.56 | 558 |
| C1orf112 | 1 | 169631245 | 169822229 | 46.8 | 0.56 | 558 |
| MYO19 | 17 | 34851599 | 34891305 | 46.6 | 0.56 | 558 |
| TTK | 6 | 80714322 | 80752244 | 46.5 | 0.56 | 558 |
| TMEM150A | 2 | 85825670 | 85829822 | 46.5 | -0.56 | 558 |
| ZWILCH | 15 | 66797431 | 66841822 | 46.4 | 0.56 | 558 |
| QTRTD1 | 3 | 113775611 | 113807268 | 46.3 | 0.56 | 558 |
| TMEM48 | 1 | 54231134 | 54304225 | 46.2 | 0.56 | 558 |
| KPNA2 | 17 | 66031848 | 66042970 | 46.2 | 0.56 | 558 |
| CDC6 | 17 | 38444146 | 38459413 | 46.2 | 0.56 | 558 |
| MELK | 9 | 36572905 | 36677679 | 46.1 | 0.56 | 558 |
| NUSAP1 | 15 | 41624892 | 41673248 | 46 | 0.56 | 558 |
| C1orf96 | 1 | 229456752 | 229478688 | 46 | 0.56 | 558 |
| NUP188 | 9 | 131683174 | 131769374 | 45.9 | 0.56 | 558 |
| FIGNL1 | 7 | 50511832 | 50518088 | 45.9 | 0.56 | 558 |
| WDR67 | 8 | 124084920 | 124164392 | 45.8 | 0.56 | 558 |
| ESCO2 | 8 | 27632058 | 27670141 | 45.8 | 0.56 | 558 |
| COX7A1 | 19 | 36641824 | 36643771 | 45.8 | -0.56 | 558 |
| C17orf53 | 17 | 42219274 | 42239844 | 45.5 | 0.55 | 558 |
| SUPT16H | 14 | 21819631 | 21852425 | 45.1 | 0.55 | 558 |
| CEP128 | 14 | 80943330 | 81425828 | 44.8 | 0.55 | 558 |
| E2F2 | 1 | 23832920 | 23857712 | 44.5 | 0.55 | 558 |
| INCENP | 11 | 61891445 | 61920635 | 44.3 | 0.55 | 558 |
| G3BP1 | 5 | 151151476 | 151184915 | 44.3 | 0.55 | 558 |
| CHAF1A | 19 | 4402660 | 4443394 | 44.3 | 0.55 | 558 |
| ZNF280C | X | 129336673 | 129402922 | 43.9 | 0.55 | 558 |
| SELM | 22 | 31500763 | 31503551 | 43.8 | -0.55 | 558 |
| LTC4S | 5 | 179220986 | 179223513 | 43.7 | -0.54 | 558 |
| FANCA | 16 | 89803959 | 89883065 | 43.6 | 0.54 | 558 |
| HNRNPU | 1 | 245013602 | 245027827 | 43.6 | 0.54 | 558 |
| CDC27 | 17 | 45195311 | 45266665 | 43.5 | 0.54 | 558 |
| L2HGDH | 14 | 50704285 | 50778947 | 43.5 | 0.54 | 558 |
| TEX10 | 9 | 103064357 | 103115259 | 43.4 | 0.54 | 558 |
| PDS5A | 4 | 39824483 | 39979576 | 43.3 | 0.54 | 558 |
| PPP1R14A | 19 | 38741877 | 38747231 | 43.2 | -0.54 | 558 |
| CTDSPL2 | 15 | 44719579 | 44819429 | 43 | 0.54 | 558 |
| CDK12 | 17 | 37617739 | 37690800 | 43 | 0.54 | 558 |
| HAUS6 | 9 | 19053141 | 19102902 | 42.8 | 0.54 | 558 |
| TUBGCP4 | 15 | 43663313 | 43698240 | 42.6 | 0.54 | 558 |
| PPAT | 4 | 57259529 | 57301802 | 42.5 | 0.54 | 558 |
| CDK1 | 10 | 62538089 | 62554610 | 42.5 | 0.54 | 558 |
| RTTN | 18 | 67671043 | 67872962 | 42.4 | 0.54 | 558 |
| MYL6 | 12 | 56552045 | 56555366 | 42.3 | -0.54 | 558 |
| ILF3 | 19 | 10764937 | 10803095 | 42.3 | 0.54 | 558 |
| R3HDM1 | 2 | 136289083 | 136482839 | 42.1 | 0.54 | 558 |
| BRCA2 | 13 | 32889617 | 32973809 | 61.5 | 0.63 | 558 |
| BRCA1 | 17 | 41196312 | 41322420 | 93.7 | 0.73 | 558 |
| BLM | 15 | 91260579 | 91358686 | 55.7 | 0.6 | 558 |
| BARD1 | 2 | 215593275 | 215674428 | 65.5 | 0.64 | 558 |
| ATAD5 | 17 | 29159023 | 29222295 | 107.9 | 0.77 | 558 |
| ATAD2 | 8 | 124332091 | 124428590 | 63.9 | 0.64 | 558 |
| ASPM | 1 | 197053257 | 197115824 | 71.4 | 0.66 | 558 |
| ARHGAP11B | 15 | 30916697 | 30977810 | 55.1 | 0.6 | 558 |
| ARHGAP11A | 15 | 32907345 | 32931868 | 59.4 | 0.62 | 558 |
| ANLN | 7 | 36429432 | 36493400 | 55.7 | 0.6 | 558 |
| ANKRD32 | 5 | 93954391 | 94031573 | 53.5 | 0.59 | 558 |
| ANAPC1 | 2 | 112523848 | 112642267 | 43.2 | 0.54 | 558 |
| ALG10 | 12 | 34175216 | 34181236 | 48 | 0.57 | 558 |
| ESCA: Esophageal carcinoma, STAD: Stomach adenocarcinoma, *: Chromosome, a: chromosome starting site of the gene, b: chromosome ending site of the gene. | | | | | | |

| Table S14. The correlation between BRIP1 and other genes of GBM from the TCGA database | | | | | | |
| --- | --- | --- | --- | --- | --- | --- |
| Genes | Chr^*^ | Start^a^ | End^b^ | -Log10 (p) | Correlation | Samples |
| MCM10 | 10 | 13203554 | 13253104 | 93.3 | 0.744 | 528 |
| PLK4 | 4 | 128802016 | 128820377 | 76.3 | 0.694 | 528 |
| KIF18A | 11 | 28042163 | 28129746 | 73.4 | 0.684 | 528 |
| NCAPH | 2 | 97001484 | 97041274 | 68.9 | 0.668 | 528 |
| CHEK1 | 11 | 125495031 | 125546150 | 68.7 | 0.668 | 528 |
| TMPO | 12 | 98909351 | 98944157 | 67.8 | 0.664 | 528 |
| BUB1 | 2 | 111395409 | 111435684 | 65.8 | 0.657 | 528 |
| CDC6 | 17 | 38444146 | 38459413 | 65.1 | 0.654 | 528 |
| BUB1B | 15 | 40453210 | 40513337 | 65 | 0.654 | 528 |
| CDCA8 | 1 | 38158159 | 38175391 | 64.6 | 0.652 | 528 |
| ESPL1 | 12 | 53662083 | 53687427 | 64.1 | 0.651 | 528 |
| EXO1 | 1 | 242011493 | 242053241 | 63 | 0.646 | 528 |
| CENPI | X | 100353178 | 100417978 | 62.1 | 0.643 | 528 |
| CENPA | 2 | 27008882 | 27023934 | 61.8 | 0.641 | 528 |
| TPX2 | 20 | 30326904 | 30389603 | 61.7 | 0.641 | 528 |
| KIF15 | 3 | 44803209 | 44894748 | 61.7 | 0.641 | 528 |
| CDK1 | 10 | 62538089 | 62554610 | 61.6 | 0.641 | 528 |
| CCNB2 | 15 | 59397284 | 59417244 | 61.2 | 0.639 | 528 |
| KNTC1 | 12 | 123011809 | 123110947 | 61.1 | 0.639 | 528 |
| KIF23 | 15 | 69706627 | 69740764 | 60.6 | 0.637 | 528 |
| DTL | 1 | 212208919 | 212278187 | 60 | 0.634 | 528 |
| ZWINT | 10 | 58117199 | 58121034 | 59.6 | 0.633 | 528 |
| SMC4 | 3 | 160117430 | 160152741 | 59.2 | 0.631 | 528 |
| DSN1 | 20 | 35380194 | 35402230 | 58.9 | 0.63 | 528 |
| MCM7 | 7 | 99690404 | 99699427 | 58.7 | 0.628 | 528 |
| NEIL3 | 4 | 178230991 | 178284092 | 58.5 | 0.628 | 528 |
| SRSF2 | 17 | 74730197 | 74733493 | 57.7 | 0.624 | 528 |
| KIF2C | 1 | 45205490 | 45233438 | 57.5 | 0.624 | 528 |
| SKP2 | 5 | 36152145 | 36184142 | 57.3 | 0.623 | 528 |
| POLQ | 3 | 121150273 | 121264853 | 57.3 | 0.623 | 528 |
| MLF1IP | 4 | 185615219 | 185655286 | 57.2 | 0.622 | 528 |
| KIAA0101 | 15 | 64657211 | 64673702 | 57.2 | 0.622 | 528 |
| EZH2 | 7 | 148504464 | 148581441 | 57 | 0.622 | 528 |
| FANCI | 15 | 89787194 | 89860362 | 56.8 | 0.62 | 528 |
| LMNB1 | 5 | 126112315 | 126172712 | 56.6 | 0.62 | 528 |
| RFC4 | 3 | 186507682 | 186524484 | 56.5 | 0.619 | 528 |
| KIF11 | 10 | 94352825 | 94415152 | 56.5 | 0.619 | 528 |
| KIF4A | X | 69509879 | 69640774 | 56.2 | 0.618 | 528 |
| NCAPG | 4 | 17812525 | 17846487 | 56 | 0.617 | 528 |
| CENPE | 4 | 104026963 | 104119566 | 55.8 | 0.616 | 528 |
| SKA1 | 18 | 47901392 | 47920538 | 55.7 | 0.616 | 528 |
| CCNB1 | 5 | 68462837 | 68474070 | 55.7 | 0.616 | 528 |
| RFC3 | 13 | 34392206 | 34540695 | 55.1 | 0.613 | 528 |
| RFC5 | 12 | 118454506 | 118470042 | 54.8 | 0.612 | 528 |
| CCNA2 | 4 | 122737599 | 122745088 | 54.7 | 0.611 | 528 |
| ECT2 | 3 | 172468532 | 172539263 | 54.6 | 0.611 | 528 |
| CENPN | 16 | 81040103 | 81066709 | 54.4 | 0.61 | 528 |
| CDC25C | 5 | 137620959 | 137674044 | 54.2 | 0.609 | 528 |
| UBE2C | 20 | 44441255 | 44445596 | 54.1 | 0.609 | 528 |
| NEK2 | 1 | 211831599 | 211848972 | 53.9 | 0.608 | 528 |
| TTK | 6 | 80714322 | 80752244 | 53.7 | 0.607 | 528 |
| RAD51AP1 | 12 | 4647950 | 4669213 | 53.5 | 0.606 | 528 |
| DHX15 | 4 | 24529088 | 24586184 | 53.5 | 0.606 | 528 |
| TRIP13 | 5 | 892969 | 918164 | 52.9 | 0.603 | 528 |
| FEN1 | 11 | 61560109 | 61564714 | 52.8 | 0.603 | 528 |
| PCNA | 20 | 5095599 | 5107268 | 52.7 | 0.602 | 528 |
| TOP2A | 17 | 38544773 | 38574202 | 52.6 | 0.602 | 528 |
| MELK | 9 | 36572905 | 36677679 | 52.3 | 0.6 | 528 |
| MSH6 | 2 | 48010221 | 48034092 | 52.2 | 0.6 | 528 |
| OIP5 | 15 | 41601466 | 41624819 | 52.1 | 0.6 | 528 |
| SPC25 | 2 | 169727401 | 169746944 | 52 | 0.599 | 528 |
| CDC25A | 3 | 48198668 | 48229801 | 51.7 | 0.598 | 528 |
| PRC1 | 15 | 91509268 | 91537804 | 51.6 | 0.597 | 528 |
| MAD2L1 | 4 | 120980579 | 120988013 | 51.6 | 0.597 | 528 |
| PLK1 | 16 | 23690201 | 23701688 | 51.5 | 0.597 | 528 |
| KPNA2 | 17 | 66031848 | 66042970 | 51.5 | 0.597 | 528 |
| DLGAP5 | 14 | 55614834 | 55658396 | 51.5 | 0.597 | 528 |
| CCDC99 | 5 | 169010638 | 169031781 | 51.4 | 0.596 | 528 |
| CDCA3 | 12 | 6953963 | 6960456 | 51.1 | 0.595 | 528 |
| NCAPG2 | 7 | 158424003 | 158497520 | 50.8 | 0.593 | 528 |
| C1ORF135 | 1 | 26160497 | 26185848 | 50.4 | 0.591 | 528 |
| MCM2 | 3 | 127317253 | 127341278 | 50.3 | 0.591 | 528 |
| SPAG5 | 17 | 26904583 | 26941211 | 50.2 | 0.591 | 528 |
| EXOSC9 | 4 | 122722472 | 122738176 | 49.9 | 0.589 | 528 |
| SUV39H2 | 10 | 14920782 | 14946304 | 49.5 | 0.587 | 528 |
| GINS1 | 20 | 25388323 | 25429191 | 49.3 | 0.586 | 528 |
| TIMELESS | 12 | 56810157 | 56843200 | 49.1 | 0.585 | 528 |
| SRSF3 | 6 | 36562090 | 36572244 | 49.1 | 0.585 | 528 |
| RNASEH2A | 19 | 12917428 | 12924462 | 48.8 | 0.584 | 528 |
| KIF14 | 1 | 200520625 | 200589862 | 48.6 | 0.583 | 528 |
| MCM4 | 8 | 48872763 | 48890719 | 48.6 | 0.583 | 528 |
| KIF20A | 5 | 137514417 | 137523404 | 48.5 | 0.582 | 528 |
| ZWILCH | 15 | 66797431 | 66841822 | 48.4 | 0.582 | 528 |
| CDC45 | 22 | 19467414 | 19508135 | 48.4 | 0.582 | 528 |
| GINS2 | 16 | 85711280 | 85722588 | 48.1 | 0.58 | 528 |
| NUSAP1 | 15 | 41624892 | 41673248 | 47.9 | 0.579 | 528 |
| MKI67 | 10 | 129894925 | 129924468 | 47.5 | 0.577 | 528 |
| ILF2 | 1 | 153634514 | 153643479 | 47.3 | 0.576 | 528 |
| CTPS1 | 1 | 41445007 | 41478235 | 47 | 0.575 | 528 |
| HMMR | 5 | 162887517 | 162918953 | 46.9 | 0.574 | 528 |
| CDC20 | 1 | 43824626 | 43828873 | 46.9 | 0.574 | 528 |
| PARPBP | 12 | 102513956 | 102591298 | 46.8 | 0.574 | 528 |
| MCM3 | 6 | 52128812 | 52149582 | 46.8 | 0.574 | 528 |
| CENPF | 1 | 214776532 | 214837914 | 46.7 | 0.573 | 528 |
| POLE | 12 | 133200348 | 133263945 | 46.6 | 0.573 | 528 |
| KIFC1 | 6 | 33359313 | 33377699 | 46.6 | 0.573 | 528 |
| RAD1 | 5 | 34905366 | 34918383 | 46.3 | 0.571 | 528 |
| DBF4 | 7 | 87505544 | 87538856 | 46.2 | 0.571 | 528 |
| CCNE2 | 8 | 95892453 | 95907482 | 45.9 | 0.569 | 528 |
| NDC80 | 18 | 2571510 | 2616634 | 45.6 | 0.567 | 528 |
| SRSF1 | 17 | 56078280 | 56084707 | 45.3 | 0.566 | 528 |
| WDHD1 | 14 | 55405656 | 55493819 | 45.2 | 0.565 | 528 |
| MCM6 | 2 | 136597196 | 136634011 | 45.2 | 0.565 | 528 |
| INTS7 | 1 | 212113741 | 212209002 | 45.2 | 0.565 | 528 |
| SMC2 | 9 | 106856541 | 106903700 | 44.9 | 0.564 | 528 |
| SRPK1 | 6 | 35800811 | 35888957 | 44.8 | 0.563 | 528 |
| FOXM1 | 12 | 2966847 | 2986321 | 44.8 | 0.563 | 528 |
| VRK1 | 14 | 97263684 | 97347951 | 44.6 | 0.562 | 528 |
| SNRPD1 | 18 | 19192260 | 19210208 | 44.6 | 0.562 | 528 |
| TYMS | 18 | 657604 | 673499 | 44.3 | 0.561 | 528 |
| RRM1 | 11 | 4115924 | 4223759 | 44.1 | 0.559 | 528 |
| PBK | 8 | 27667138 | 27695572 | 44.1 | 0.559 | 528 |
| MSH2 | 2 | 47630206 | 47906510 | 43.7 | 0.557 | 528 |
| ORC6 | 16 | 46723558 | 46732306 | 43.5 | 0.556 | 528 |
| HN1L | 16 | 1728278 | 1752073 | 43.5 | 0.556 | 528 |
| MYBL2 | 20 | 42295709 | 42345122 | 43.4 | 0.556 | 528 |
| CCNF | 16 | 2479395 | 2508859 | 43.3 | 0.555 | 528 |
| H2AFZ | 4 | 100869243 | 100871512 | 42.6 | 0.551 | 528 |
| CSE1L | 20 | 47662838 | 47713486 | 42.5 | 0.551 | 528 |
| PHTF2 | 7 | 77428109 | 77586821 | 42.4 | 0.55 | 528 |
| DEPDC1 | 1 | 68939835 | 68962799 | 42.4 | 0.55 | 528 |
| CHAF1A | 19 | 4402660 | 4443394 | 42.3 | 0.55 | 528 |
| NONO | X | 70503042 | 70521018 | 42.2 | 0.549 | 528 |
| RAD51 | 15 | 40987327 | 41024356 | 42 | 0.548 | 528 |
| ERCC6L | X | 71424507 | 71458858 | 42 | 0.548 | 528 |
| TRA2B | 3 | 185632358 | 185655924 | 41.9 | 0.547 | 528 |
| PRIM1 | 12 | 57125364 | 57146146 | 41.9 | 0.548 | 528 |
| TACC3 | 4 | 1723217 | 1746905 | 41.8 | 0.547 | 528 |
| CDC7 | 1 | 91966404 | 91991321 | 41.8 | 0.547 | 528 |
| WDR76 | 15 | 44119112 | 44160617 | 41.6 | 0.546 | 528 |
| STIL | 1 | 47715811 | 47779819 | 41.6 | 0.546 | 528 |
| CDKN3 | 14 | 54863673 | 54886934 | 41.6 | 0.546 | 528 |
| HMGB2 | 4 | 174252527 | 174255595 | 41.2 | 0.544 | 528 |
| POLA2 | 11 | 65029432 | 65065088 | 41 | 0.542 | 528 |
| CKS1B | 1 | 154947118 | 154951725 | 40.9 | 0.542 | 528 |
| XRCC5 | 2 | 216974020 | 217071016 | 40.8 | 0.541 | 528 |
| SRSF10 | 1 | 24291418 | 24306953 | 40.8 | 0.541 | 528 |
| HMGB3P1 | 20 | 33421378 | 33422265 | 40.7 | 0.541 | 528 |
| TROAP | 12 | 49716971 | 49725514 | 39.8 | 0.535 | 528 |
| CEP55 | 10 | 95256369 | 95288849 | 39.7 | 0.535 | 528 |
| RACGAP1 | 12 | 50382945 | 50419307 | 39.5 | 0.534 | 528 |
| BRCA2 | 13 | 32889617 | 32973809 | 48.3 | 0.581 | 528 |
| BORA | 13 | 73302042 | 73330328 | 40.3 | 0.539 | 528 |
| BIRC5 | 17 | 76210277 | 76221716 | 58.6 | 0.628 | 528 |
| AURKB | 17 | 8108049 | 8113883 | 53.3 | 0.605 | 528 |
| AURKA | 20 | 54944445 | 54967351 | 50.2 | 0.591 | 528 |
| ATAD2 | 8 | 124332091 | 124428590 | 68.7 | 0.668 | 528 |
| ASPM | 1 | 197053257 | 197115824 | 56.5 | 0.619 | 528 |
| ASF1B | 19 | 14230321 | 14247440 | 47.8 | 0.578 | 528 |
| ARHGAP11A | 15 | 32907345 | 32931868 | 47 | 0.575 | 528 |
| GBM: Glioblastoma multiforme, *: Chromosome, a: chromosome starting site of the gene, b: chromosome ending site of the gene. | | | | | | |

| Table S15. The correlation between BRIP1 and other genes of KIRC from the TCGA database | | | | | | |
| --- | --- | --- | --- | --- | --- | --- |
| Genes | Chr^*^ | Start^a^ | End^b^ | -Log10 (p) | Correlation | Samples |
| DTL | 1 | 212208919 | 212278187 | 141.7 | 0.837 | 538 |
| FAM111B | 11 | 58874658 | 58894888 | 122.5 | 0.804 | 538 |
| MCM10 | 10 | 13203554 | 13253104 | 120.2 | 0.8 | 538 |
| CKAP2L | 2 | 113495444 | 113522254 | 113.6 | 0.787 | 538 |
| EXO1 | 1 | 242011493 | 242053241 | 110.5 | 0.78 | 538 |
| FANCI | 15 | 89787194 | 89860362 | 108.8 | 0.776 | 538 |
| CLSPN | 1 | 36197713 | 36235551 | 104.4 | 0.767 | 538 |
| ESCO2 | 8 | 27632058 | 27670141 | 102.1 | 0.761 | 538 |
| TOP2A | 17 | 38544773 | 38574202 | 100.9 | 0.758 | 538 |
| KIF11 | 10 | 94352825 | 94415152 | 100.9 | 0.758 | 538 |
| PLK4 | 4 | 128802016 | 128820377 | 100.8 | 0.758 | 538 |
| E2F7 | 12 | 77415026 | 77459360 | 93.7 | 0.74 | 538 |
| ORC1L | 1 | 52838501 | 52870143 | 93.2 | 0.739 | 538 |
| KIF14 | 1 | 200520625 | 200589862 | 91.9 | 0.735 | 538 |
| MKI67 | 10 | 129894925 | 129924468 | 91.2 | 0.733 | 538 |
| CDC6 | 17 | 38444146 | 38459413 | 90.5 | 0.732 | 538 |
| MELK | 9 | 36572905 | 36677679 | 88.2 | 0.725 | 538 |
| RAD51AP1 | 12 | 4647950 | 4669213 | 88.1 | 0.725 | 538 |
| BUB1B | 15 | 40453210 | 40513337 | 87.7 | 0.724 | 538 |
| DEPDC1 | 1 | 68939835 | 68962799 | 85.8 | 0.718 | 538 |
| PRR11 | 17 | 57232860 | 57284070 | 84.2 | 0.714 | 538 |
| CDC45 | 22 | 19467414 | 19508135 | 82.9 | 0.71 | 538 |
| POLQ | 3 | 121150273 | 121264853 | 82.8 | 0.71 | 538 |
| NCAPG | 4 | 17812525 | 17846487 | 82.3 | 0.708 | 538 |
| KIF18A | 11 | 28042163 | 28129746 | 82.3 | 0.708 | 538 |
| KIF20B | 10 | 91461367 | 91534700 | 81.2 | 0.705 | 538 |
| NUSAP1 | 15 | 41624892 | 41673248 | 80.9 | 0.704 | 538 |
| CENPF | 1 | 214776532 | 214837914 | 80.8 | 0.703 | 538 |
| RRM2 | 2 | 10262695 | 10271546 | 80.4 | 0.702 | 538 |
| XRCC2 | 7 | 152343587 | 152373250 | 80 | 0.701 | 538 |
| NCAPH | 2 | 97001484 | 97041274 | 79.4 | 0.699 | 538 |
| RAD51 | 15 | 40987327 | 41024356 | 79.1 | 0.698 | 538 |
| EZH2 | 7 | 148504464 | 148581441 | 78.7 | 0.697 | 538 |
| NEK2 | 1 | 211831599 | 211848972 | 78.6 | 0.697 | 538 |
| STIL | 1 | 47715811 | 47779819 | 78.1 | 0.695 | 538 |
| UHRF1 | 19 | 4909510 | 4962165 | 77.6 | 0.694 | 538 |
| ERCC6L | X | 71424507 | 71458858 | 77.5 | 0.693 | 538 |
| BUB1 | 2 | 111395409 | 111435684 | 77.1 | 0.692 | 538 |
| CENPK | 5 | 64813593 | 64858995 | 76.8 | 0.691 | 538 |
| DLGAP5 | 14 | 55614834 | 55658396 | 75.5 | 0.686 | 538 |
| E2F2 | 1 | 23832920 | 23857712 | 75.3 | 0.686 | 538 |
| KIF15 | 3 | 44803209 | 44894748 | 74.7 | 0.684 | 538 |
| WDR76 | 15 | 44119112 | 44160617 | 74 | 0.681 | 538 |
| GSG2 | 17 | 3627197 | 3629992 | 73.7 | 0.681 | 538 |
| PBK | 8 | 27667138 | 27695572 | 73.5 | 0.68 | 538 |
| SKA3 | 13 | 21727734 | 21750741 | 73.3 | 0.679 | 538 |
| CCNE2 | 8 | 95892453 | 95907482 | 72.9 | 0.678 | 538 |
| KIFC1 | 6 | 33359313 | 33377699 | 72.2 | 0.675 | 538 |
| HMMR | 5 | 162887517 | 162918953 | 72 | 0.675 | 538 |
| KIF4A | X | 69509879 | 69640774 | 71.1 | 0.671 | 538 |
| LMNB1 | 5 | 126112315 | 126172712 | 70.5 | 0.67 | 538 |
| CENPE | 4 | 104026963 | 104119566 | 70.3 | 0.669 | 538 |
| PRC1 | 15 | 91509268 | 91537804 | 70.2 | 0.669 | 538 |
| SGOL2 | 2 | 201390865 | 201448818 | 69.9 | 0.667 | 538 |
| CEP55 | 10 | 95256369 | 95288849 | 69.8 | 0.667 | 538 |
| TTK | 6 | 80714322 | 80752244 | 69.4 | 0.665 | 538 |
| CDCA5 | 11 | 64844927 | 64851615 | 69.2 | 0.665 | 538 |
| NUF2 | 1 | 163291723 | 163325553 | 67.2 | 0.657 | 538 |
| TPX2 | 20 | 30326904 | 30389603 | 66.7 | 0.656 | 538 |
| ZWINT | 10 | 58117199 | 58121034 | 65.4 | 0.651 | 538 |
| KIAA0101 | 15 | 64657211 | 64673702 | 65.4 | 0.651 | 538 |
| NCAPG2 | 7 | 158424003 | 158497520 | 65.1 | 0.65 | 538 |
| FOXM1 | 12 | 2966847 | 2986321 | 64.5 | 0.647 | 538 |
| CCNA2 | 4 | 122737599 | 122745088 | 64.5 | 0.648 | 538 |
| E2F8 | 11 | 19245610 | 19263167 | 64.3 | 0.647 | 538 |
| NEIL3 | 4 | 178230991 | 178284092 | 62.9 | 0.641 | 538 |
| GINS1 | 20 | 25388323 | 25429191 | 62 | 0.638 | 538 |
| IQGAP3 | 1 | 156495197 | 156542396 | 61 | 0.634 | 538 |
| CDCA7 | 2 | 174219561 | 174233718 | 60.8 | 0.633 | 538 |
| HJURP | 2 | 234745486 | 234763212 | 60.2 | 0.631 | 538 |
| OIP5 | 15 | 41601466 | 41624819 | 60.1 | 0.63 | 538 |
| TMEM194A | 12 | 57449426 | 57472574 | 59.5 | 0.627 | 538 |
| CDCA8 | 1 | 38158159 | 38175391 | 59.5 | 0.628 | 538 |
| CDC25C | 5 | 137620959 | 137674044 | 57.8 | 0.62 | 538 |
| GTSE1 | 22 | 46692638 | 46726707 | 57.5 | 0.619 | 538 |
| RACGAP1 | 12 | 50382945 | 50419307 | 57.4 | 0.619 | 538 |
| KIF18B | 17 | 43002082 | 43025082 | 57.3 | 0.618 | 538 |
| CCDC88A | 2 | 55514978 | 55647057 | 57.2 | 0.618 | 538 |
| C11ORF82 | 11 | 82612737 | 82669319 | 56.7 | 0.616 | 538 |
| C1ORF112 | 1 | 169631245 | 169822229 | 56.5 | 0.615 | 538 |
| CKAP2 | 13 | 53029495 | 53050763 | 56.1 | 0.613 | 538 |
| WDHD1 | 14 | 55405656 | 55493819 | 56 | 0.613 | 538 |
| TYMS | 18 | 657604 | 673499 | 55.8 | 0.612 | 538 |
| SKA1 | 18 | 47901392 | 47920538 | 55.4 | 0.61 | 538 |
| CCNB2 | 15 | 59397284 | 59417244 | 54.8 | 0.607 | 538 |
| MAD2L1 | 4 | 120980579 | 120988013 | 54.4 | 0.605 | 538 |
| EME1 | 17 | 48450581 | 48458820 | 53.9 | 0.603 | 538 |
| FAM72D | 1 | 143896452 | 143913143 | 53.6 | 0.602 | 538 |
| C6ORF167 | 6 | 97590037 | 97731061 | 53.6 | 0.602 | 538 |
| TOPBP1 | 3 | 133319449 | 133380737 | 52.9 | 0.599 | 538 |
| FANCA | 16 | 89803959 | 89883065 | 52.5 | 0.597 | 538 |
| PKMYT1 | 16 | 3022792 | 3030540 | 52.3 | 0.596 | 538 |
| GINS4 | 8 | 41386725 | 41402565 | 52.3 | 0.596 | 538 |
| MLF1IP | 4 | 185615219 | 185655286 | 52 | 0.594 | 538 |
| KIF2C | 1 | 45205490 | 45233438 | 51.9 | 0.594 | 538 |
| PLK1 | 16 | 23690201 | 23701688 | 51.5 | 0.592 | 538 |
| CDK1 | 10 | 62538089 | 62554610 | 51.4 | 0.592 | 538 |
| CENPA | 2 | 27008882 | 27023934 | 51.3 | 0.591 | 538 |
| STK4 | 20 | 43595120 | 43708618 | 51.1 | 0.59 | 538 |
| KIF20A | 5 | 137514417 | 137523404 | 51 | 0.59 | 538 |
| C15ORF42 | 15 | 90118818 | 90171253 | 51 | 0.59 | 538 |
| BRCA2 | 13 | 32889617 | 32973809 | 115.5 | 0.791 | 538 |
| BRCA1 | 17 | 41196312 | 41322420 | 123.4 | 0.806 | 538 |
| BLM | 15 | 91260579 | 91358686 | 67.1 | 0.657 | 538 |
| ATAD5 | 17 | 29159023 | 29222295 | 76.9 | 0.691 | 538 |
| ATAD2 | 8 | 124332091 | 124428590 | 89.6 | 0.729 | 538 |
| ASPM | 1 | 197053257 | 197115824 | 102.7 | 0.762 | 538 |
| ASF1B | 19 | 14230321 | 14247440 | 65.1 | 0.65 | 538 |
| ARHGAP11A | 15 | 32907345 | 32931868 | 106.9 | 0.772 | 538 |
| ANLN | 7 | 36429432 | 36493400 | 86.4 | 0.72 | 538 |
| ANKRD32 | 5 | 93954391 | 94031573 | 51.2 | 0.591 | 538 |
| KIRC: Kidney renal clear cell carcinoma, *: Chromosome, a: chromosome starting site of the gene, b: chromosome ending site of the gene. | | | | | | |

| Table S16. The correlation between BRIP1 and other genes of LGG from the TCGA database | | | | | | |
| --- | --- | --- | --- | --- | --- | --- |
| Genes | Chr^*^ | Start^a^ | End^b^ | -Log10 (p) | Correlation | Samples |
| CKAP2L | 2 | 113495444 | 113522254 | 118 | 0.93 | 271 |
| EXO1 | 1 | 242011493 | 242053241 | 113.6 | 0.924 | 271 |
| ESCO2 | 8 | 27632058 | 27670141 | 112.1 | 0.922 | 271 |
| DTL | 1 | 212208919 | 212278187 | 111.2 | 0.921 | 271 |
| NCAPH | 2 | 97001484 | 97041274 | 107.6 | 0.915 | 271 |
| SGOL1 | 3 | 20202085 | 20227724 | 106.8 | 0.914 | 271 |
| TOP2A | 17 | 38544773 | 38574202 | 106.4 | 0.914 | 271 |
| CASC5 | 15 | 40886447 | 40954881 | 103.4 | 0.909 | 271 |
| TPX2 | 20 | 30326904 | 30389603 | 102.1 | 0.907 | 271 |
| CENPF | 1 | 214776532 | 214837914 | 102 | 0.906 | 271 |
| RRM2 | 2 | 10262695 | 10271546 | 101.9 | 0.906 | 271 |
| BUB1 | 2 | 111395409 | 111435684 | 99.5 | 0.902 | 271 |
| FAM111B | 11 | 58874658 | 58894888 | 99.4 | 0.902 | 271 |
| FANCI | 15 | 89787194 | 89860362 | 99.1 | 0.902 | 271 |
| POLQ | 3 | 121150273 | 121264853 | 97.2 | 0.898 | 271 |
| ESPL1 | 12 | 53662083 | 53687427 | 96.8 | 0.897 | 271 |
| MELK | 9 | 36572905 | 36677679 | 95.8 | 0.896 | 271 |
| CDC45 | 22 | 19467414 | 19508135 | 95 | 0.894 | 271 |
| KIFC1 | 6 | 33359313 | 33377699 | 94.4 | 0.893 | 271 |
| PBK | 8 | 27667138 | 27695572 | 94.3 | 0.893 | 271 |
| CDK1 | 10 | 62538089 | 62554610 | 94.2 | 0.892 | 271 |
| TTK | 6 | 80714322 | 80752244 | 94 | 0.892 | 271 |
| NCAPG | 4 | 17812525 | 17846487 | 93.9 | 0.892 | 271 |
| SKA3 | 13 | 21727734 | 21750741 | 93.8 | 0.892 | 271 |
| MKI67 | 10 | 129894925 | 129924468 | 93.7 | 0.891 | 271 |
| BUB1B | 15 | 40453210 | 40513337 | 93.6 | 0.891 | 271 |
| KIF14 | 1 | 200520625 | 200589862 | 93.3 | 0.891 | 271 |
| NUSAP1 | 15 | 41624892 | 41673248 | 93.1 | 0.89 | 271 |
| PRC1 | 15 | 91509268 | 91537804 | 92.7 | 0.889 | 271 |
| KIF23 | 15 | 69706627 | 69740764 | 92.6 | 0.889 | 271 |
| HJURP | 2 | 234745486 | 234763212 | 90.7 | 0.885 | 271 |
| ERCC6L | X | 71424507 | 71458858 | 90.4 | 0.885 | 271 |
| GSG2 | 17 | 3627197 | 3629992 | 90.3 | 0.884 | 271 |
| PLK4 | 4 | 128802016 | 128820377 | 90 | 0.884 | 271 |
| KIF20A | 5 | 137514417 | 137523404 | 89.1 | 0.882 | 271 |
| MCM10 | 10 | 13203554 | 13253104 | 88.4 | 0.88 | 271 |
| SKA1 | 18 | 47901392 | 47920538 | 87.6 | 0.879 | 271 |
| FOXM1 | 12 | 2966847 | 2986321 | 87.4 | 0.878 | 271 |
| CDCA5 | 11 | 64844927 | 64851615 | 86.9 | 0.877 | 271 |
| KIF11 | 10 | 94352825 | 94415152 | 86.8 | 0.877 | 271 |
| NDC80 | 18 | 2571510 | 2616634 | 86.7 | 0.877 | 271 |
| IQGAP3 | 1 | 156495197 | 156542396 | 86.7 | 0.877 | 271 |
| GTSE1 | 22 | 46692638 | 46726707 | 86.2 | 0.876 | 271 |
| CENPK | 5 | 64813593 | 64858995 | 85 | 0.873 | 271 |
| CLSPN | 1 | 36197713 | 36235551 | 84.7 | 0.872 | 271 |
| RAD51 | 15 | 40987327 | 41024356 | 84.6 | 0.872 | 271 |
| FAM64A | 17 | 6347735 | 6354385 | 83.4 | 0.869 | 271 |
| CCNB2 | 15 | 59397284 | 59417244 | 82.8 | 0.868 | 271 |
| DLGAP5 | 14 | 55614834 | 55658396 | 82.7 | 0.867 | 271 |
| MYBL2 | 20 | 42295709 | 42345122 | 82.6 | 0.867 | 271 |
| SPC24 | 19 | 11257831 | 11266484 | 82.2 | 0.866 | 271 |
| NUF2 | 1 | 163291723 | 163325553 | 82.1 | 0.866 | 271 |
| KIAA0101 | 15 | 64657211 | 64673702 | 81.6 | 0.865 | 271 |
| FANCD2 | 3 | 10068113 | 10143614 | 81.3 | 0.864 | 271 |
| E2F7 | 12 | 77415026 | 77459360 | 80.5 | 0.862 | 271 |
| KIF4A | X | 69509879 | 69640774 | 80.1 | 0.861 | 271 |
| CDC25C | 5 | 137620959 | 137674044 | 79.1 | 0.858 | 271 |
| KIF15 | 3 | 44803209 | 44894748 | 78.9 | 0.858 | 271 |
| ZWINT | 10 | 58117199 | 58121034 | 78.6 | 0.857 | 271 |
| HMMR | 5 | 162887517 | 162918953 | 78.6 | 0.857 | 271 |
| CENPE | 4 | 104026963 | 104119566 | 77.2 | 0.853 | 271 |
| NEK2 | 1 | 211831599 | 211848972 | 76.8 | 0.852 | 271 |
| GINS1 | 20 | 25388323 | 25429191 | 76.5 | 0.851 | 271 |
| CDCA2 | 8 | 25316513 | 25365425 | 76.4 | 0.851 | 271 |
| TRIP13 | 5 | 892969 | 918164 | 76.3 | 0.851 | 271 |
| ZNF367 | 9 | 99148225 | 99180669 | 76.1 | 0.85 | 271 |
| NEIL3 | 4 | 178230991 | 178284092 | 76.1 | 0.85 | 271 |
| CCNA2 | 4 | 122737599 | 122745088 | 76.1 | 0.85 | 271 |
| ECT2 | 3 | 172468532 | 172539263 | 73.4 | 0.842 | 271 |
| CDC6 | 17 | 38444146 | 38459413 | 71.9 | 0.838 | 271 |
| CENPA | 2 | 27008882 | 27023934 | 71.8 | 0.837 | 271 |
| UBE2C | 20 | 44441255 | 44445596 | 71.5 | 0.837 | 271 |
| MLF1IP | 4 | 185615219 | 185655286 | 70.7 | 0.834 | 271 |
| CEP55 | 10 | 95256369 | 95288849 | 70.5 | 0.833 | 271 |
| FANCA | 16 | 89803959 | 89883065 | 70.4 | 0.833 | 271 |
| TROAP | 12 | 49716971 | 49725514 | 70.3 | 0.833 | 271 |
| C11ORF82 | 11 | 82612737 | 82669319 | 70.2 | 0.832 | 271 |
| TIMELESS | 12 | 56810157 | 56843200 | 70.1 | 0.832 | 271 |
| SHCBP1 | 16 | 46614468 | 46655311 | 70.1 | 0.832 | 271 |
| DEPDC1B | 5 | 59892739 | 59995993 | 69 | 0.828 | 271 |
| POC1A | 3 | 52109249 | 52188706 | 68.5 | 0.827 | 271 |
| HELLS | 10 | 96305543 | 96361856 | 68 | 0.825 | 271 |
| CENPI | X | 100353178 | 100417978 | 67.5 | 0.824 | 271 |
| DEPDC1 | 1 | 68939835 | 68962799 | 67.1 | 0.822 | 271 |
| RAD51AP1 | 12 | 4647950 | 4669213 | 66.6 | 0.821 | 271 |
| TACC3 | 4 | 1723217 | 1746905 | 66.3 | 0.82 | 271 |
| SGOL2 | 2 | 201390865 | 201448818 | 66.2 | 0.819 | 271 |
| TK1 | 17 | 76170160 | 76183285 | 65.8 | 0.818 | 271 |
| XRCC2 | 7 | 152343587 | 152373250 | 64.4 | 0.813 | 271 |
| LMNB1 | 5 | 126112315 | 126172712 | 63.7 | 0.811 | 271 |
| WDR62 | 19 | 36545783 | 36596012 | 62.2 | 0.805 | 271 |
| E2F8 | 11 | 19245610 | 19263167 | 61.9 | 0.804 | 271 |
| PTTG1 | 5 | 159848865 | 159855746 | 61.3 | 0.802 | 271 |
| PKMYT1 | 16 | 3022792 | 3030540 | 61.1 | 0.801 | 271 |
| KIAA1524 | 3 | 108268718 | 108308491 | 60.9 | 0.8 | 271 |
| ORC1 | 1 | 52838501 | 52870143 | 60.5 | 0.798 | 271 |
| OIP5 | 15 | 41601466 | 41624819 | 60.3 | 0.798 | 271 |
| CDCA8 | 1 | 38158159 | 38175391 | 60.3 | 0.798 | 271 |
| SPAG5 | 17 | 26904583 | 26941211 | 60.2 | 0.797 | 271 |
| RAD54L | 1 | 46713367 | 46744145 | 59.2 | 0.794 | 271 |
| ORC6 | 16 | 46723558 | 46732306 | 58.2 | 0.789 | 271 |
| PARPBP | 12 | 102513956 | 102591298 | 58.2 | 0.789 | 271 |
| CCNB1 | 5 | 68462837 | 68474070 | 58 | 0.789 | 271 |
| EME1 | 17 | 48450581 | 48458820 | 57.8 | 0.788 | 271 |
| KNTC1 | 12 | 123011809 | 123110947 | 57 | 0.784 | 271 |
| MCM2 | 3 | 127317253 | 127341278 | 56 | 0.78 | 271 |
| MCM4 | 8 | 48872763 | 48890719 | 55.8 | 0.779 | 271 |
| SMC4 | 3 | 160117430 | 160152741 | 55 | 0.776 | 271 |
| RTKN2 | 10 | 63942794 | 64028466 | 55 | 0.776 | 271 |
| CHAF1A | 19 | 4402660 | 4443394 | 54.6 | 0.774 | 271 |
| CDT1 | 16 | 88870186 | 88875666 | 54.5 | 0.774 | 271 |
| WDHD1 | 14 | 55405656 | 55493819 | 54.4 | 0.773 | 271 |
| CENPM | 22 | 42334741 | 42343148 | 53.7 | 0.77 | 271 |
| TCF19 | 6 | 31126303 | 31131992 | 53.2 | 0.768 | 271 |
| KIF2C | 1 | 45205490 | 45233438 | 53 | 0.767 | 271 |
| PLK1 | 16 | 23690201 | 23701688 | 52.2 | 0.763 | 271 |
| GAS2L3 | 12 | 100967489 | 101018685 | 52.1 | 0.763 | 271 |
| EZH2 | 7 | 148504464 | 148581441 | 52 | 0.762 | 271 |
| RFWD3 | 16 | 74655297 | 74700779 | 51.2 | 0.758 | 271 |
| SPC25 | 2 | 169727401 | 169746944 | 51 | 0.757 | 271 |
| MAD2L1 | 4 | 120980579 | 120988013 | 51 | 0.757 | 271 |
| KIF18A | 11 | 28042163 | 28129746 | 50.6 | 0.755 | 271 |
| CENPL | 1 | 173768688 | 173793777 | 50.1 | 0.753 | 271 |
| C17ORF53 | 17 | 42219274 | 42239844 | 49.7 | 0.751 | 271 |
| TYMS | 18 | 657604 | 673499 | 49.1 | 0.748 | 271 |
| CDKN3 | 14 | 54863673 | 54886934 | 49 | 0.748 | 271 |
| RMI2 | 16 | 11343506 | 11445617 | 48.9 | 0.747 | 271 |
| GINS2 | 16 | 85711280 | 85722588 | 48.3 | 0.744 | 271 |
| C5ORF34 | 5 | 43486803 | 43515273 | 47.5 | 0.74 | 271 |
| CDC25A | 3 | 48198668 | 48229801 | 47.1 | 0.738 | 271 |
| TRAIP | 3 | 49866028 | 49893992 | 46.5 | 0.734 | 271 |
| E2F2 | 1 | 23832920 | 23857712 | 46 | 0.732 | 271 |
| MCM8 | 20 | 5931298 | 5975831 | 46 | 0.732 | 271 |
| FAM72D | 1 | 143896452 | 143913143 | 45.9 | 0.732 | 271 |
| CENPO | 2 | 25016175 | 25045245 | 45.4 | 0.728 | 271 |
| MND1 | 4 | 154265801 | 154336247 | 45.3 | 0.728 | 271 |
| NCAPG2 | 7 | 158424003 | 158497520 | 45 | 0.727 | 271 |
| FANCC | 9 | 97861336 | 98079991 | 45 | 0.726 | 271 |
| CKAP2 | 13 | 53029495 | 53050763 | 45 | 0.726 | 271 |
| E2F1 | 20 | 32263292 | 32274210 | 44.9 | 0.726 | 271 |
| FAM83D | 20 | 37554955 | 37581703 | 44.4 | 0.723 | 271 |
| CHEK1 | 11 | 125495031 | 125546150 | 44.4 | 0.723 | 271 |
| GEN1 | 2 | 17935162 | 17966632 | 44.3 | 0.723 | 271 |
| CDC20 | 1 | 43824626 | 43828873 | 44.1 | 0.721 | 271 |
| POLE2 | 14 | 50110270 | 50155098 | 44 | 0.721 | 271 |
| UBE2T | 1 | 202300785 | 202311094 | 43.8 | 0.72 | 271 |
| ZWILCH | 15 | 66797431 | 66841822 | 43.7 | 0.719 | 271 |
| MCM6 | 2 | 136597196 | 136634011 | 43.6 | 0.718 | 271 |
| DNMT1 | 19 | 10244022 | 10305755 | 43 | 0.715 | 271 |
| RACGAP1 | 12 | 50382945 | 50419307 | 42.8 | 0.714 | 271 |
| FBXO5 | 6 | 153291658 | 153304740 | 42.7 | 0.714 | 271 |
| KIF18B | 17 | 43002082 | 43025082 | 42 | 0.709 | 271 |
| STIL | 1 | 47715811 | 47779819 | 41.7 | 0.707 | 271 |
| CENPH | 5 | 68485375 | 68506184 | 41.1 | 0.704 | 271 |
| TOP3A | 17 | 18177235 | 18218321 | 40.9 | 0.702 | 271 |
| TMPO | 12 | 98909351 | 98944157 | 40.9 | 0.702 | 271 |
| DSN1 | 20 | 35380194 | 35402230 | 40.3 | 0.699 | 271 |
| DBF4 | 7 | 87505544 | 87538856 | 39.7 | 0.695 | 271 |
| WDR76 | 15 | 44119112 | 44160617 | 39.6 | 0.694 | 271 |
| KPNA2 | 17 | 66031848 | 66042970 | 39.6 | 0.695 | 271 |
| C9ORF100 | 9 | 35658872 | 35675863 | 39 | 0.69 | 271 |
| GINS4 | 8 | 41386725 | 41402565 | 38.4 | 0.686 | 271 |
| SMC2 | 9 | 106856541 | 106903700 | 38.2 | 0.686 | 271 |
| FAM54A | 6 | 136552168 | 136571449 | 38.1 | 0.685 | 271 |
| CKS2 | 9 | 91926113 | 91931618 | 37.6 | 0.681 | 271 |
| FAM72B | 1 | 120839005 | 120855681 | 36.4 | 0.673 | 271 |
| SDC1 | 2 | 20400558 | 20425194 | 36.2 | 0.672 | 271 |
| FEN1 | 11 | 61560109 | 61564714 | 36 | 0.67 | 271 |
| LMNB2 | 19 | 2428164 | 2456958 | 35.9 | 0.669 | 271 |
| RBL1 | 20 | 35626178 | 35724410 | 35.7 | 0.668 | 271 |
| CCDC15 | 11 | 124824017 | 124911385 | 35.6 | 0.667 | 271 |
| KIF20B | 10 | 91461367 | 91534700 | 35.6 | 0.668 | 271 |
| CENPN | 16 | 81040103 | 81066709 | 35.5 | 0.667 | 271 |
| RFC4 | 3 | 186507682 | 186524484 | 35.2 | 0.665 | 271 |
| TONSL | 8 | 145654163 | 145669812 | 34.9 | 0.663 | 271 |
| CEP152 | 15 | 49030135 | 49103343 | 34.4 | 0.659 | 271 |
| WDR67 | 8 | 124084920 | 124164392 | 34.3 | 0.658 | 271 |
| RECQL4 | 8 | 145736667 | 145743210 | 33.8 | 0.654 | 271 |
| CDCA4 | 14 | 105475910 | 105487425 | 33.4 | 0.652 | 271 |
| CENPW | 6 | 126661253 | 126669754 | 33.3 | 0.651 | 271 |
| TRIM24 | 7 | 138145079 | 138270332 | 33.3 | 0.651 | 271 |
| CHEK2 | 22 | 29083731 | 29137822 | 33 | 0.648 | 271 |
| POLE | 12 | 133200348 | 133263945 | 32.8 | 0.647 | 271 |
| PRR11 | 17 | 57232860 | 57284070 | 31.9 | 0.64 | 271 |
| CDCA3 | 12 | 6953963 | 6960456 | 31.9 | 0.64 | 271 |
| RFC3 | 13 | 34392206 | 34540695 | 31.8 | 0.639 | 271 |
| POLA2 | 11 | 65029432 | 65065088 | 31.7 | 0.638 | 271 |
| HAUS8 | 19 | 17160571 | 17186343 | 31.5 | 0.637 | 271 |
| TMEM194A | 12 | 57449426 | 57472574 | 31.4 | 0.636 | 271 |
| BRCA2 | 13 | 32889617 | 32973809 | 78.5 | 0.857 | 271 |
| BIRC5 | 17 | 76210277 | 76221716 | 80.8 | 0.863 | 271 |
| AURKB | 17 | 8108049 | 8113883 | 76.8 | 0.852 | 271 |
| AURKA | 20 | 54944445 | 54967351 | 68.3 | 0.826 | 271 |
| ATAD5 | 17 | 29159023 | 29222295 | 48.9 | 0.747 | 271 |
| ATAD2 | 8 | 124332091 | 124428590 | 46.7 | 0.736 | 271 |
| ASPM | 1 | 197053257 | 197115824 | 96.4 | 0.897 | 271 |
| ASF1B | 19 | 14230321 | 14247440 | 84.3 | 0.871 | 271 |
| ARHGAP11B | 15 | 30916697 | 30977810 | 81 | 0.863 | 271 |
| ARHGAP11A | 15 | 32907345 | 32931868 | 93.6 | 0.891 | 271 |
| ANXA7 | 10 | 75135189 | 75173841 | 31.4 | -0.636 | 271 |
| LGG: Brain Lower Grade Glioma, *: Chromosome, a: chromosome starting site of the gene, b: chromosome ending site of the gene. | | | | | | |

| Table S17. The correlation between BRIP1 and other genes of LUAD from the TCGA database | | | | | | |
| --- | --- | --- | --- | --- | --- | --- |
| Genes | Chr^*^ | Start^a^ | End^b^ | -Log10 (p) | Correlation | Samples |
| CLSPN | 1 | 36197713 | 36235551 | 98.9 | 0.849 | 353 |
| PRR11 | 17 | 57232860 | 57284070 | 96.1 | 0.843 | 353 |
| DTL | 1 | 212208919 | 212278187 | 82.8 | 0.81 | 353 |
| TOP2A | 17 | 38544773 | 38574202 | 78.2 | 0.796 | 353 |
| KIF18B | 17 | 43002082 | 43025082 | 78.2 | 0.796 | 353 |
| CKAP2L | 2 | 113495444 | 113522254 | 78 | 0.796 | 353 |
| KIF15 | 3 | 44803209 | 44894748 | 77.3 | 0.794 | 353 |
| WDHD1 | 14 | 55405656 | 55493819 | 76.7 | 0.792 | 353 |
| STIL | 1 | 47715811 | 47779819 | 76.7 | 0.792 | 353 |
| CASC5 | 15 | 40886447 | 40954881 | 75.9 | 0.789 | 353 |
| CENPI | X | 100353178 | 100417978 | 74.9 | 0.786 | 353 |
| RAD54L | 1 | 46713367 | 46744145 | 74.1 | 0.783 | 353 |
| KIF4A | X | 69509879 | 69640774 | 74 | 0.783 | 353 |
| ORC1 | 1 | 52838501 | 52870143 | 73.1 | 0.78 | 353 |
| RBL1 | 20 | 35626178 | 35724410 | 72.9 | 0.78 | 353 |
| MCM2 | 3 | 127317253 | 127341278 | 72.5 | 0.778 | 353 |
| CDC6 | 17 | 38444146 | 38459413 | 72.4 | 0.778 | 353 |
| PLK4 | 4 | 128802016 | 128820377 | 72.3 | 0.778 | 353 |
| CENPE | 4 | 104026963 | 104119566 | 72.2 | 0.777 | 353 |
| MCM6 | 2 | 136597196 | 136634011 | 72.2 | 0.777 | 353 |
| XRCC2 | 7 | 152343587 | 152373250 | 71.9 | 0.776 | 353 |
| POLQ | 3 | 121150273 | 121264853 | 71.8 | 0.776 | 353 |
| TPX2 | 20 | 30326904 | 30389603 | 70.6 | 0.772 | 353 |
| BUB1B | 15 | 40453210 | 40513337 | 70.6 | 0.772 | 353 |
| KIF23 | 15 | 69706627 | 69740764 | 70.4 | 0.771 | 353 |
| NCAPG2 | 7 | 158424003 | 158497520 | 68.8 | 0.765 | 353 |
| ESPL1 | 12 | 53662083 | 53687427 | 68.7 | 0.765 | 353 |
| CHAF1A | 19 | 4402660 | 4443394 | 68.5 | 0.765 | 353 |
| KIAA1524 | 3 | 108268718 | 108308491 | 68.1 | 0.763 | 353 |
| EXO1 | 1 | 242011493 | 242053241 | 68.1 | 0.763 | 353 |
| BUB1 | 2 | 111395409 | 111435684 | 67.6 | 0.761 | 353 |
| KIF14 | 1 | 200520625 | 200589862 | 67.4 | 0.76 | 353 |
| SGOL1 | 3 | 20202085 | 20227724 | 67 | 0.759 | 353 |
| KIF11 | 10 | 94352825 | 94415152 | 67 | 0.759 | 353 |
| MCM4 | 8 | 48872763 | 48890719 | 66.8 | 0.758 | 353 |
| SPAG5 | 17 | 26904583 | 26941211 | 66.7 | 0.758 | 353 |
| MCM10 | 10 | 13203554 | 13253104 | 66.6 | 0.758 | 353 |
| C15ORF42 | 15 | 90118818 | 90171253 | 66.3 | 0.757 | 353 |
| NCAPH | 2 | 97001484 | 97041274 | 66.2 | 0.756 | 353 |
| HELLS | 10 | 96305543 | 96361856 | 66.1 | 0.756 | 353 |
| UHRF1 | 19 | 4909510 | 4962165 | 65.9 | 0.755 | 353 |
| FAM111B | 11 | 58874658 | 58894888 | 65.6 | 0.754 | 353 |
| GTSE1 | 22 | 46692638 | 46726707 | 65 | 0.752 | 353 |
| ZNF367 | 9 | 99148225 | 99180669 | 64.5 | 0.75 | 353 |
| MKI67 | 10 | 129894925 | 129924468 | 64.5 | 0.75 | 353 |
| KPNA2 | 17 | 66031848 | 66042970 | 64.4 | 0.749 | 353 |
| FANCI | 15 | 89787194 | 89860362 | 64.3 | 0.749 | 353 |
| TOPBP1 | 3 | 133319449 | 133380737 | 64.2 | 0.749 | 353 |
| DIAPH3 | 13 | 60239723 | 60738119 | 63.5 | 0.746 | 353 |
| KNTC1 | 12 | 123011809 | 123110947 | 62.5 | 0.742 | 353 |
| SPC24 | 19 | 11257831 | 11266484 | 62.3 | 0.741 | 353 |
| KIFC1 | 6 | 33359313 | 33377699 | 61.5 | 0.738 | 353 |
| NCAPG | 4 | 17812525 | 17846487 | 61.3 | 0.737 | 353 |
| DEPDC1 | 1 | 68939835 | 68962799 | 61.3 | 0.737 | 353 |
| WDR76 | 15 | 44119112 | 44160617 | 61.2 | 0.737 | 353 |
| TYMS | 18 | 657604 | 673499 | 60.8 | 0.735 | 353 |
| LMNB1 | 5 | 126112315 | 126172712 | 60.6 | 0.735 | 353 |
| CDCA5 | 11 | 64844927 | 64851615 | 60.5 | 0.734 | 353 |
| KIF20B | 10 | 91461367 | 91534700 | 60 | 0.732 | 353 |
| NUSAP1 | 15 | 41624892 | 41673248 | 59.9 | 0.731 | 353 |
| MELK | 9 | 36572905 | 36677679 | 59.9 | 0.731 | 353 |
| C11ORF82 | 11 | 82612737 | 82669319 | 59.9 | 0.731 | 353 |
| DLGAP5 | 14 | 55614834 | 55658396 | 59.8 | 0.731 | 353 |
| KIF2C | 1 | 45205490 | 45233438 | 59.7 | 0.73 | 353 |
| CHEK1 | 11 | 125495031 | 125546150 | 59.3 | 0.729 | 353 |
| TTK | 6 | 80714322 | 80752244 | 59.1 | 0.728 | 353 |
| IQGAP3 | 1 | 156495197 | 156542396 | 59 | 0.728 | 353 |
| CDCA8 | 1 | 38158159 | 38175391 | 59 | 0.728 | 353 |
| PRC1 | 15 | 91509268 | 91537804 | 58.8 | 0.727 | 353 |
| INCENP | 11 | 61891445 | 61920635 | 58.7 | 0.726 | 353 |
| CDCA2 | 8 | 25316513 | 25365425 | 58.6 | 0.726 | 353 |
| RRM2 | 2 | 10262695 | 10271546 | 58.5 | 0.725 | 353 |
| EME1 | 17 | 48450581 | 48458820 | 58 | 0.723 | 353 |
| SMC2 | 9 | 106856541 | 106903700 | 57.6 | 0.722 | 353 |
| FOXM1 | 12 | 2966847 | 2986321 | 57.5 | 0.721 | 353 |
| CENPO | 2 | 25016175 | 25045245 | 57.4 | 0.721 | 353 |
| CENPF | 1 | 214776532 | 214837914 | 56.6 | 0.717 | 353 |
| SGOL2 | 2 | 201390865 | 201448818 | 56.5 | 0.717 | 353 |
| TCF19 | 6 | 31126303 | 31131992 | 55.7 | 0.713 | 353 |
| NDC80 | 18 | 2571510 | 2616634 | 55.5 | 0.712 | 353 |
| SHCBP1 | 16 | 46614468 | 46655311 | 54.9 | 0.71 | 353 |
| RACGAP1 | 12 | 50382945 | 50419307 | 54.8 | 0.709 | 353 |
| NUP205 | 7 | 135242662 | 135333499 | 54.6 | 0.708 | 353 |
| HJURP | 2 | 234745486 | 234763212 | 54.4 | 0.707 | 353 |
| FANCB | X | 14861529 | 14891184 | 54.3 | 0.707 | 353 |
| SKA1 | 18 | 47901392 | 47920538 | 54.3 | 0.707 | 353 |
| FEN1 | 11 | 61560109 | 61564714 | 54.2 | 0.707 | 353 |
| GSG2 | 17 | 3627197 | 3629992 | 54 | 0.706 | 353 |
| E2F8 | 11 | 19245610 | 19263167 | 54 | 0.705 | 353 |
| FANCD2 | 3 | 10068113 | 10143614 | 53.6 | 0.703 | 353 |
| CDC45 | 22 | 19467414 | 19508135 | 53.1 | 0.701 | 353 |
| PLK1 | 16 | 23690201 | 23701688 | 53.1 | 0.701 | 353 |
| GINS4 | 8 | 41386725 | 41402565 | 53.1 | 0.701 | 353 |
| E2F7 | 12 | 77415026 | 77459360 | 53.1 | 0.701 | 353 |
| KIF18A | 11 | 28042163 | 28129746 | 52.9 | 0.7 | 353 |
| CDK2 | 12 | 56360556 | 56366568 | 52.9 | 0.7 | 353 |
| MMS22L | 6 | 97590037 | 97731061 | 52.8 | 0.7 | 353 |
| ZWINT | 10 | 58117199 | 58121034 | 52.6 | 0.699 | 353 |
| CCNA2 | 4 | 122737599 | 122745088 | 52.2 | 0.697 | 353 |
| CDC7 | 1 | 91966404 | 91991321 | 51.9 | 0.695 | 353 |
| KIF20A | 5 | 137514417 | 137523404 | 51.8 | 0.695 | 353 |
| MSH6 | 2 | 48010221 | 48034092 | 51.7 | 0.694 | 353 |
| FAM72B | 1 | 120839005 | 120855681 | 51.7 | 0.694 | 353 |
| ERCC6L | X | 71424507 | 71458858 | 51.6 | 0.694 | 353 |
| CEP55 | 10 | 95256369 | 95288849 | 51.2 | 0.692 | 353 |
| TIMELESS | 12 | 56810157 | 56843200 | 51.1 | 0.691 | 353 |
| CCNB2 | 15 | 59397284 | 59417244 | 51.1 | 0.691 | 353 |
| LMNB2 | 19 | 2428164 | 2456958 | 50.9 | 0.691 | 353 |
| FAM72D | 1 | 143896452 | 143913143 | 50.9 | 0.691 | 353 |
| TMEM194A | 12 | 57449426 | 57472574 | 50.9 | 0.691 | 353 |
| CDC25A | 3 | 48198668 | 48229801 | 50.8 | 0.69 | 353 |
| WDR62 | 19 | 36545783 | 36596012 | 50.6 | 0.689 | 353 |
| MTBP | 8 | 121457666 | 121535875 | 50.6 | 0.689 | 353 |
| C17ORF53 | 17 | 42219274 | 42239844 | 50.6 | 0.689 | 353 |
| CDC25C | 5 | 137620959 | 137674044 | 50.3 | 0.687 | 353 |
| TMEM48 | 1 | 54231134 | 54304225 | 50.2 | 0.687 | 353 |
| DEPDC1B | 5 | 59892739 | 59995993 | 49.7 | 0.685 | 353 |
| KPNB1 | 17 | 45727275 | 45761004 | 49.4 | 0.683 | 353 |
| CENPA | 2 | 27008882 | 27023934 | 49.4 | 0.683 | 353 |
| SASS6 | 1 | 100549102 | 100598511 | 49.3 | 0.683 | 353 |
| CCNF | 16 | 2479395 | 2508859 | 48.8 | 0.68 | 353 |
| MYBL2 | 20 | 42295709 | 42345122 | 48.4 | 0.678 | 353 |
| NEK2 | 1 | 211831599 | 211848972 | 48.1 | 0.676 | 353 |
| SKA3 | 13 | 21727734 | 21750741 | 47.9 | 0.675 | 353 |
| FAM72A | 1 | 206138911 | 206155074 | 47.7 | 0.674 | 353 |
| E2F2 | 1 | 23832920 | 23857712 | 47.6 | 0.674 | 353 |
| CCNE1 | 19 | 30302901 | 30315215 | 47.6 | 0.674 | 353 |
| RAD51AP1 | 12 | 4647950 | 4669213 | 47.4 | 0.672 | 353 |
| DNMT1 | 19 | 10244022 | 10305755 | 47.4 | 0.673 | 353 |
| CHAF1B | 21 | 37757689 | 37789125 | 47.3 | 0.672 | 353 |
| NUP155 | 5 | 37291941 | 37371197 | 47.2 | 0.672 | 353 |
| PKMYT1 | 16 | 3022792 | 3030540 | 47.1 | 0.671 | 353 |
| ECT2 | 3 | 172468532 | 172539263 | 47.1 | 0.671 | 353 |
| CDC20 | 1 | 43824626 | 43828873 | 46.9 | 0.67 | 353 |
| PSMC3IP | 17 | 40724329 | 40729747 | 46.8 | 0.669 | 353 |
| DBF4B | 17 | 42785976 | 42829636 | 46.7 | 0.669 | 353 |
| MYO19 | 17 | 34851599 | 34891305 | 46.6 | 0.669 | 353 |
| EZH2 | 7 | 148504464 | 148581441 | 46.2 | 0.666 | 353 |
| RAD51 | 15 | 40987327 | 41024356 | 45.9 | 0.665 | 353 |
| TRIP13 | 5 | 892969 | 918164 | 45.6 | 0.663 | 353 |
| MCM8 | 20 | 5931298 | 5975831 | 45.2 | 0.66 | 353 |
| DONSON | 21 | 34947783 | 35288158 | 45.1 | 0.66 | 353 |
| SPC25 | 2 | 169727401 | 169746944 | 44.3 | 0.655 | 353 |
| MAD2L1 | 4 | 120980579 | 120988013 | 44.3 | 0.656 | 353 |
| CENPK | 5 | 64813593 | 64858995 | 44.3 | 0.655 | 353 |
| FBXO5 | 6 | 153291658 | 153304740 | 44.2 | 0.655 | 353 |
| FBXO45 | 3 | 196295725 | 196315930 | 44.2 | 0.655 | 353 |
| ESCO2 | 8 | 27632058 | 27670141 | 44.1 | 0.654 | 353 |
| TACC3 | 4 | 1723217 | 1746905 | 44 | 0.654 | 353 |
| TROAP | 12 | 49716971 | 49725514 | 43.8 | 0.653 | 353 |
| NCAPD3 | 11 | 134022337 | 134094426 | 43.7 | 0.652 | 353 |
| C1ORF112 | 1 | 169631245 | 169822229 | 43.5 | 0.651 | 353 |
| MSH2 | 2 | 47630206 | 47906510 | 43.4 | 0.65 | 353 |
| CST3 | 20 | 23608534 | 23618574 | 43.4 | -0.65 | 353 |
| GEN1 | 2 | 17935162 | 17966632 | 43.2 | 0.649 | 353 |
| RFC4 | 3 | 186507682 | 186524484 | 43.1 | 0.649 | 353 |
| CDC27 | 17 | 45195311 | 45266665 | 43 | 0.648 | 353 |
| E2F1 | 20 | 32263292 | 32274210 | 42.8 | 0.647 | 353 |
| CDK1 | 10 | 62538089 | 62554610 | 42.6 | 0.646 | 353 |
| NUF2 | 1 | 163291723 | 163325553 | 42.5 | 0.645 | 353 |
| C1ORF135 | 1 | 26160497 | 26185848 | 42.5 | 0.645 | 353 |
| CCDC18 | 1 | 93646281 | 93744287 | 42.3 | 0.644 | 353 |
| HMMR | 5 | 162887517 | 162918953 | 42.1 | 0.643 | 353 |
| GMPS | 3 | 155588325 | 155655520 | 42.1 | 0.642 | 353 |
| FAM64A | 17 | 6347735 | 6354385 | 42 | 0.642 | 353 |
| RRM1 | 11 | 4115924 | 4223759 | 41.9 | 0.642 | 353 |
| GINS1 | 20 | 25388323 | 25429191 | 41.9 | 0.642 | 353 |
| CCNB1 | 5 | 68462837 | 68474070 | 41.9 | 0.641 | 353 |
| ORC6 | 16 | 46723558 | 46732306 | 41.8 | 0.641 | 353 |
| DSCC1 | 8 | 120846181 | 120868170 | 41.8 | 0.641 | 353 |
| TRAIP | 3 | 49866028 | 49893992 | 41.7 | 0.64 | 353 |
| GINS3 | 16 | 58426298 | 58440048 | 41.7 | 0.641 | 353 |
| NOL11 | 17 | 65714061 | 65740266 | 41.6 | 0.64 | 353 |
| MASTL | 10 | 27443753 | 27475848 | 41.5 | 0.639 | 353 |
| SUZ12 | 17 | 30264044 | 30328057 | 41.3 | 0.638 | 353 |
| MCM7 | 7 | 99690404 | 99699427 | 41.3 | 0.638 | 353 |
| FAM54A | 6 | 136552168 | 136571449 | 41.2 | 0.638 | 353 |
| EFTUD2 | 17 | 42927655 | 42976993 | 40.9 | 0.636 | 353 |
| MCM3 | 6 | 52128812 | 52149582 | 40.7 | 0.634 | 353 |
| NCAPD2 | 12 | 6603298 | 6641132 | 40.5 | 0.633 | 353 |
| FIGNL1 | 7 | 50511832 | 50518088 | 40.3 | 0.632 | 353 |
| SKP2 | 5 | 36152145 | 36184142 | 40.3 | 0.632 | 353 |
| KIF24 | 9 | 34211974 | 34329198 | 40.3 | 0.632 | 353 |
| FAM83D | 20 | 37554955 | 37581703 | 40.2 | 0.631 | 353 |
| POLE | 12 | 133200348 | 133263945 | 40.1 | 0.631 | 353 |
| PRIM1 | 12 | 57125364 | 57146146 | 40 | 0.63 | 353 |
| BRCA2 | 13 | 32889617 | 32973809 | 60.9 | 0.736 | 353 |
| BRCA1 | 17 | 41196312 | 41322420 | 90.8 | 0.83 | 353 |
| BLM | 15 | 91260579 | 91358686 | 56.8 | 0.718 | 353 |
| BIRC5 | 17 | 76210277 | 76221716 | 44.1 | 0.654 | 353 |
| BARD1 | 2 | 215593275 | 215674428 | 49.1 | 0.682 | 353 |
| AURKB | 17 | 8108049 | 8113883 | 39.7 | 0.628 | 353 |
| AURKA | 20 | 54944445 | 54967351 | 48.6 | 0.679 | 353 |
| ATAD5 | 17 | 29159023 | 29222295 | 70.5 | 0.772 | 353 |
| ATAD2 | 8 | 124332091 | 124428590 | 60.1 | 0.732 | 353 |
| ASPM | 1 | 197053257 | 197115824 | 61.8 | 0.739 | 353 |
| ASF1B | 19 | 14230321 | 14247440 | 50.4 | 0.688 | 353 |
| ARHGAP11B | 15 | 30916697 | 30977810 | 64.5 | 0.75 | 353 |
| ARHGAP11A | 15 | 32907345 | 32931868 | 62.8 | 0.743 | 353 |
| ANLN | 7 | 36429432 | 36493400 | 63.9 | 0.747 | 353 |
| LUAD: Lung adenocarcinoma, *: Chromosome, a: chromosome starting site of the gene, b: chromosome ending site of the gene. | | | | | | |

| Table S18. The correlation between BRIP1 and other genes of PRAD from the TCGA database | | | | | | |
| --- | --- | --- | --- | --- | --- | --- |
| Genes | Chr^*^ | Start^a^ | End^b^ | -Log10 (p) | Correlation | Samples |
| WDHD1 | 14 | 55405656 | 55493819 | 74.2 | 0.8 | 333 |
| PRR11 | 17 | 57232860 | 57284070 | 70.9 | 0.79 | 333 |
| CASC5 | 15 | 40886447 | 40954881 | 70.1 | 0.79 | 333 |
| CLSPN | 1 | 36197713 | 36235551 | 58.3 | 0.74 | 333 |
| DTL | 1 | 212208919 | 212278187 | 57.9 | 0.74 | 333 |
| MCM4 | 8 | 48872763 | 48890719 | 56 | 0.73 | 333 |
| KIF14 | 1 | 200520625 | 200589862 | 55.1 | 0.73 | 333 |
| CKAP2L | 2 | 113495444 | 113522254 | 55 | 0.73 | 333 |
| RBL1 | 20 | 35626178 | 35724410 | 53.2 | 0.72 | 333 |
| GAS2L3 | 12 | 100967489 | 101018685 | 52.8 | 0.72 | 333 |
| C11orf82 | 11 | 82612737 | 82669319 | 51.1 | 0.71 | 333 |
| TOP2A | 17 | 38544773 | 38574202 | 51 | 0.71 | 333 |
| PLK4 | 4 | 128802016 | 128820377 | 50.3 | 0.7 | 333 |
| KIF20B | 10 | 91461367 | 91534700 | 50 | 0.7 | 333 |
| E2F7 | 12 | 77415026 | 77459360 | 50 | 0.7 | 333 |
| KIF11 | 10 | 94352825 | 94415152 | 49.4 | 0.7 | 333 |
| INCENP | 11 | 61891445 | 61920635 | 49.4 | 0.7 | 333 |
| ESCO2 | 8 | 27632058 | 27670141 | 48.6 | 0.69 | 333 |
| RACGAP1 | 12 | 50382945 | 50419307 | 48.2 | 0.69 | 333 |
| SGOL1 | 3 | 20202085 | 20227724 | 48.1 | 0.69 | 333 |
| POLQ | 3 | 121150273 | 121264853 | 47.8 | 0.69 | 333 |
| MKI67 | 10 | 129894925 | 129924468 | 47.1 | 0.69 | 333 |
| NUP155 | 5 | 37291941 | 37371197 | 47 | 0.69 | 333 |
| CENPF | 1 | 214776532 | 214837914 | 46.3 | 0.68 | 333 |
| ERCC6L | X | 71424507 | 71458858 | 45.7 | 0.68 | 333 |
| BUB1 | 2 | 111395409 | 111435684 | 45.6 | 0.68 | 333 |
| EXO1 | 1 | 242011493 | 242053241 | 45.4 | 0.68 | 333 |
| HELLS | 10 | 96305543 | 96361856 | 45 | 0.68 | 333 |
| GINS1 | 20 | 25388323 | 25429191 | 44.9 | 0.67 | 333 |
| SMC2 | 9 | 106856541 | 106903700 | 44.5 | 0.67 | 333 |
| FANCI | 15 | 89787194 | 89860362 | 44.5 | 0.67 | 333 |
| BUB1B | 15 | 40453210 | 40513337 | 44.5 | 0.67 | 333 |
| TMEM48 | 1 | 54231134 | 54304225 | 44 | 0.67 | 333 |
| MCM10 | 10 | 13203554 | 13253104 | 43.8 | 0.67 | 333 |
| DLGAP5 | 14 | 55614834 | 55658396 | 43.8 | 0.67 | 333 |
| SKP2 | 5 | 36152145 | 36184142 | 43.6 | 0.67 | 333 |
| KIF18A | 11 | 28042163 | 28129746 | 43.4 | 0.67 | 333 |
| SMC4 | 3 | 160117430 | 160152741 | 43.1 | 0.66 | 333 |
| CENPE | 4 | 104026963 | 104119566 | 42.4 | 0.66 | 333 |
| NCAPG2 | 7 | 158424003 | 158497520 | 42.3 | 0.66 | 333 |
| TOPBP1 | 3 | 133319449 | 133380737 | 42.3 | 0.66 | 333 |
| KNTC1 | 12 | 123011809 | 123110947 | 41.9 | 0.66 | 333 |
| TMPO | 12 | 98909351 | 98944157 | 41.7 | 0.66 | 333 |
| NUSAP1 | 15 | 41624892 | 41673248 | 41.2 | 0.65 | 333 |
| XRCC2 | 7 | 152343587 | 152373250 | 40.6 | 0.65 | 333 |
| CDCA2 | 8 | 25316513 | 25365425 | 40.3 | 0.65 | 333 |
| KLHDC10 | 7 | 129710349 | 129775560 | 39.8 | 0.64 | 333 |
| ECT2 | 3 | 172468532 | 172539263 | 39.8 | 0.64 | 333 |
| ZNF829 | 19 | 37379026 | 37407193 | 39.7 | 0.64 | 333 |
| ZNF367 | 9 | 99148225 | 99180669 | 39.7 | 0.64 | 333 |
| UHRF1 | 19 | 4909510 | 4962165 | 39.7 | 0.64 | 333 |
| HMMR | 5 | 162887517 | 162918953 | 39.7 | 0.64 | 333 |
| ZNF107 | 7 | 64126511 | 64171401 | 39.4 | 0.64 | 333 |
| NUP205 | 7 | 135242662 | 135333499 | 39.1 | 0.64 | 333 |
| ZNF623 | 8 | 144718190 | 144735900 | 38.9 | 0.64 | 333 |
| ESPL1 | 12 | 53662083 | 53687427 | 38.8 | 0.64 | 333 |
| ORC1 | 1 | 52838501 | 52870143 | 38.7 | 0.64 | 333 |
| SGOL2 | 2 | 201390865 | 201448818 | 38.7 | 0.64 | 333 |
| KIAA1524 | 3 | 108268718 | 108308491 | 38.5 | 0.64 | 333 |
| CEP55 | 10 | 95256369 | 95288849 | 38.5 | 0.64 | 333 |
| ZMYM1 | 1 | 35544972 | 35581455 | 38.3 | 0.64 | 333 |
| CIT | 12 | 120123595 | 120315095 | 38.2 | 0.63 | 333 |
| RSBN1L | 7 | 77325743 | 77409120 | 38.2 | 0.63 | 333 |
| E2F2 | 1 | 23832920 | 23857712 | 37.9 | 0.63 | 333 |
| WDR76 | 15 | 44119112 | 44160617 | 37.9 | 0.63 | 333 |
| BRWD3 | X | 79924987 | 80065233 | 37.6 | 0.63 | 333 |
| RRM2 | 2 | 10262695 | 10271546 | 37.5 | 0.63 | 333 |
| FIGNL1 | 7 | 50511832 | 50518088 | 37.5 | 0.63 | 333 |
| KIF15 | 3 | 44803209 | 44894748 | 37.4 | 0.63 | 333 |
| CEP97 | 3 | 101443494 | 101486181 | 37.4 | 0.63 | 333 |
| RNF169 | 11 | 74459913 | 74553458 | 37.1 | 0.63 | 333 |
| NEIL3 | 4 | 178230991 | 178284092 | 37 | 0.63 | 333 |
| FANCM | 14 | 45605136 | 45670093 | 36.9 | 0.63 | 333 |
| CCNA2 | 4 | 122737599 | 122745088 | 36.8 | 0.63 | 333 |
| HEATR1 | 1 | 236712305 | 236767841 | 36.5 | 0.62 | 333 |
| CTDSPL2 | 15 | 44719579 | 44819429 | 36.3 | 0.62 | 333 |
| CEP128 | 14 | 80943330 | 81425828 | 36.3 | 0.62 | 333 |
| RASA2 | 3 | 141205926 | 141331197 | 36.2 | 0.62 | 333 |
| NCAPG | 4 | 17812525 | 17846487 | 36.1 | 0.62 | 333 |
| NCAPH | 2 | 97001484 | 97041274 | 35.9 | 0.62 | 333 |
| TPX2 | 20 | 30326904 | 30389603 | 35.6 | 0.62 | 333 |
| USP37 | 2 | 219314974 | 219433084 | 35.5 | 0.62 | 333 |
| MELK | 9 | 36572905 | 36677679 | 35.4 | 0.62 | 333 |
| KIF23 | 15 | 69706627 | 69740764 | 35.4 | 0.62 | 333 |
| DIAPH3 | 13 | 60239723 | 60738119 | 35.2 | 0.61 | 333 |
| HAUS6 | 9 | 19053141 | 19102902 | 35.2 | 0.61 | 333 |
| MRE11A | 11 | 94150469 | 94227040 | 35.1 | 0.61 | 333 |
| TNPO3 | 7 | 128594234 | 128695227 | 34.8 | 0.61 | 333 |
| ZNF619 | 3 | 40518604 | 40531728 | 34.7 | 0.61 | 333 |
| MED13 | 17 | 60019966 | 60142643 | 34.6 | 0.61 | 333 |
| TOR1AIP2 | 1 | 179809102 | 179846941 | 34.5 | 0.61 | 333 |
| KIAA1715 | 2 | 176790410 | 176867514 | 34.5 | 0.61 | 333 |
| PHF6 | X | 133507342 | 133562822 | 34.4 | 0.61 | 333 |
| LMNB1 | 5 | 126112315 | 126172712 | 34.4 | 0.61 | 333 |
| PARPBP | 12 | 102513956 | 102591298 | 34.2 | 0.61 | 333 |
| SENP1 | 12 | 48436757 | 48500091 | 34.1 | 0.61 | 333 |
| CENPL | 1 | 173768688 | 173793777 | 34.1 | 0.61 | 333 |
| NSD1 | 5 | 176560080 | 176727214 | 34 | 0.61 | 333 |
| DEPDC1B | 5 | 59892739 | 59995993 | 34 | 0.61 | 333 |
| ZNF81 | X | 47696301 | 47781655 | 33.9 | 0.6 | 333 |
| ZNF675 | 19 | 23835708 | 23870017 | 33.9 | 0.6 | 333 |
| CCDC75 | 2 | 37311594 | 37323738 | 33.9 | 0.61 | 333 |
| RSF1 | 11 | 77377274 | 77531880 | 33.8 | 0.6 | 333 |
| POLA1 | X | 24712058 | 25015102 | 33.8 | 0.6 | 333 |
| SKA1 | 18 | 47901392 | 47920538 | 33.7 | 0.6 | 333 |
| KIF4A | X | 69509879 | 69640774 | 33.7 | 0.6 | 333 |
| CDK13 | 7 | 39989959 | 40136733 | 33.5 | 0.6 | 333 |
| RC3H2 | 9 | 125611732 | 125667562 | 33.4 | 0.6 | 333 |
| KIAA0947 | 5 | 5422807 | 5490338 | 33.4 | 0.6 | 333 |
| SMC6 | 2 | 17845079 | 17981462 | 33.3 | 0.6 | 333 |
| CXADR | 21 | 18885224 | 18965897 | 33.3 | 0.6 | 333 |
| BRCA2 | 13 | 32889617 | 32973809 | 67.4 | 0.78 | 333 |
| BRCA1 | 17 | 41196312 | 41322420 | 52.1 | 0.71 | 333 |
| BLM | 15 | 91260579 | 91358686 | 50.4 | 0.7 | 333 |
| ATAD5 | 17 | 29159023 | 29222295 | 55.4 | 0.73 | 333 |
| ASPM | 1 | 197053257 | 197115824 | 57.7 | 0.74 | 333 |
| ARHGAP11B | 15 | 30916697 | 30977810 | 49.7 | 0.7 | 333 |
| ARHGAP11A | 15 | 32907345 | 32931868 | 57.8 | 0.74 | 333 |
| ANLN | 7 | 36429432 | 36493400 | 51.3 | 0.71 | 333 |
| ANKIB1 | 7 | 91875548 | 92030698 | 37.7 | 0.63 | 333 |
| PRAD: Prostate adenocarcinoma, *: Chromosome, a: chromosome starting site of the gene, b: chromosome ending site of the gene. | | | | | | |

| Table S19. The correlation between BRIP1 and other genes of STAD from the TCGA database | | | | | | |
| --- | --- | --- | --- | --- | --- | --- |
| Genes | Chr^*^ | Start^a^ | End^b^ | -Log10 (p) | Correlation | Samples |
| WDHD1 | 14 | 55405668 | 55493823 | 54 | 0.779 | 262 |
| E2F7 | 12 | 77415027 | 77459360 | 53.5 | 0.777 | 262 |
| KIF11 | 10 | 94353043 | 94415150 | 52.4 | 0.772 | 262 |
| HELLS | 10 | 96305547 | 96373662 | 52 | 0.77 | 262 |
| STIL | 1 | 47715811 | 47779819 | 50.9 | 0.765 | 262 |
| CDC7 | 1 | 91966408 | 91991321 | 49.9 | 0.76 | 262 |
| KNTC1 | 12 | 123011793 | 123110943 | 49.5 | 0.759 | 262 |
| POLQ | 3 | 121150278 | 121264853 | 49.3 | 0.757 | 262 |
| EZH2 | 7 | 148504475 | 148581413 | 48.8 | 0.755 | 262 |
| RFC5 | 12 | 118451393 | 118470935 | 47.6 | 0.749 | 262 |
| FBXO5 | 6 | 153291664 | 153304714 | 47.1 | 0.746 | 262 |
| ZNF367 | 9 | 99148223 | 99180611 | 46.9 | 0.745 | 262 |
| PLK4 | 4 | 128802016 | 128820350 | 46.5 | 0.743 | 262 |
| RRM1 | 11 | 4115937 | 4160106 | 46.4 | 0.742 | 262 |
| NOL11 | 17 | 65713949 | 65740647 | 46.2 | 0.741 | 262 |
| PARPBP | 12 | 102513956 | 102591298 | 46.1 | 0.741 | 262 |
| DTL | 1 | 212208919 | 212280742 | 45.9 | 0.74 | 262 |
| NCAPG | 4 | 17812525 | 17846485 | 45.8 | 0.739 | 262 |
| DNA2 | 10 | 70173821 | 70231879 | 45.8 | 0.739 | 262 |
| PRIM1 | 12 | 57125380 | 57146157 | 45.4 | 0.737 | 262 |
| CHEK1 | 11 | 125495036 | 125546150 | 44.9 | 0.734 | 262 |
| CEP78 | 9 | 80850978 | 80894606 | 44.8 | 0.734 | 262 |
| KIF15 | 3 | 44803209 | 44914868 | 44.7 | 0.733 | 262 |
| FANCI | 15 | 89787180 | 89860492 | 43.8 | 0.728 | 262 |
| RAD51AP1 | 12 | 4647950 | 4669214 | 43.7 | 0.727 | 262 |
| WDR76 | 15 | 44119161 | 44160617 | 43.4 | 0.726 | 262 |
| BUB1B | 15 | 40453224 | 40513337 | 43.2 | 0.725 | 262 |
| TMEM48 | 1 | 54231134 | 54304225 | 42.6 | 0.721 | 262 |
| ORC1 | 1 | 52838501 | 52870131 | 42.3 | 0.719 | 262 |
| USP1 | 1 | 62901968 | 62917475 | 42.2 | 0.719 | 262 |
| SRSF1 | 17 | 56080721 | 56084707 | 42.1 | 0.718 | 262 |
| C4orf21 | 4 | 113460492 | 113558151 | 41.9 | 0.717 | 262 |
| INTS2 | 17 | 59942731 | 60005377 | 41.8 | 0.716 | 262 |
| CASC5 | 15 | 40886218 | 40956540 | 41.8 | 0.716 | 262 |
| TMPO | 12 | 98909290 | 98944157 | 41.6 | 0.715 | 262 |
| KIF18A | 11 | 28042167 | 28129855 | 41.5 | 0.714 | 262 |
| NCAPD3 | 11 | 134020014 | 134095348 | 41.4 | 0.714 | 262 |
| RACGAP1 | 12 | 50370706 | 50426919 | 41.2 | 0.712 | 262 |
| NCAPG2 | 7 | 158424003 | 158497520 | 41.1 | 0.712 | 262 |
| MKI67 | 10 | 129894923 | 129924649 | 40.9 | 0.711 | 262 |
| CLSPN | 1 | 36185819 | 36235568 | 40.8 | 0.71 | 262 |
| NUP107 | 12 | 69080514 | 69136785 | 40.5 | 0.709 | 262 |
| GINS3 | 16 | 58328984 | 58440048 | 40.3 | 0.707 | 262 |
| MSH2 | 2 | 47630108 | 47789450 | 39.5 | 0.702 | 262 |
| SMC2 | 9 | 106856541 | 106903698 | 39.5 | 0.702 | 262 |
| RAD54L | 1 | 46713360 | 46744145 | 39.4 | 0.701 | 262 |
| MAD2L1 | 4 | 120976763 | 120988229 | 39.4 | 0.702 | 262 |
| DLGAP5 | 14 | 55614830 | 55658396 | 39.2 | 0.7 | 262 |
| C15orf42 | 15 | 90118818 | 90171253 | 39.1 | 0.699 | 262 |
| TOPBP1 | 3 | 133317019 | 133380737 | 39.1 | 0.7 | 262 |
| SRSF2 | 17 | 74730197 | 74733456 | 39.1 | 0.699 | 262 |
| MMS22L | 6 | 97590037 | 97731093 | 39.1 | 0.7 | 262 |
| E2F8 | 11 | 19245610 | 19263167 | 39.1 | 0.7 | 262 |
| RRM2 | 2 | 10262455 | 10271545 | 38.7 | 0.697 | 262 |
| PRIM2 | 6 | 57179603 | 57513375 | 38.6 | 0.696 | 262 |
| CENPK | 5 | 64813593 | 64858998 | 38.5 | 0.696 | 262 |
| NUP50 | 22 | 45559722 | 45583896 | 38.4 | 0.695 | 262 |
| ESCO2 | 8 | 27629466 | 27670157 | 38.4 | 0.695 | 262 |
| DNMT1 | 19 | 10244021 | 10341962 | 37.9 | 0.692 | 262 |
| CENPE | 4 | 104026963 | 104119566 | 37.8 | 0.691 | 262 |
| KIF23 | 15 | 69706585 | 69740764 | 37.7 | 0.691 | 262 |
| FANCD2 | 3 | 10068098 | 10143614 | 37.7 | 0.69 | 262 |
| NUSAP1 | 15 | 41624892 | 41673248 | 37.5 | 0.689 | 262 |
| SRPK1 | 6 | 35800743 | 35889119 | 37.5 | 0.689 | 262 |
| XRCC2 | 7 | 152341864 | 152373250 | 37.3 | 0.688 | 262 |
| C11orf82 | 11 | 82611017 | 82669319 | 37.1 | 0.686 | 262 |
| CCNE2 | 8 | 95891998 | 95908906 | 37 | 0.686 | 262 |
| EXO1 | 1 | 242011269 | 242058450 | 36.9 | 0.685 | 262 |
| DHX9 | 1 | 182808504 | 182856886 | 36.9 | 0.685 | 262 |
| DBF4 | 7 | 87505531 | 87538856 | 36.9 | 0.685 | 262 |
| TTF2 | 1 | 117602925 | 117650075 | 36.9 | 0.685 | 262 |
| CHAF1B | 21 | 37757676 | 37791313 | 36.8 | 0.684 | 262 |
| CDCA2 | 8 | 25316513 | 25365436 | 36.8 | 0.685 | 262 |
| POLA1 | X | 24712036 | 25015103 | 36.8 | 0.684 | 262 |
| MELK | 9 | 36572859 | 36677678 | 36.8 | 0.684 | 262 |
| TIMELESS | 12 | 56810903 | 56843187 | 36.5 | 0.682 | 262 |
| PSMD12 | 17 | 65334032 | 65362743 | 36.5 | 0.682 | 262 |
| MPHOSPH9 | 12 | 123636867 | 123728561 | 36.4 | 0.682 | 262 |
| RBL1 | 20 | 35624752 | 35724398 | 36.2 | 0.68 | 262 |
| KPNB1 | 17 | 45726842 | 45762871 | 36.2 | 0.68 | 262 |
| SASS6 | 1 | 100549119 | 100598511 | 36.1 | 0.679 | 262 |
| POLE2 | 14 | 50110273 | 50155140 | 36.1 | 0.679 | 262 |
| ORC6 | 16 | 46723555 | 46732306 | 36 | 0.679 | 262 |
| MCM6 | 2 | 136597196 | 136633996 | 35.9 | 0.678 | 262 |
| BUB1 | 2 | 111395275 | 111435691 | 35.9 | 0.678 | 262 |
| NASP | 1 | 46049518 | 46084566 | 35.7 | 0.676 | 262 |
| SMC4 | 3 | 160117062 | 160152750 | 35.6 | 0.676 | 262 |
| POLE | 12 | 133200348 | 133263951 | 35.6 | 0.676 | 262 |
| CENPI | X | 100353178 | 100418670 | 35.6 | 0.676 | 262 |
| CDK1 | 10 | 62538089 | 62554610 | 35.6 | 0.676 | 262 |
| NAA25 | 12 | 112464500 | 112546826 | 35.5 | 0.675 | 262 |
| CIT | 12 | 120123595 | 120315095 | 35.5 | 0.675 | 262 |
| SUZ12 | 17 | 30264037 | 30328064 | 35.4 | 0.675 | 262 |
| SELM | 22 | 31500758 | 31516055 | 35.2 | -0.673 | 262 |
| GEN1 | 2 | 17935125 | 17966632 | 35.2 | 0.673 | 262 |
| PMCH | 12 | 102590237 | 102591623 | 35.1 | 0.672 | 262 |
| WHSC1 | 4 | 1873151 | 1983934 | 35 | 0.672 | 262 |
| NUPL1 | 13 | 25875662 | 25923938 | 34.8 | 0.67 | 262 |
| DHX15 | 4 | 24519064 | 24586173 | 34.8 | 0.67 | 262 |
| CDC45 | 22 | 19466982 | 19508135 | 34.6 | 0.669 | 262 |
| DEPDC1 | 1 | 68939835 | 68962904 | 34.6 | 0.669 | 262 |
| KIAA1524 | 3 | 108268716 | 108308491 | 34.4 | 0.667 | 262 |
| TTK | 6 | 80713604 | 80752244 | 34.4 | 0.667 | 262 |
| MCM4 | 8 | 48872745 | 48890720 | 34.4 | 0.667 | 262 |
| KIF14 | 1 | 200520628 | 200589862 | 34.3 | 0.666 | 262 |
| HMMR | 5 | 162887209 | 162918947 | 34.3 | 0.667 | 262 |
| CEP85 | 1 | 26560691 | 26605299 | 34.3 | 0.667 | 262 |
| SRSF3 | 6 | 36562145 | 36573377 | 34.2 | 0.666 | 262 |
| FANCM | 14 | 45605143 | 45670093 | 34.2 | 0.665 | 262 |
| ECT2 | 3 | 172468472 | 172539264 | 34.1 | 0.665 | 262 |
| DCLRE1A | 10 | 115594488 | 115614142 | 34.1 | 0.665 | 262 |
| TYMS | 18 | 657604 | 673578 | 34.1 | 0.665 | 262 |
| RMI1 | 9 | 86595626 | 86618985 | 34.1 | 0.665 | 262 |
| NEDD1 | 12 | 97301001 | 97347129 | 34.1 | 0.665 | 262 |
| RFWD3 | 16 | 74655292 | 74700779 | 34 | 0.664 | 262 |
| NCAPH | 2 | 97001525 | 97039583 | 34 | 0.664 | 262 |
| DEPDC1B | 5 | 59892739 | 59996017 | 34 | 0.664 | 262 |
| PCNA | 20 | 5095599 | 5107272 | 33.9 | 0.663 | 262 |
| HNRNPR | 1 | 23630264 | 23670829 | 33.9 | 0.663 | 262 |
| FANCB | X | 14861529 | 14891191 | 33.9 | 0.664 | 262 |
| CCNA2 | 4 | 122737599 | 122745087 | 33.9 | 0.664 | 262 |
| CENPF | 1 | 214776538 | 214837931 | 33.8 | 0.663 | 262 |
| DEK | 6 | 18224099 | 18265054 | 33.7 | 0.662 | 262 |
| CSTF3 | 11 | 33098734 | 33183917 | 33.7 | 0.662 | 262 |
| PRC1 | 15 | 91509270 | 91538859 | 33.4 | 0.66 | 262 |
| CKAP2L | 2 | 113493930 | 113522254 | 33.4 | 0.66 | 262 |
| CDC6 | 17 | 38443885 | 38459171 | 33.4 | 0.66 | 262 |
| GUF1 | 4 | 44680444 | 44702943 | 33.2 | 0.658 | 262 |
| XPO1 | 2 | 61704984 | 61765761 | 33.1 | 0.658 | 262 |
| PTGES3 | 12 | 57057127 | 57082159 | 33.1 | 0.657 | 262 |
| FAM111B | 11 | 58874658 | 58894883 | 33.1 | 0.657 | 262 |
| PPAT | 4 | 57259528 | 57301781 | 33 | 0.657 | 262 |
| PDS5A | 4 | 39824483 | 39979576 | 32.9 | 0.656 | 262 |
| NUP205 | 7 | 135242667 | 135333505 | 32.9 | 0.656 | 262 |
| KIF20B | 10 | 91461367 | 91534700 | 32.9 | 0.656 | 262 |
| C4orf46 | 4 | 159587831 | 159593407 | 32.9 | 0.656 | 262 |
| INTS7 | 1 | 212113741 | 212208884 | 32.7 | 0.654 | 262 |
| KIF4A | X | 69509879 | 69640682 | 32.6 | 0.653 | 262 |
| FEN1 | 11 | 61560109 | 61564716 | 32.6 | 0.654 | 262 |
| ZWILCH | 15 | 66797297 | 66842115 | 32.4 | 0.652 | 262 |
| RFC3 | 13 | 34392186 | 34540695 | 32.4 | 0.652 | 262 |
| ERCC6L | X | 71424510 | 71458897 | 32.4 | 0.652 | 262 |
| CCDC138 | 2 | 109403213 | 109501933 | 32.4 | 0.652 | 262 |
| CKAP5 | 11 | 46764598 | 46867847 | 32.3 | 0.651 | 262 |
| UHRF1 | 19 | 4903092 | 4962165 | 32.2 | 0.651 | 262 |
| MCM10 | 10 | 13203554 | 13253104 | 32.2 | 0.651 | 262 |
| CPSF6 | 12 | 69633317 | 69668138 | 32.2 | 0.651 | 262 |
| BUB3 | 10 | 124913793 | 124924886 | 32.2 | 0.651 | 262 |
| PBK | 8 | 27667137 | 27695612 | 32.1 | 0.65 | 262 |
| BRCA2 | 13 | 32889611 | 32973805 | 36.6 | 0.683 | 262 |
| BRCA1 | 17 | 41196312 | 41277500 | 40.8 | 0.71 | 262 |
| BC035392 | 5 | 162909610 | 162921064 | 34.7 | 0.67 | 262 |
| BARD1 | 2 | 215590370 | 215674428 | 33.8 | 0.662 | 262 |
| ATAD5 | 17 | 29158988 | 29222887 | 42.8 | 0.722 | 262 |
| ATAD2 | 8 | 124332090 | 124428590 | 37.5 | 0.689 | 262 |
| ASPM | 1 | 197053258 | 197115824 | 36.5 | 0.682 | 262 |
| ARHGAP11A | 15 | 32907345 | 32932150 | 42.9 | 0.723 | 262 |
| ANKRD32 | 5 | 93954052 | 94075141 | 33.1 | 0.658 | 262 |
| STAD: Stomach adenocarcinoma, *: Chromosome, a: chromosome starting site of the gene, b: chromosome ending site of the gene. | | | | | | |
